# SNARE Complexity in Arbuscular Mycorrhizal Symbiosis

**DOI:** 10.3389/fpls.2020.00354

**Published:** 2020-04-03

**Authors:** Rik Huisman, Jan Hontelez, Ton Bisseling, Erik Limpens

**Affiliations:** Department of Plant Sciences, Laboratory of Molecular Biology, Wageningen University & Research, Wageningen, Netherlands

**Keywords:** SNARE, arbuscular mycorrhiza, syntaxin, exocytosis, VAMP, *Medicago*, membrane, symbiosis

## Abstract

How cells control the proper delivery of vesicles and their associated cargo to specific plasma membrane (PM) domains upon internal or external cues is a major question in plant cell biology. A widely held hypothesis is that expansion of plant exocytotic machinery components, such as SNARE proteins, has led to a diversification of exocytotic membrane trafficking pathways to function in specific biological processes. A key biological process that involves the creation of a specialized PM domain is the formation of a host–microbe interface (the peri-arbuscular membrane) in the symbiosis with arbuscular mycorrhizal fungi. We have previously shown that the ability to intracellularly host AM fungi correlates with the evolutionary expansion of both v- (VAMP721d/e) and t-SNARE (SYP132α) proteins, that are essential for arbuscule formation in *Medicago truncatula*. Here we studied to what extent the symbiotic SNAREs are different from their non-symbiotic family members and whether symbiotic SNAREs define a distinct symbiotic membrane trafficking pathway. We show that all tested SYP1 family proteins, and most of the non-symbiotic VAMP72 members, are able to complement the defect in arbuscule formation upon knock-down/-out of their symbiotic counterparts when expressed at sufficient levels. This functional redundancy is in line with the ability of all tested v- and t-SNARE combinations to form SNARE complexes. Interestingly, the symbiotic t-SNARE SYP132α appeared to occur less in complex with v-SNAREs compared to the non-symbiotic syntaxins in arbuscule-containing cells. This correlated with a preferential localization of SYP132α to functional branches of partially collapsing arbuscules, while non-symbiotic syntaxins accumulate at the degrading parts. Overexpression of VAMP721e caused a shift in SYP132α localization toward the degrading parts, suggesting an influence on its endocytic turn-over. These data indicate that the symbiotic SNAREs do not selectively interact to define a symbiotic vesicle trafficking pathway, but that symbiotic SNARE complexes are more rapidly disassembled resulting in a preferential localization of SYP132α at functional arbuscule branches.

## Introduction

The growth and maintenance of eukaryotic cells requires the continuous delivery of vesicles to the plasma membrane (PM); exocytosis. The fusion of vesicles with their target membrane is driven by the interaction of vesicle SNAREs (v-SNAREs or R SNAREs) on the vesicle with a complex of target membrane SNAREs (t-SNAREs) on the target membrane. A t-SNARE complex consists of a Qa, Qb, and Qc SNARE that each contribute a single SNARE domain, or a Qa and Qb+Qc SNARE, of which the latter contributes two SNARE domains to the complex (Sanderfoot, 2007). In plants, the number of SNAREs involved in exocytosis has expanded to be encoded by families of at least partially redundant proteins (Sanderfoot, 2007). This suggests that expansion of SNARE proteins allowed the adaptation of exocytosis to these different biological processes. Furthermore, the expansion of the number of secretory SNAREs in plants has been suggested to allow the presence of multiple exocytosis pathways in one cell (Sanderfoot, 2007; Łangowski et al., 2016; Kanazawa and Ueda, 2017). We define an exocytosis pathway as the traffic and fusion of a distinct population of vesicles and associated cargo to the PM or subdomain. An example of the use of different exocytosis pathways to create specialized membrane domains can be found in animal cells: in mammalian polarized epithelial cells two PM domains with a distinct protein composition are present; an apical domain and a basolateral domain (Mostov et al., 2003). Trafficking of proteins to these domains is mediated by distinct populations of vesicles, which depend on distinct v-SNAREs (Martinez-Arca et al., 2003) and different t-SNAREs that are present at the two domains (Kreitzer et al., 2003). Whether SNAREs mark distinct exocytotic trafficking pathways in plants as well is currently unknown, although differential effects of syntaxins on secretion have been reported (Kalde et al., 2007; Leucci et al., 2007; Rehman et al., 2008; Waghmare et al., 2018).

A key example of a biological process that depends on specific SNARE proteins is the formation of the peri-arbuscular membrane (PAM) during the endosymbiotic interaction of plants with arbuscular mycorrhizal (AM) fungi (Harrison and Ivanov, 2017). AM fungi colonize the roots of almost all land plants, where they form highly branched hyphal structures in cortical cells, called arbuscules, that are surrounded by the specialized PAM, which creates a symbiotic interface where exchange of nutrients takes place (Gutjahr and Parniske, 2013). Upon entering a cortical cell, first a trunk domain is established, after which the fungus undergoes repeated dichotomous branching by which gradually finer branches appear. These fine branches are characterized by the absence of a structured cell wall and contain specific plant proteins, such as symbiotic phosphate and lipid transporters to control the exchange of nutrients, that distinguish it from the PM which is continuous to the PAM. As such the PAM represents a specialized PM subdomain that involves the polar targeting of membrane vesicles (Pumplin and Harrison, 2009).

It has previously shown that the formation of arbuscules in *Medicago truncatula* (Medicago) depends on the two v-SNAREs MtVAMP721d and MtVAMP721e, and on the Qa-type t-SNARE (syntaxin) isoform MtSYP132α (named MtSYP132A by Pan et al., 2016) which results from alternative splicing (Ivanov et al., 2012; Huisman et al., 2016; Pan et al., 2016). Silencing of MtVAMP721d/e by RNAi results in a phenotype where the formation of arbuscular branches is almost completely blocked (Ivanov et al., 2012). RNAi of MtSYP132α results in small arbuscules that collapse prematurely. The silencing of VAMP721d/e or SYP132α does not affect root development under the tested conditions, suggesting that they are dedicated to symbiosis. For simplicity, we will refer to these SNAREs as ‘symbiotic’ SNAREs while we will call other SNAREs ‘non-symbiotic,’ even though the latter ones may be involved in symbiosis as well. These symbiotic SNARE proteins are highly conserved in dicot plants that host AM fungi and are absent in non-host lineages, which is another strong indication that they are dedicated to symbiosis (Ivanov et al., 2012; Huisman et al., 2016; Pan et al., 2016). Since the formation of the PAM depends on both a specialized v- and t-SNARE, it is a particularly interesting model to study whether different SNAREs mark distinct exocytosis pathways in plant cells.

Therefore, we questioned what the difference is between symbiotic SNAREs and their non-symbiotic paralogs, to find out whether they can define a specialized symbiotic exocytosis pathway. We examined the potential functional redundancy between symbiotic SNARES and their closest non-symbiotic paralogs, as well as family members that are expressed in the same cell. Furthermore, we compared the spatiotemporal localization and SNARE-interactions of symbiotic and non-symbiotic SNAREs in arbuscule containing cells. Overall, our data indicate that the symbiotic SNAREs do not selectively interact to define a symbiotic vesicle trafficking pathway, but that symbiotic SNARE complexes are more rapidly disassembled. This results in a preferential localization of SYP132 at functional arbuscule branches, which may be instrumental to form an optimal functioning arbuscule.

## Results

### The Qa- and R-SNARE Repertoire of Arbuscule-Containing Cells

**FIGURE 1:**
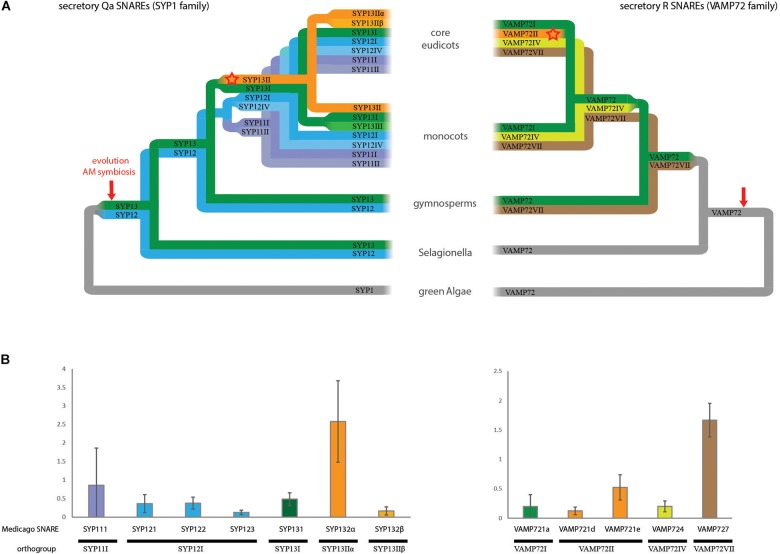
The SNARE repertoire of arbuscule containing cells. **(A)** Schematic phylogenetic trees showing the evolution of the exocytosis related SNARE families SYP1 and VAMP72. The trees are showing orthogroups instead of individual genes, indicated by the use of roman numbers. Orthogroups were named following Sanderfoot (2007). Both schematic trees are based on the actual phylogenetic trees shown in Supplementary Figures S1, S2. **(B)** Digital droplet PCR on cDNA from arbuscule containing cells. Only SYP1 and VAMP72 genes that are expressed in arbuscule-containing cells are shown. The copy in each cDNA was normalized against the average expression of all tested genes. Error bars represent standard deviation of three biological replicates.

To guide functional comparisons we first investigated the phylogenetic relationship of exocytosis-related SNAREs and determined their expression levels in arbuscule-containing cells in the model plant Medicago (*Medicago truncatula*). We focused on Qa t-SNAREs called syntaxins (SYP1 class) and the VAMP72 class R-SNAREs, as they form the core members of exocytosis-related SNARE complexes. We divided the individual SNARE genes into orthogroups among a wide range of plant species (Figure 1A and Supplementary Figures S1, S2). This classification allowed comparison of our data with studies on SNARE interactions in other plant species (Supplementary Table S1). Furthermore, comparing SNARE genes from separate orthogroups is most likely to reveal functional specialization, since we expect that there will be a strong selective pressure to maintain functionally different paralogs during evolution, resulting in conserved orthogroups.

The vast expansion of the number of syntaxins occurs at the base of the angiosperms: whereas we found only two conserved orthogroups in the gymnosperms, this number increases to six orthogroups in angiosperms, including the SYP13II orthogroup that we linked to symbiosis earlier (Huisman et al., 2016). As shown before, during evolution this orthogroup is strictly linked to the ability of plant species to interact with AM fungi (Bravo et al., 2016; Huisman et al., 2016). It is conserved in all analyzed AM host plant species (16/16), and lost in all (6/6) analyzed plant species that (independently) lost the ability to interact with AM fungi. In the dicot lineage, SYP13II is spliced into two different transcripts encoding the SYP13IIα and SYP13Iiβ proteins.

Within the VAMP72 family, four orthogroups can be found. Most individual VAMP genes of both Arabidopsis and Medicago are the result of independent and recent expansions within the VAMP72I orthogroup. The symbiotic MtVAMP721d and MtVAMP721e genes form a clear group together with other VAMP genes from dicots embedded within the VAMP72I orthogroup. We named this group VAMP72II. VAMP72II does not contain monocot genes, nor genes from *Aquilegia coerulea*, the most basal sequenced eudicot. Thus, the symbiotic VAMPs likely evolved at the base of the dicot lineage, after the split of *A. coerulea*, coinciding with the evolution of alternative splicing of SYP132. The conservation of VAMP72II is largely, but not strictly correlated to the ability of plants to host AM fungi. In particular, it is conserved in 1 out of 5 of the analyzed dicot AM non-host plants (*Striga hermonthica*), while it is lost in 2 out of 10 analyzed dicot AM host plants (*Manihot esculenta* and *Carica papaya*).

To get an accurate overview of the relative expression of symbiotic SNAREs and their orthologs in arbuscule-containing cells, we isolated RNA from these cells using laser microdissection. We used digital droplet PCR (ddPCR) to measure the absolute levels of transcripts of the different SNAREs that showed expression in this cell-type based on qPCR analysis (Huisman et al., 2016). ddPCR allows a more reliable quantitative measurement of expression levels compared to our qPCR approached used earlier (Huisman et al., 2016), as it is not affected by differences in primer efficiencies (Hindson et al., 2013). This analysis confirmed that multiple SNAREs are expressed in arbuscule-containing cells, and showed that MtSYP132α is clearly the dominant syntaxin (Figure 1B). Among the VAMPs, *MtVAMP727* is the most highly abundant transcript in arbuscule-containing cells, followed by the symbiotic *MtVAMP721e*.

Based on phylogeny and expression in arbuscule-containing cells we selected SNAREs for functional comparison. We selected the highest expressed symbiotic VAMP MtVAMP721e, along with the expressed genes of all other orthogroups; MtVAMP721a, MtVAMP724, and MtVAMP727. We selected both spliceforms of the symbiotic SYP13II; MtSYP132α and MtSYP132β, and their closest non-symbiotic paralog SYP131, all of which are expressed in arbuscule-containing cells. Further, we selected the more distantly related MtSYP121, which is also expressed in arbuscule-containing cells (Figure 1B).

### Most Exocytosis Related SNAREs Locate to the Peri-Arbuscular Membrane

**FIGURE 2:**
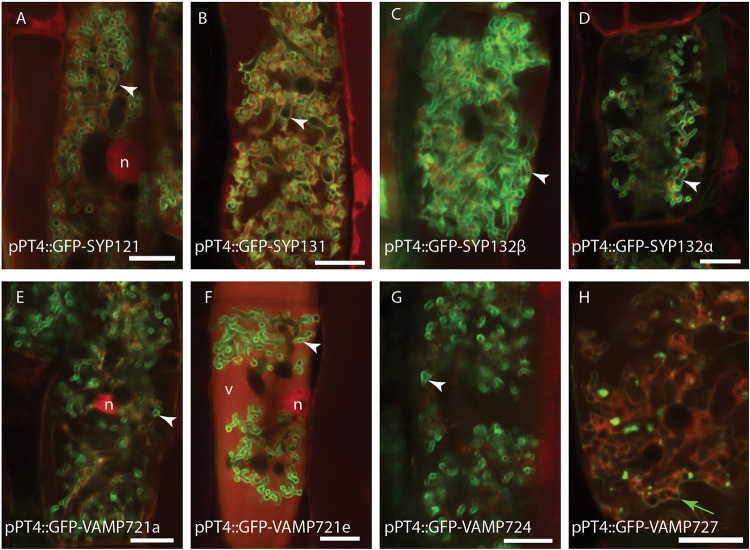
Intracellular localization of syntaxins **(A–D)** and VAMPs **(E–H)** in arbuscule containing cells. Different SNAREs fused to GFP were expressed from the arbuscule specific Medicago PT4 promoter, combined with dsRed that marks the cytoplasm and nucleus (n), as well as occasional accumulation in the vacuoles (v). white arrowheads indicate the PAM, a green arrow indicates the tonoplast. Scalebars are 10 μm.

We first investigated whether the different SNARE proteins can localize to the PAM. Therefore, we expressed the different SNAREs fused to GFP from the Medicago PT4 promoter, which is exclusively active in arbuscule-containing cells, and determined their localization by confocal microscopy. All syntaxins localized to the PAM (Figures 2A–D), as shown earlier for SYP132α, SYP132β, and SYP121 when expressed from their native promoter (Huisman et al., 2016). Even though v-SNAREs function on vesicles, overexpression typically results in their accumulation on the target membrane (Kwaaitaal et al., 2010; Ivanov et al., 2012). MtVAMP721a, MtVAMP721e, and MtVAMP724 accumulated on the PAM (Figures 2E–G). In contrast MtVAMP727 accumulated in punctuate intracellular compartments, as well as on membranes enclosing multiple arbuscular branches and the tonoplast (Figure 2H).

### Interaction Between v- and t-SNAREs

**FIGURE 3:**
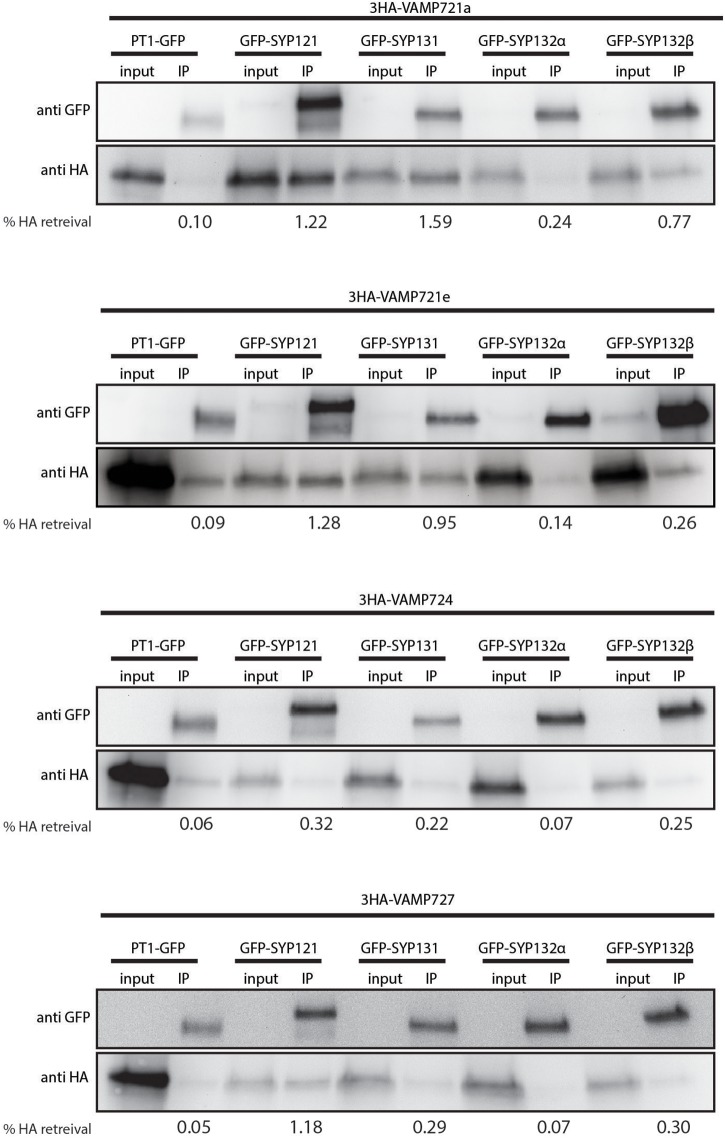
Co-immunoprecipitation analysis of SNARE interactions. Western blot showing GFP-labeled syntaxins or negative control PT1, and 3HA-labeled VAMPs in extracts of mycorrhized Medicago roots before (input) and after immunoprecipitation (IP) using anti-GFP coated beads. The fraction (% HA retrieval) of HA-labeled VAMPs that co-purified with the GFP-labeled bait proteins was quantified as the intensity of the bands in the IP lane, divided by the intensity of the corresponding band in the input lane, and corrected for the volume difference of the two fractions.

Next, we tested whether there is specificity in SNARE-SNARE interactions at the PAM, by using a co-immunoprecipitation (co-IP) approach. Therefore, we expressed combinations of GFP-labeled syntaxins and triple HA-tag labeled VAMPs from the PT4 promoter in mycorrhized Medicago roots. As a negative control we tested the interaction with the PAM-localized GFP-tagged MtPT1 (Pumplin et al., 2012). The GFP-tagged proteins were immunoprecipitated from root extracts, and the co-IP of the VAMPs was determined by western blot using an antibody against the HA-tag (Figure 3). The experiment was performed twice with similar results (Supplementary Figure S3). We noticed that a small amount of HA-labeled VAMPs co-purified with negative control PT1, likely reflecting a weak background of non-solubilized proteins. For all combinations of syntaxins and VAMPs, the anti-GFP blot revealed two bands of around 63 and 25 kDa, representing the SYP-GFP fusion protein and free GFP respectively. On the anti-HA blot, one clear band around 30 kDa was visible for all input fractions corresponding to the 3HA-VAMP fusion proteins. For all combinations, a band was also visible in the IP lane representing the 3HA-VAMP fusion proteins that co-purified with the GFP labeled SYPs. We quantified the fraction of HA-labeled VAMPs from the input samples that was retrieved in the IP samples as a proxy for the amount of interaction between VAMPs and syntaxins. In general, the individual SNAREs showed different co-IP levels: SYP121 and SYP131 appeared to interact stronger than both spliceforms of SYP132, as indicated by the relative signals after co-IP compared to the input fractions in two independent replicate experiments. VAMP724 interacted less than the other VAMPs. Most striking, in case of SYP132α the co-IP of all VAMPs was extremely weak with signals barely detectable, similar to that observed for the non-interacting control protein MtPT1. To further confirm the ability of SYP132α to interact with VAMPs we used a BiFC approach in *Nicotiana benthamiana* leaves. This showed that SYP132α was able to interact with all tested VAMPs; VAMP721a, VAMP721d, and VAMP721e (Supplementary Figure S4). Thus, we consider it most likely that SYP132α can interact with all VAMPs. The lower levels of VAMPs that co-purify with SYP132α may indicate that these complexes are more rapidly disassembled.

### Symbiotic SNAREs Are Largely Redundant With Their Non-symbiotic Paralogs

**FIGURE 4:**
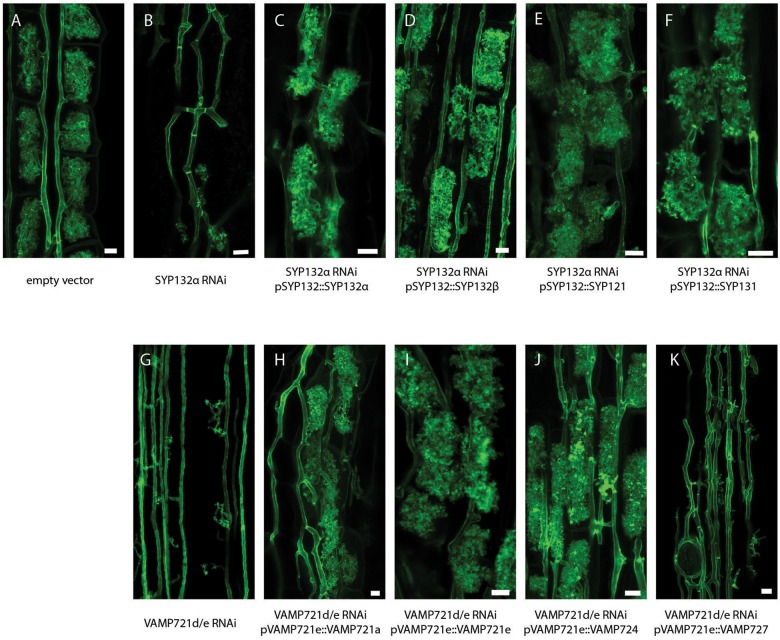
RNAi complementation analysis. Projection of confocal image stacks showing AM fungi in Medicago roots, stained with wheat germ agglutinin conjugated to alexa488 *n* = 3–4 biological replicates. **(A)** Empty vector control, **(B)** RNAi of SYP132α, **(C–F)** complementation of SYP132 RNAi with other syntaxins, **(G)** RNAi of VAMP721d and -e, and **(H–K)** complementation of VAMP721d/e RNAi with other VAMPs.

Previously, we already showed that SYP132β can restore arbuscule formation upon knock-down of SYP132α when expressed at sufficient levels (Huisman et al., 2016). To determine whether this also holds for the other non-symbiotic syntaxins we used a similar RNAi complementation approach. Therefore, we combined in one binary vector the RNAi constructs targeting SYP132α or VAMP721d/e (Ivanov et al., 2012; Huisman et al., 2016), and expression cassettes expressing the non-symbiotic SNAREs from the promoter of their symbiotic family member. As positive controls, we expressed VAMP721e lacking its native 3′-UTR or codon-scrambled SYP132α, both of which escape silencing by the RNAi constructs. We used *Agrobacterium rhizogenes* mediated transformation to generate transgenic roots expressing these constructs. 6 weeks after inoculation with AM fungi, roots were harvested and successful RNAi was confirmed in each individual root by qRT-PCR (Supplementary Figure S5). Next, we quantified the arbuscule development and level of colonization in the silenced roots and determined the arbuscule phenotype by confocal microscopy after staining with WGA-Alexa488 (Figures 4A–K and Supplementary Figure S6). Knockdown of *SYP132α* resulted in a severe reduction of mature arbuscules, as most arbuscules were stunted or collapsed. Expression of all of the tested syntaxins was sufficient to restore the arbuscule morphology of SYP132α knockdown to that of non-silenced control roots (Figures 4A–F), although complementation with SYP121 resulted in a slightly lower amount of arbuscules that fill the whole cell (Supplementary Figure S6A). In a similar way we tested the functional redundancy of VAMP members. The phenotype of VAMP721d/e RNAi is slightly stronger than SYP132α RNAi, as arbuscule formation does not proceed beyond the formation of the arbuscular trunk (Ivanov et al., 2012; Supplementary Figure S6B). After expression of VAMP721a, VAMP721e or VAMP724, the arbuscule phenotype was fully restored, showing many mature arbuscules (Figures 4A,G–J). In contrast, the arbuscule morphology was not restored after expression of VAMP727 (Figure 4K). The levels of mature arbuscules after complementation with VAMP721a or VAMP724 were slightly lower than the empty vector control, but not significantly different from the positive control; complementation with VAMP721e itself (Supplementary Figure S6B). It should be noted that the amount of biological replicates was relatively low which could mask subtle phenotypes. Nevertheless, these data show that most non-symbiotic SNAREs can functionally replace their symbiotic counterparts with respect to arbuscule morphology.

### Generation of *syp132α-1*, a Constitutively SYP132β-Splicing Mutant

**FIGURE 5:**
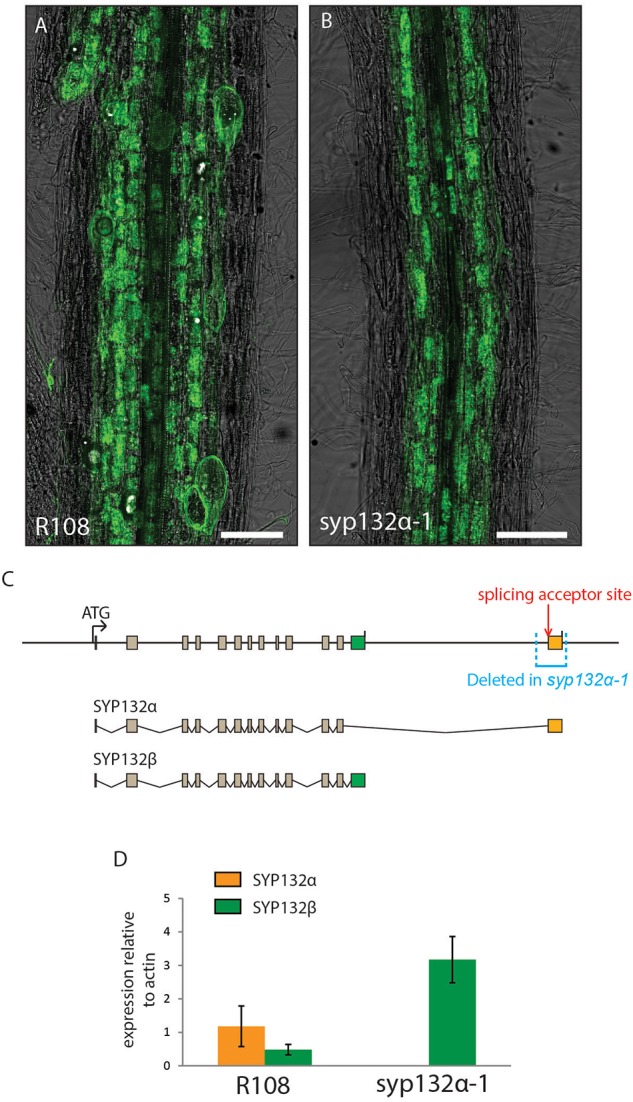
**(A,B)** Mycorrhized wild-type **(A)** and *syp132α-1* roots stained with WGA-Alexa488. Scale bars are 100 μm. **(C)** Schematic representation of the SYP132 exons in the genome of Medicago, the different spliced transcripts, and the region deleted in the *syp132α-1* mutant. **(D)** Digital droplet PCR measurements of the transcript levels of SYP132α and β in the arbuscule-containing cells of wild-type plants and *syp132α-1* plants. Error bars represent standard deviation of 4 (R108) or 3 (*syp132α-1*) biological replicates.

To rule out more subtle effects on arbuscule morphology that could go unnoticed in *A. rhizogenes* transformed roots, we generated a stable CRISPR line in which SYP132 is constitutively spliced into the SYP132*β* form. This line, named *syp132α-1*, contains a 532 bp deletion, which includes the entire α-specific exon as well as 34 bp of the last intron (Figure 5C). Since the deletion in this mutant includes the splice acceptor site in front of the *SYP132α*-specific last exon, we used digital droplet PCR on laser dissected arbuscule-containing cells to test whether a compensatory raise in the *β*-splice form occurs in arbuscule-containing cells. As shown in Figure 5D, the lack of *SYP132α* in the *SYP132α-1* mutant is indeed compensated by an equivalent increase of *SYP132β* levels. In this line we did not observe the premature degradation of arbuscules or impaired symbiosome development phenotype previously observed upon silencing of the *SYP132α* isoform. Instead, the formation of arbuscules as well as symbiosomes appeared similar to wild-type (Figures 5A,B and Supplementary Figure S7), in line with the RNAi complementations.

**FIGURE 6:**
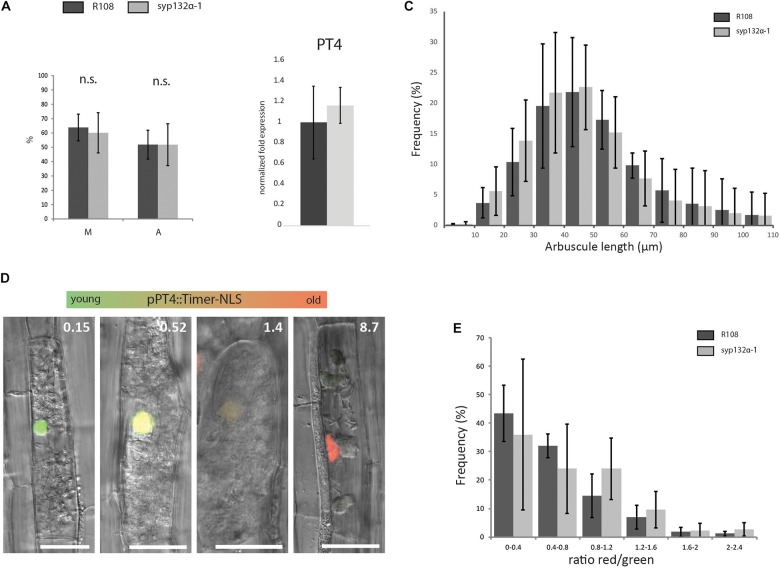
**(A)** Colonization (M%) and arbuscule abundance (A%) in wild-type and *syp132α-1* roots, 6 weeks after inoculation with *R. irregularis*. Parameters are scored according to Trouvelot et al. (1986). Error bars represent the standard deviation of five biological replicates. **(B)** qRT-PCR on cDNA isolated from mycorrhized roots of wild-type and *syp132α-1* showing the expression of marker gene PT4. Error bars represent the standard deviation of three biological replicates. **(C)** Arbuscule length distribution measured in wild-type and *syp132α-1* roots infected with *R. irregularis.* Around 1000 arbuscules per root were measured. Error bars represent standard deviation of four different roots. **(D)** Confocal laser scanning microscopy images overlaid with DIC images showing expression of nuclear localized dsRed-E5 (Timer) driven by the PT4 promoter in arbuscular cells of wild-type plants. The numbers in the upper-right corner indicate the ratio between red and green fluorescence. **(E)** Age distribution of arbuscules in wild-type and *syp132α-1* roots. The age of ∼100 arbuscules was measured per root. Collapsed arbuscules were not measured. Error bars represent standard deviation of four individual roots. **(A–C,E)** No significant differences between wild-type and syp132α-1 were found (Student’s *t*-test *p* ≤ 0.05).

This mutant now allowed us to study in more detail whether arbuscule morphology and lifetime were affected when the symbiotic SYP132α is replaced by its non-symbiotic isoform at native levels. We found that the level of colonization (M%) and arbuscule abundance (A%) in *syp132α-1* was identical to wild-type R108 plants (Figure 6A). This is further confirmed by the similar expression level of the arbuscule-specific marker PT4 in *syp132α-1* compared to wild-type (Figure 6B). Next, we quantified arbuscule size distribution in the *syp132α-1* mutant and wild-type, by measuring the arbuscule size in images of 1000 arbuscules per root of wild-type and mutant plants (*n* = 4). However, no differences in arbuscule size distribution were observed (Figure 6C). From the same images we quantified the fraction of collapsed arbuscules, which was not significantly different. To get more direct data on the lifetime of arbuscules, we developed a construct that expresses the fluorescent timer reporter protein dsRed-E5 fused to a nuclear localization tag from the PT4 promoter. DsRed-E5 slowly maturates from green to red fluorescence with a half-time of approximately 10 h (Mirabella et al., 2004). Since the PT4 promoter is switched on at the start of the formation of the PAM (Pumplin et al., 2012), the ratio of red/green fluorescence can be used as a proxy for arbuscule age. We expressed the pPT4::Timer-NLS construct in mycorrhized Medicago roots and determined the red/green ratio of the nucleus of approximately 100 arbuscular cells per root (*n* = 4). We observed a clear correlation between the color of the nucleus and the phenotype of the arbuscule: arbuscules with young (turgoid) branches that did not yet completely fill the cell displayed a low nuclear red/green ratio, while collapsed arbuscules displayed a high nuclear red/green ratio. Mature arbuscules displayed intermediate red/green ratio’s (Figure 6D). This confirmed the suitability of the construct to measure arbuscule age. The age distribution of arbuscule-containing cells in *syp132α-1* plants was not significantly different from the age distribution in wild-type plants (Figure 6E). These detailed analyses unequivocally show that SYP132β can functionally replace SYP132α to restore normal arbuscule morphology at native levels.

### SYP132α Turnover Is Affected by VAMP721e Expression

**FIGURE 7:**
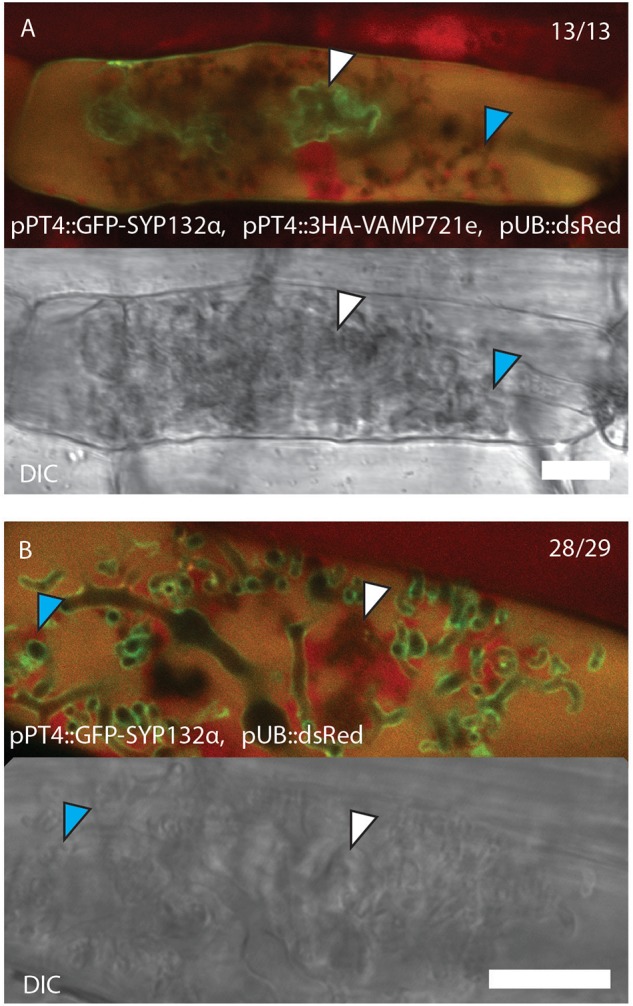
Confocal laser scanning microscopy images and accompanying bright-field images showing the localization of SYP132α fused to GFP with **(A)** and without **(B)** co-expression of triple HA-tag labeled VAMP721e, both driven by the PT4 promoter. Collapsed branches are marked by white arrowheads, functional branches are marked by blue arrowheads. The red signal shows autofluorescence of collapsed arbuscules and the nuclei and cytoplasm that are labeled with dsRed driven by the Arabidopsis ubiquitin 3 promoter. Scale bars are 10 μm.

Upon examining the co-expression of the different syntaxins and VAMPs used in the co-IP experiment, we noticed a different behavior of SYP132α compared to the non-symbiotic syntaxins upon co-expression with VAMP721e. Previously, we observed a difference in localization between the two SYP132 isoforms when arbuscules start to collapse, with SYP132α being more confined to the non-degrading (“functional”) arbuscule branches and SYP132β accumulating on degrading parts (Huisman et al., 2016). However, upon co-expression of SYP132α with VAMP721e both from the PT4 promoter, SYP132α localized mostly to the collapsed part of degrading arbuscules, resembling the behavior of SYP132β (Figure 7A and Supplementary Figure S8). This localization was observed in 13 out of 13 partially collapsing arbuscules, whereas without overexpression of VAMP721e, SYP132α preferentially localized to the functional branches in 28 out of 29 partially collapsing arbuscules (Figure 7B and Supplementary Figure S8). This indicates that overexpression of VAMP721e causes the accumulation of SYP132α on collapsing arbuscule domains. It suggests that VAMP721e levels affect the localization of SYP132α. This can be achieved in different ways; either by redirecting the newly synthesized proteins to the degrading arbuscule branches, or by increasing the turnover of SYP132α at the functional branches.

## Discussion

The expansion and evolutionary conservation of symbiotic v- and t-SNAREs in AM (eudicot) host plants suggested that these SNAREs may have specialized to mark a distinct exocytosis pathway to form a symbiotic host–microbe interface. Here we show that the essential role of the symbiotic SNAREs in interface formation, MtVAMP721d/e and MtSYP132α, can be largely explained by their dominant expression levels in arbuscule forming cells. We compared the interaction of a wide range of exocytosis related v- and t-SNAREs in a single cell-type. All tested v- and t-SNAREs that are expressed in arbuscule-containing cells were shown to be able to form complexes at the PAM. This, together with the ability of the majority of non-symbiotic SNARE paralogs to restore the arbuscule morphology defect upon loss of symbiotic SNAREs, shows that the symbiotic SNAREs do not mark a distinct exocytosis pathway that distinguishes traffic to the PAM from traffic to the PM. This is in line with the suggestion by Pumplin et al. (2012) that targeting to the PAM involves a transient reorientation of general secretion. Instead, our data suggest that the symbiotic SNARE complexes are more rapidly disassembled, most likely to confine SYP132α to functional branches.

The use of different SNAREs in specific biological processes most often relates to differences in the spatiotemporal expression and dynamics of these SNAREs (Kanazawa and Ueda, 2017; Slane et al., 2017; Supplementary Table S1). In Arabidopsis, different secretory t-SNAREs are expressed in different plant tissues (Enami et al., 2009), show specific subcellular localization patterns (Lauber et al., 1997; Collins et al., 2003; Ângelo Silva et al., 2010; Ichikawa et al., 2014), turnover (Reichardt et al., 2011) or dynamics (Nielsen and Thordal-Christensen, 2012). The only clear example of a plant SNARE that evolved to define a new trafficking pathway is VAMP727, which acquired the ability to interact with vacuolar t-SNAREs and marks vesicles with a distinguishable cargo (Ebine et al., 2011). The low levels of MtVAMP727 on the PAM suggest that most MtVAMP727-labeled vesicles are targeted to other compartments like endosomes and the vacuole. This explains the inability of MtVAMP727 to complement the arbuscule defect in VAMP721d/e RNAi roots, despite the observation that it is the most highly expressed v-SNARE in arbuscule-containing cells.

A striking observation from our co-IP analyses was the lower level of v-SNAREs in complexes with SYP132α in arbuscule-containing cells, compared to the non-symbiotic syntaxins. This might reflect a stricter regulation of SYP132α complexes by accessory factors. SNARE complexes occur in two different states; a *trans*-SNARE complex between the v-SNARE on the vesicle and the t-SNAREs on the target membrane, and a *cis*-SNARE complex where both v- and t-SNAREs are present on the same membrane until they are disassembled by the ATPase NSF and SNAPs (Südhof and Rothman, 2009). It was recently shown that, also before vesicle fusion, SNAREs involved in cytokinesis are transported to their site of action as *cis*-SNARE complexes, which may apply to other secretory SNAREs as well (Karnahl et al., 2017). The actual vesicle fusion event involving *trans-*SNARE complexes is short-lived compared to the lifetime of *cis*-SNARE complexes. Therefore, it is likely that the complexes that are detected in our co-IP study – or any of the previous studies on SNARE interactions in plants – are in fact mostly representing *cis*-SNARE complexes. This may be further exaggerated by the strong expression from the PT4 promoter. In this scenario, the amount of VAMPs that co-purify with syntaxins is not a measure for the amount of vesicle fusion events driven by this particular complex, but merely represents the speed of *cis*-complex formation and dissociation. This implies that SYP132α containing SNARE complexes are more rapidly disassembled, possibly to recycle the syntaxin for subsequent fusion reactions at the functional PAM branches or to facilitate the interaction with other proteins.

The lower amount of detected SYP132α containing SNARE complexes correlated with a different localization with respect to degrading arbuscule branches in comparison to the non-symbiotic syntaxins (Huisman et al., 2016). We speculate that the accumulation of SYP132 at the collapsing parts may be related to the structural resemblance of the collapsing arbuscule to the encasement of haustoria from filamentous pathogens, which occurs as result of a defense response toward these pathogens. During the encasement of haustoria multivesicular bodies (MVBs) are redirected toward the haustorial encasement. This results in the inclusion of the inner vesicles of MVB within the deposited cell-wall material as exosomes (Assaad et al., 2004; An et al., 2006; Micali et al., 2011). Following this route, endocytosed membrane proteins including the recycling t-SNARE SYP121 have been shown to accumulate in the encasement (Nielsen et al., 2012). From transmission electron microscopy images, it was shown that collapsed arbuscule branches are encased by depositions of cell-wall like material, with many paramural bodies (Cox and Sanders, 1974). In two recent detailed ultrastructural analyses of the arbuscule interface it was shown that extracellular vesicles and fusion of MVB’s also occurs at developing arbuscules, suggesting a tightly regulated process (Ivanov et al., 2019; Roth et al., 2019). Therefore, similar to the accumulation of SYP121 in the encasement of haustoria, we hypothesize that the accumulation of SYPs on collapsing arbuscules may represent a retargeting of MVB’s containing endocytosed syntaxins to the PAM “encasement”. It would therefore be interesting to test whether a similar differential behavior of SYP132α is also observed upon papilla formation or haustorial encasements of pathogenic microbes. Over-expression of VAMP721e together with SYP132α caused a shift in the accumulation of SYP132α toward the degrading parts of the arbuscule. Since non-symbiotic syntaxins appear to make more stable complexes with VAMP721e compared to SYP132α, it suggests that a faster dissociation of *cis-*SNARE complexes prevents the redirection of SYP132α to the degrading parts, ensuring a preferential localization at the functional arbuscule domains (Huisman et al., 2016). This supports a so-far understudied role for PM-SNARE complex formation in the regulation of endocytic traffic in plants. Interestingly, it has recently been shown that AtSYP132 (the ortholog of MtSYP131) affects the endocytic traffic of an H+-ATPase, with which it interacts (Xia et al., 2019). Induced expression of AtSYP132 reduced the amount of H+-ATPase proteins at the PM thereby affecting the acidification of the cell wall. This suggests a role for syntaxins in the selective turn-over of proteins at the PM. It is therefore tempting to speculate that MtSYP132α may control the endocytic turnover of specific proteins at the PAM to control arbuscule function, rather than morphology.

A speculative specialization of SYP132α, that can be independent from its role in vesicle fusion, may involve an interaction with PAM localized transporters. Such interaction could affect the functional efficiency of nutrient exchange, without affecting arbuscule morphology *per se*. Similarly, the t-SNARE AtSYP121 was shown to interact with K^+^-channels KC1 and KAT1. As a result of this interaction, K^+^ uptake is reduced in *Atsyp121* mutants (Grefen et al., 2010). Also in animal systems, syntaxins have been shown to interact with and directly regulate a range of ion channels or transporters (Michaelevski et al., 2003; Chao et al., 2011). Intriguingly, although syntaxins interact with the same v-SNAREs, it has been reported that they can mediate the secretion of distinct cargoes (Kalde et al., 2007; Leucci et al., 2007; Rehman et al., 2008; Waghmare et al., 2018). How different syntaxins control the delivery of different cargo, despite a lack in specificity for different v-SNAREs, will be an interesting future avenue to study how the exocytosis-machinery may be specialized for different biological functions.

## Materials and Methods

### Phylogenetic Analysis of SNAREs

The protein sequences of all SYP1 and VAMP72 family members were retrieved from the Phytozome database^1^ for the AM host species; *Aquilegia coerulea, Brachypodium distachyon, Citrus clementina, Carica papaya, Cucumis sativus, Fragaria vesca, Ginkgo biloba, Musa acuminate, Manihot esculenta, Medicago truncatula, Oryza sativa, Phoenix dactylifera, Prunus persica, Populus trichocarpa, Setaria italic, Solanum lycopersicum, Selagionella moelendorfii, Theobroma cacao* and *Vitis vinifera*, and the AM non-host species; *Arabidopsis thaliana, Beta vulgaris, Dianthus caryophyllus, Marchantia polymorpha, Nelumbo nucifera, Physcomitrella patens, Pinus taeda, Striga hermonthica, Spirodela polyrhiza*, and *Utricularia gibba* (Supplementary Datasheets S1, S2). To ensure the recovery of all family members, missing species in each orthogroup were confirmed by repeated homology searches using orthologs from that group as an input. The species were choses to include a large and diverse range of AM hosts and non-hosts. The Protein sequences were aligned in Mega5 using the ClustalW algorithm. Subsequently, trees were constructed using the neighbor-joining method with 100 bootstrap iterations.

### Plant Growth, Transient Transformation, and Inoculation

For transformation, *A. rhizogenes* MSU440 was used according to Limpens et al. (2004). For nodulation assays, plants were transferred to perlite saturated with Färhaeus medium without Ca(NO_3_)_2_ and grown at 21°C at a 16/8 h light/dark regime. After 3 days, plants were inoculated with Sinorhizobium melilotii 2011 and grown for 4 weeks. For mycorrhization assays, plants were transferred to pots containing a 5:3 (v/v) ratio mix of expanded clay and sand, saturated with modified Hoagland medium (5 mM MgSO_4_, 2.5 mM Ca(NO_3_)_2_, 2.5 mM KNO_3_, 2 mM NH_4_NO_3_, 500 μM MES, 50 μM NaFeEDTA, 20 μM KH_2_PO_4_, 12.5 μM HCl, 10 μM H_3_BO_3_, 2 μM MnCl_2_, 1 μM ZnSO_4_, 0.5 μM CuSO_4_, 0.2 μM Na_2_MoO_4_, 0.2 μM CoCl_2_, pH 6.1). Plants were inoculated with dried Rhizophagus irregularis infected maize roots obtained from Plant Health Cure^2^. Plants were grown for 4 weeks at 21°C at a 16/8 h light/dark regime.

### Laser Capture Microdissection and ddPCR

Roots of mycorrhized Medicago plants and uninfected control plants were harvested and fixed in Farmer’s fixative (75% ethanol, 25% acetic acid) substituted with 0.01% Chlorazol Black E to stain AM fungi, and vacuum infiltrated for 30 min on ice. Then, the roots were incubated in Farmer’s fixative for 16 h at 4°C on a spinning wheel. After fixation, the roots were dehydrated in an ethanol dehydration series (80, 85, 90, 95% 30 min each followed by 100%, overnight) Steedman wax was prepared by mixing 90% polyethylene glucol400 distearate and 10% 1-hexadecanol at 65°C. Steedman wax was infiltrated by incubating the roots in 50% Steedman wax and 50% ethanol for 2 h at 38°C, followed by three incubations in 100% Steedman wax for 2 h at 38°C. Finally, the samples were transferred to room temperature to allow the wax to solidify. Solidified blocks of Steedman wax were cut into 20 μm thick sections using a microtome, and transferred to PEN-membrane slides (Leica). Three replicates of Arbuscule containing cells and uninfected cortical cells were collected using a Leica LMD7000 laser capture microdissection microscope. RNA was isolated using a RNeasy micro kit (Qiagen). cDNA was synthesized using the iScript cDNA synthesis kit (Bio-Rad). in a total volume of 20 μl. 1 μl cDNA was then used per ddPCR reaction. For this, a ddPCR mastermix containing evaGreen as a probe was used (BioRad), as well gene specific primers (1–24, Supplementary Table S2). Then the PCR mix was suspended in oil using the QX200 Droplet Generator (Biorad). The PCR was carried out following manufacturer’s instructions. Subsequently, the absolute number of positive droplets was counted using a QX200 Droplet Reader.

### Whole Root RNA Isolation, cDNA Synthesis, qRT-PCR

RNA was isolated from plant tissue using the EZNA Plant RNA mini kit (omega). cDNA was synthesized from 1 μg of RNA using the iScript cDNA synthesis kit (BioRad). Equal amounts of cDNA were used for qPCR using SYBR green supermix (Bio-Rad) in a Bio-Rad CFX connect real-time system qPCR machine. Gene expression levels were determined using gene specific primers listed in Supplementary Table S2. The gene expression was normalized using Actin2 and Ubiquitin10 as reference genes. To confirm RNA silencing levels, five biological replicates were checked. Only plants in which silencing resulted in trancript levels below 20% of the empty vector control were considered in subsequent phenotypic analysis. These were 3–4 biological replicates per construct.

### Plasmid Construction

All expression cassettes were created using multisite gateway technology (Invitrogen) in the pKGW-RR-MGW destination vector. For all reactions a pENTR2-3 carrying a 35S terminator was used (Ovchinnikova et al., 2011).

The SYP132α RNAi construct, the empty RNAi control vector, a pENTR4-1 vector carrying a PT4 promoter, a pENTR4-1 carrying a PT4 promoter fused to GFP, The pENTR1-2 vectors containing the coding sequences of SYP121, SYP122, SYP132α, and SYP132β and a pENTR4-1 carrying the SYP132 promoter are described in Huisman et al. (2016).

The VAMP721d/e RNAi vector, the pENTR1-2 vectors containing the coding sequences of VAMP721a, VAMP721d and VAMP721e, and the pENTR4-1 carrying the VAMP721e promoter are described in Ivanov et al. (2012).

To obtain a pENTR4-1 containing a triple HA-tag driven by the PT4 promoter, A triple HA tag flanked by AscI and Acc65I restriction sites was *de novo* synthesized (Integrated DNA technologies). Using AscI-Acc65I restriction-ligation, The GFP in the pENTR4-1 pPT4::GFP was swapped for the triple HA-tag. A pENTR1-2 carrying a Timer-NLS construct was generated by amplifying the timer-NLS cds from a vector described in Mirabella et al. (2004), using primers 53 and 54 (Supplementary Table S2) adding a cacc sequences in the forward primer. The PCR fragment was then cloned into a pENTR/D-TOPO entry vector using TOPO cloning (Invitrogen). A pENTR4-1 carrying the fluorescent timer driven by the PT4 promoter was constructed by amplifying the timer cds using primers 55 and 56 (Supplementary Table S2), while adding AscI-KpnI restriction sites. By AscI-KpnI restriction digestion, the GFP of the pENTR4-1 pPT4-GFP was swapped for Timer. pENTR2-3 vectors containing nGFP or cGFP were obtained from VIB Ghent. For n-terminal split GFP fusions, nGFP and cGFP were amplified from these vectors using primers 41–44 listed in Supplementary Table S2. Primers were designed to remove stop codons, and to add a start codon and AscI/Acc65I restriction sites. Subsequently, both fragments were cloned into a pENTR4-1 vector containing a 35S promoter using AscI-Acc65I restriction/ligation. pENTR1-2 vectors containing the coding sequence of SYP131, VAMP724 and VAMP727 were constructed by amplifying the respective genes from Medicago A17 cDNA using primers 29–34, adding a cacc sequence adapter at the 5′ end. The PCR fragment was then cloned into a pENTR/D-TOPO entry vector using TOPO cloning (Invitrogen). To combine GFP fusion cassettes with 3HA fusion cassettes, the GFP fusion constructs were amplified using primers 35 and 36 (Supplementary Table S2), adding SpeI and SwaI restriction sites. The HA fusion constructs were amplified using primers 37 and 38 (Supplementary Table S2), adding SwaI and ApaI restriction sites. Using three-point restriction/ligation, the two constructs were inserted into a pKGW-MGW binary vector. To combine SNARE expression cassettes with RNAi constructs for RNAi complementation, the SNARE expression cassette was amplified using primers 39 and 40 (Supplementary Table S2), adding ApaI and Eco81I restriction sites. Using ApaI-Eco81I restriction/ligation, the cassetes were inserted into the SYP132α and VAMP721d/e RNAi vectors.

To make a CRISPR-Cas9 construct for SYP132α we made use of the pCAMBIA1302-Cas9 vector described by Jiang et al. (2013). A 20 bp region (GCAACTGATCATGTGAAGTC) targeting the last exon of SYP132α was chosen as sgRNA target sequence. Overlap PCR was performed to introduce the selected 20 bp target sequence into the U6-sgRNA cassette from pCAMBIA1302-Cas9, flanked by SalI and KpnI restriction sites, using the primers 45–48 (Supplementary Table S2). The resulting SYP132α-sgRNA fragment was introduced into the pCAMBIA-CAS9 vector via SalI-KpnI restriction-ligation. pCAMBIA-CAS9 additionally contains the CAS9 gene under the control of the CaMV35 promoter and a hygromycin resistance gene for selection in the plant.

### Microscopy and Quantification

For confocal imaging, a Leica SP8 confocal microscope was used. An excitation wavelength of 488 nm was used for GFP and WGA-alexa 488. An excitation wavelength of 543 nm was used for dsRed. Appropriate emission range settings were used to separate the fluorophores used in each experiment. For quantification of fluorescence levels, ImageJ software was used. The average arbuscule size was determined from 1000 arbuscules in each of the four roots per genotype.

### Mycorrhizal Staining and Quantification of Colonization Levels

For quantification of colonization levels, roots were incubated in 10% (w/v) KOH at 98°C for 10 min. Then roots were washed three times with 5% acetic acid. After washing, the roots were stained in 5% ink in 5% acetic acid, for 2 min at 98°C. after staining the roots were destained in 5% acetic acid, refreshing the destaining solution several times. For staining with WGA alexafluor 488, roots were incubated in 10% (w/v) KOH at 60°C for 3 h. Then, roots were washed three times in PBS (150 mM NaCl, 10 mM Na_2_HPO_4_, 1.8 mM KH_2_PO_4_, pH 7.4), and incubated in 0.2 μg/mL WGA-Alexafluor 488 (Molecular Probes) in PBS at room temperature for 16 h. For quantification of colonization levels, roots were cut into 1 cm fragments, and the colonization and arbuscule abundance was scored and calculated according to Trouvelot et al. (1986).

### Co-immunoprecipitation

The roots of mycorrhized plants (5 weeks post-inoculation) expressing different combinations of GFP-labeled syntaxins and 3HA-labeled VAMPs were harvested, and residual sand was washed away. The roots were flash-frozen in liquid nitrogen, and ground using a mortar and pestle. One gram of plant material was added to 6 ml of RIPA buffer [10 mM Tris/HCl pH 7.5, 150 mM NaCl, 0.1% SDS, 1% Triton X-100, 1% sodium deoxycholate, 0.5 mM EDTA, 1 mM PMSF (from a 100x stock in isopropanol), 20 μM MG132, 1x protease inhibitor mix (Roche cOmplete, EDTA free)], and ground in a potter tube. After 20 min incubation on ice, the extracts were centrifuged twice at 9000 *g* after which the supernatant was transferred to a new tube each time. The input fraction was harvested at this point, and mixed in a 1:1 ratio with 4x SDS-sample buffer (200 mM Tris HCl 6.8, 8% SDS, 40% glycerol, 4% β-mercaptoethanol, 50 mM EDTA, 0.08% bromophenol blue). Then 30 μl of anti-GFP coated agarose beads (Chromotek), equilibrated in washing buffer [50 mM Tris HCl 8.0, 150 mM NaCl, 0.1% Triton X-100, 1x protease inhibitor mix (Roche cOmplete, EDTA free)] were added to the protein extracts. The samples were incubated for 1 h at 4°C on a spinning wheel. Then, the beads were harvested by centrifugation at 2000 *g* for 2 min, and washed three times with 1 ml of washing buffer. The washing buffer was removed, and 100 μl 2x SDS-sample buffer was added, after which the samples were incubated for 10 min at 98°C. After centrifugation for 2 min at 2700 *g*, the supernatant was transferred to a new tube and stored at −20°C until gel electrophoresis.

### Western Blotting

Proteins were separated on a precast 4–12% poly acrylamide gradient mini-gel (Bio-Rad) at 300 V. Then the proteins were transferred to a PVDF membrane using the BioRad Trans-Blot turbo system. The blot was blocked for 1 h with 3% BSA in TBST (50 mM Tris HCl 7.4, 150 mM NaCl, 0.3% Tween 20) while shaking and washed three times with TBST. Antibodies against GFP (Miltenyi Biotec) or HA (Pierce scientific) conjugated to horse radish peroxidase diluted 5000x in TBST with 1% BSA were added, and incubated for 1 h while shaking. Then, the blot was washed three times with TBST, and one time with TBS (50 mM Tris HCl 7.4, 150 mM NaCl). Finally, 1 ml of supersignal west femto ECL substrate (Thermo scientific) was added to the blot, and luminescence was measured for 5 min, using a G:box detection system (Syngene).

### Stable Transformation of *Medicago truncatula*

The binary plasmid carrying SYP132α-sgRNA/35S::CAS9 construct the was introduce into *Agrobacterium tumefaciens* AGL1 via electroporation. Stable transformation of *Medicago truncatula* R108 cotyledon and young leaf explants was done according to Chabaud et al. (2003). Transformants were selected using 10 mg/L hygromycin B. DNA was extracted from the transformed lines using the standard CTAB miniprep method. The resulting lines were genotyped, and resulting PCR amplicons sequenced, using the primers 49 and 50 (Supplementary Table S2). The presence of the Cas9 gene in the obtained lines was checked by PCR using primers 51 and 52 (Supplementary Table S2).

### *Agrobacterium* Infiltration of *Nicotiana benthamiana*

*Agrobacterium tumefaciens* C58 expressing split-GFP constructs were grown in liquid LB with appropriate antibiotics for 2 days at 28°C. The bacteria were collected by centrifugation, resuspended in MMi medium [10 g/l sucrose, 5 g/l MS basal salts (Duchefa), 2 g/l MES, 200 μM acetosyringone, pH 5.6] to an OD_600_ of 0.1 and incubated for 1 h at room temperature. Different combinations of split-GFP constructs were made by mixing the appropriate bacterial suspensions in a 1:1 ratio. The suspensions were then injected into the leaves of *Nicotiana benthamiana* plants which were then grown in a greenhouse at 21°C. Three days post-infiltration, the infiltrated parts were analyzed by confocal microscopy.

## Data Availability Statement

All datasets generated for this study are included in the article/Supplementary Material.

## Author Contributions

RH, JH, and EL performed experiments and data analyses. RH and EL conceived experiments. RH, TB, and EL wrote the manuscript.

## Conflict of Interest

The authors declare that the research was conducted in the absence of any commercial or financial relationships that could be construed as a potential conflict of interest.

## Footnotes

1 http://www.phytozome.net

2 http://www.phc.eu
