# Supplementary material for: SNARE Complexity in Arbuscular Mycorrhizal Symbiosis
Source: Front Plant Sci. 2020 Apr 3;11:354. doi: 10.3389/fpls.2020.00354 (PMC7145992; doi:10.3389/fpls.2020.00354)
Supplement: Supplementary file 1 [file Data_Sheet_1.docx]

>SmSYP121_130326

MNDLLRSS

LRSSQSYLDLKRDVLRDLEVGPEMELGSLET

EKNLEHFFDEVEQINIGMEKIQQLLSKLQDANEESRGIYKALAMKAI

RDRMDKDVLEVLRLAKSIKDRLEELDKANIVNRKIPGCEQGTPADRTRM

SITMSMRMKLKDLMGDFQALRQKMMGEYRETVERRYFTVTGHQADE

ETIEKIIETGESEVFVQRAIQDQGRGQVLETIREIQERHDAV

KEIEKNLLELHQIFLDMAVLVEAQGEQLNDIERHMSMAANY

VDKGNSQLHYAKQHQRSSRKCTLIAIILLLVVLM

VVVIPVVTSFKSA*

>SmSYP122_151097

MNDLMSRS

FSGPRSYVDLRHDRDLEAGGETEMTVVDS

ERNLALFLEEANEIQAKMKSVEGLLARLQQANEESKSIHKAQAVKAI

RQRMDKDVEQVLKLAKSIKAQLEELDKANAANRKIAGFGAGTPTDRTRT

SLTASMRKKLKDIMGDFQELRQRMKGEYREAIGRRYFTVTGTDPDE

ETLETMIETGESETFLQRAIQEQGRGQVMETIREIQERHDAV

REIEKNLLELHQIFLDMAVLVEAQGEQLNSIEDQVHRASSF

VARGTTNLQVAKKHQRSARKWTCIGIIILIIVVI

VILVPILVRARNNSSSPSPSPNPVTPSPPPPVAPPPPT*

>SmSYP123_25349

LGRFFDEVGKIKSDMERLKMLLGKLQAAHEESKTIHKAKAMKAL

RQRMDKDIEEVLVKAKLIKDEIQKLDRSNIASRQVGGCEEGTPTDRTRT

VITANLKKNLQDVMAEFQKLRHKITGEYRDTLIRRFFTVTGKKPDD

ETVDYILETGESESFLQRAIQDQGRGQIVETIREIQERHDAV

KTMEKNLLELQQIFLDISVLVESQGEQLNSIEHQVHRAAAY

VEQGAGSLRGARKIQRSSRKCMCIAIVLLLVVIL

VVAAPV

>SmSYP123_74098

MNNLLSS

SFPRGSDMEASPLDVEMAVHN

SEKSLVGFFEEVSKIKDGMDAIQELLLKLNEDNESTKGIHRPGAMREL

RLRMDEHVSLVLKNGRSIKSKLEALDASNLANRKVKGCEEGSSTDRTRM

SITNSLRIKLRDLMADFANLRERMSSEYRGTIERRYFTVTGEQASQ

EVIERMIQTGESENFLRKAIQEQGRRQILDTIQEIQERHDAM

MEVEKNLRELQQIFQDLATLVDAQGAQLNTIEEHVNKAASF

VDRGTQQLVKAKRSQRRSRKWTCIGIIILIIILL

VILLPILTRR*

>SmSYP124_93442

MNNLLSS

SFPRGSDMEANPLDVEMAVHN

SEKSLVGFFEEVSKIKDAMDAIQELLLKLNEDNESSKSVHKPGAMREL

RSRMDEHVSLVLKNGRGIKSKLEALDASNLANRKVKGCEEGSSTDRTRM

SITNSLRIKLRDLMADLANLRERMSSEYRGTIERRYFTVTGEQASQ

EVIERMIQTGESENFLRKAIQEQGRGQILDTIQEIQERHDAV

VEVERNLRELQQIFQDLATLVDAQGAQLNTIEEHVNKAASF

VDRGTQQLVKAKRSQRRSRKWTCIGIILLIIILL

VILLPILTRK*

>BdSYP121_Bradi1g06372

MNNLFSS

SWKRAGDGDLESGGVEMSAPPGAA

AGASLDKFFEDVESIKDDLRDLDRIQRSLHDGNESGKSLHDASAVRAL

RARMDADAAAAIKKAKVVKLRLESLDRANAANRSVPGCGPGTSTDRTRT

SVVAGLRKKLRDSMESFSALRARVSSEYRDTVARRYFTVTGAQPDE

ATLDTLAETGEGERFLQRAIAEQGQGRGEVMGVVAEIQERHGAV

ADLERSLLELHQVFNDMAVLVAAQGEQLDDIEGHVGRARSF

VDRGREQLQVARKHQKSSRKWKFIAIGILLVIILV

IVIPIVLKNTKNSSSSNNSNQPQQ*

>BdSYP111_Bradi1g09440

MNDLMTK

SFMSYVDLKKAAMKDLEAGGGDETELTEAGCAAGGV

TDERLKGFFKEAEVVKEEMAAIRDALARLHAINEEGKSLHQPEALRAM

RGRVNADIVAVLRRARDIRVRLEAMDRANAAQRRLSAGCSEGTPLDRTRT

SVTAGLRKKLKDLMLDFQALRQRMMSEYKETVERRYYTLTGEVPED

EVIERIISEGRGEEIMGAAVAEHGKGAVLAALHEIQDRHDAA

REVERSLLELHQVFLDMAVVVESQGEQIDDIERHVVNARDY

VHTGNKELGKAREHQRSSRKCLCIGLILLLLLIL

IVVVPVVTSFKTS*

>BdSYP125_Bradi1g37150

MNDLFSTS

SFKKYADASPQGAGGGDMEAGGESVA

NLDMFFEEVEAVKEDMRGFETLYKRLQSTNEETKTAHEARAIKSL

RSRMDGDVEQVLKRAKAVKAKLEALDKDNANSRKAPGCGPGSSTDRTRT

SVVAGLGKKLKDIMDDFQGLRTRMAAEYKETVARRYYTVTGEHAEE

STIESLISSGESESFMQKAIQDQGRGQVMDTISEIQERHDAV

KEIERSLMDLHQVFLDMAALVEAQGHQLNDIESHVAHASSF

VRRGTVELETAHEIQKDSRKWMCFAVLGGIAIVIVL

VTPVLINLHILTLR*

>BdSYP124_Bradi3g07970

MNDLFSSG

SFKKYEDLKYQVALDDMESGLEVAAA

NLDKFFEDVEAVKEDMRGLEALHRRLQSGNEEVKAAHDAGAVRSL

RARMDADVAQVLVRAKAVKAKLESLDRDNAASRATLPGCGPGSSTDRTRS

SVVSGLGNKLKDLMDDFQGLRTRMAAEYRETVARRYFTVTGEQAEE

SRIEALIASGESETFLQKAIQQQGDQQAAGRGQVMGTVSEIQERHDAV

REMERSLRDLHALFLDMAALVEAQGHQLNDIQSHVAKASSF

VHRGAVELESARGYQRSSRKWGCVGLVAAVVLLLVV

LLPILVNLKLLTVG*

>CcSYP121_Ciclev10012164m.g

MNDLFSG

SFSRFRSEQASPDHPHHHTTIQMTENPNST

GGVNLDKFFDDVESVKDELKELERLHNSLQTSHEKSKTLHNAKAVKDL

RSRMDADVSAALKNAKLIKIRLEALDRSNAANRSLPGCGPGSSSDRTRT

SVVNGLRKKLKDSMESFNELRQRISSEYRDTVQRRYYTVTGENPDE

KTLDRLISTGESETFLQKAIQEQGRGRILDTINEIQERHDAV

KVLEKNLQELHQVFLDMAVLVQAQGEQLDDIESQVQRANSY

VRGGTERLQTARKYQLSTRKWTCIAITILLIIILF

VVLFTVKPWDNGGGNGGGGSSPQNPTPQAQTPPS*

>CcSYP125_Ciclev10016060m.g

MNDLFSN

SFKKYTDLKTQAYQDDMEAGRE

RDNLDKFFEDVENVKAEMKTVEKLYKRLQEANEESKIVHNARTMKEL

RARMDADVQQVLKRVKIIKGKLEALERSNAASRNIPGCGPGSSSDRTRT

SVVSGLGKKLKDLMDDFQNFRNKMQYEYKETVERRYFTVTGQKADE

ETIENLIASGESESFLQKAIQEQGRGQILDTISEIQERHDAI

KEIEKNLLELHQVFLDMAALVEAQGHQLNDIESHVAHANSF

VRRGTEQLQEARELQKGSRKWTCYAIIAVIILIIVI

LLPFFPHIMMIIREH*

>CcSYP124_Ciclev10017913m.g

MNDLISN

SFKRYSNVDLEAGGEIRKG

TENLDKFFDDVENVKEDMKVVEKLYKRLQESNEETKVAHNAKTMKQI

RARMDSDVEQVLKRVKVIKGKLEALEKSNAANRKVPGCGPGSSVDRTRT

SVVAGLGKKLKDTMDDFQHLRAKMTAEYQETIERRYFTVTGQQADE

QMIENLISSGESENFLQKAIQEQGRGQIMDTISEIQERHDAV

KEIEKNLLELHQVFLDMFALVEAQGHQLNDIESHVAHANSF

VRRGTEQLEFARESQKDSRKWTCIAILLGIILIIVF

LFPLISTLIVNSPQ*

>CcSYP111_Ciclev10021255m.g

MNDLMTK

SFTSYVDLKKEAMKDLEAGPDPDLEMTTSTNT

MDQNLNLFLAEAENVKKEMEEIRDVLGRLQEANEQSKSLHKPEALKLL

RHKINKDVVTVLKRARNIKSHLEDMDRANAANKRLSGCKEGTPIYRTRI

AVTNGLRKKLKELMMEFQGLRQKMMTEYKETVGRRYFTVTGEYPNE

DVIEKIISDNNGSEEFLTHAIQEHGRGKVLETVVEIQDRHDTA

KEIEKSLLELHQIFLDMAVMVEAQGEQMDDIEHHVMNASHY

VKDGTKELKTAKDYQRSNRKWMCVGIILLLLIIL

LIVIPIATSFSSS*

>CcSYP112_Ciclev10027293m.g

MNDLMTK

SFLSYVELKKQAQKDLEADLDIERGQLNPR

DEENLTQFFQEVDAIKDEMEEITNLLLDLQNLNEGTKSTHSGKVLRGL

RDRMESDVASILRKAKIVKARLESLDKSNMINRTLSQAFKEGSSVDRTRI

SVTNGLRVKLRDLMNDFQSLRGKVLSDYKEDLKRRYYNATGEEPSE

DVIEKVISGSGKVEILEGKTEKDIQRSKERHEAV

MDIQRSLTKLHQVFLDMAVLVETQEEKMDDIEENVANAGNF

ISGGTNSLYYANQTRKKRSWVCSVWAVGLIILLVCLIS

LLTSR*

>CsSYP111_Cucsa.024000

MNDLMTK

SFTSYVDLKKAAMKDLDLEAGLEKASSVTG

DNGDMGLFLEEAEKVKTEMGSIREILVKLQQANEETKSAHKPETLKLL

RNAINVDIVTVLKKARSIRSQLEEMDRANAAKKRLSGSKEGTAIYRTRI

AVTNGLRKKLKELMMEFQSLRQRMMTEYKETVGRRYFTVTGEHPEE

EVIEKIISNGGEEFLARAIEEHGRGKVAETVVEIQDRHGAA

KEIEKSLLELHQVFLDMAVMVEAQGEKMDDIEHHVMNASQY

VIDGTKDLKTAKDLQRNSRKCLCFGILLLLVIIL

VVVIPIAVSFGSS*

>CsSYP124_Cucsa.126310

MNDLFSN

SFKKYTDLKQQAYLDSMEAGSE

SVNLDRFFEDVENVKDDMKQVENLYKKLQQANEECKVVHNAKTMKEL

RGRMETDVAQVLKRVKLIKGKLEALERSNAAHRGLPGCGPGSSADRTRT

SVVSGLGKKLKDVMDDFQGLRARMNAEYKETVERRYFTVTGQKANE

ETIENLISSGESESFLQKAIQEQGRGQIMDTISEIQERHDAV

KEIEKNLIELHQIFLDMAALVEAQGHQLNDIESHVAHANSF

VRRGTEQLQEAREYQKSSRKWTCYAILLGAILIIIL

LFPLLTSILPHLL*

>CsSYP112_Cucsa.127530

MNDLMTK

SFLSYVELKKQAQREAVGGGGHGFDIESGGQKLNPT

EEQNLSLFFEKVDEIKTQMEETTNLLVDIQKLNQEAKSTHNAKILRGL

RDRIDSDMVSTLRRARILKEKLASLDQSNTANRLISVAYGEGTIVDRTRT

SITNGLRVKLREMMNEFQGLREKVVADHKEDLRRRYFSATGEQPSE

EQVEKIMSGSLKLEMLGGKLSETESEDRVRHESV

MDIQRSLNKLHQVFLDMAILVESEGEKIEDIEENVAKAGQF

INGGTRSLYYAKQMKRKNKKWVYWVWAIIFVILLVCIVS

MLVC*

>CsSYP121_Cucsa.241950

MNDLFSSR

SFSRDARVVEMGNNASSSP

TAVNLDKFFEDVESVKDELKELERLYSNLHDSHEQSKTLHNAKAVKDL

RSRMDTDVSLALKKAKLIKVRLEALDRSNAANRSLPGCGPGSSSDRTRT

SVVNGLRKKLQDSMESFNNLRQQISSEYRETVQRRYYTVTGENPDE

KTIDVLISTGESETFLQKAIQEQGRGRILDTISEIQERHDAV

KDLERNLKELHQVFMDMAVLVHEQGEKLDDIESQVNRAHSF

VRGGTQELTTARVYQKNTRKWTIIAIIILLLVVL

VIVLSLQPWKKNNSSSPATP*

>CsSYP122_Cucsa.309900

MNDLFSTD

SFRQEHHQYRHHDTVEMSDNLPSS

TTINLNTFFDDVESVKAELTELEGLHRSLQNSHEQSKTLHNSKAIKDV

RSRMETAVTLALKKARFIKVRLEELDRSNEENRKLPGCGYGSSADRSRT

SVVSGLRKKLCDSMESFNRLREEITKTYKETIERRYFTITGENPDE

KTVELLISTGESETFLQKAIQKQGRGRVLETIQEIQERHDAV

KDIERNLRELHQVFLDMAVMVQAQGQQLDDIESQVTRANS

ALQTARYYQKNTRKWICIGVSVGATVILI

IIIVAIARAIKKKD?

>VvSYP125_GSVIVG01006716001

MNDLFSS

SFKKYTDLKQQTYMDDMESGKE

AVNLDKFFEDVENVKQDMGGVEKLYKQLQDANEESKTVHNAKTMKDL

RARMDSDVTQVLKRVKMIKGKLEGLERSNAASRNVPGCGPGSSADRTRS

SVVSGLGKKLKDMMDDFQGLRAKMSTEYKETVERRYYTITGQKADE

DTIENLISSGESESFLQKAIQEQGRGQIMDTISEIQERHDAV

KEIEKNLIELHQVFLDMAALVEAQGQQLNDIESHVAHASSF

VRRGTEQLQVAREYQKGSRKWTCIAIILGAIVVGLL

LLPILPTLISIKPPNLSVNE*

>VvSYP112_GSVIVG01018937001

MNDLMTK

SFLSYVELKKQAEMDLEAEAEADIEIGKLKPK

DEENLSQFFEEVAAIKTVMEEITNLVLDLHNLNEETKSTHSAEVLP

YREGSPVDRTRM

SVTNGLRSKLRDMMNDFHSLRERILWDHRETLKRRYYNATGSEASE

EVVEKMMTGSVQIEAFEGKTGGDLVNRERNEAL

REIQRSLDKLRQVLLDMAVLVGSQGEKMDDIEENVAIAGNF

ISGGTNSLVYAKQMKKGKKWVYWVWAVGLIILLVCFIS

MLTS*

>VvSYP124_GSVIVG01030578001

MNDLFSS

SFKRFTDLKPQSFDVEAGGDAGRE

SVNLEKFFEEVENVKDDMRAVENFYKKLQDLNEESKTVHNAKTMKDL

RARMDTDVVQVLKRVKIIKGKLEALERSNAANRNHPGCGPGSSADRTRT

SVVGGLGKKLKDMMDDFQNLRVRMNAEYKDTIERRYFTITGEKADE

ETIENLISSGESETFLQKAIQDQGRGQIMDTISELQERHGAV

KEIEKNLIELHQVFLDMAALVEAQGQHLNDIESHVAHASSF

VRKGTDQLQIARNYQKSSRKWTCIAVGLAICLIIVI

LFPVLKSLDMIHL*

>VvSYP111_GSVIVG01035559001

MNDLMTK

SFISYVDLKKEAMKDLEAGPEYDLQMSGTQ

MDRNLGLFLEEAEKVKQEMGLIREILGRLHEANEESK

AIKSQLEEMDRANAANMRLSGYKEGTPVYRTRA

AVTNGLRKKLKELMMDFQGLRQRMMTEYKETVGRRYFTVTGEYPDE

EVIEKIISNGEGGEEFLGRAIQEHGRGKVLETVVEIQDRHDAA

KEIEKKSQDCQRLPEEQQEVHVF

GCYTSADTNSYSG

HPHCYQF*

>OsSYP124_Os02g11890

MNDLFSSG

SFKKYADLKNQAALDDMESGGGGGGGGEGA

NLEQFFEEVEGVKGEMRGLEALHGRLQASHEGSKTAHDARAVRSL

RARMDADVEQVLRRARAVKGRLQALDRANAASRKLPGRGPGSSTDRTRS

SIVSGLGTKLKDLMDDFQGLRSRMAEEYKETVARRYYTVTGEKAEE

STVEALISSGESETFLQKAIQEQGRGQVLDTISEIQERHDAV

KEIERGLLDLHQVFLDMAALVEAQGHQLNDIESHVARANSF

VRRGAVELETAREYQRSSRKWACIAILAGVVLVVII

VLPIIVNLHLLTIR*

>OsSYP111_Os03g52650

MNDLMTK

SFMSYVDLKKAAMKDLEAGGDGVELPEVGV

TDERLKGFFQETEAVEEEMAAIRDALARLNAANEEGKSLHQPDALRAL

RGRVNADIIAVLRRARDIRARLEAMDRANAAQRRLSAGCREGTPLDRTRT

ALTAALRKKLKDLMLDFQALRQRIMSEYKDTVERRYYTLTGEVPEE

EVIERIISEGRSEELLCAAVAEHGKGAVLATVHEIQDRHDAA

REVERSLLELHQVFLDMAVVVESQGEQLDDIERHVNSATTY

VQGGNKELRKAREHQRSSRKWLCIGIIILLLLVL

LVIVPIATSFKRS*

>OsSYP121_Os03g57310

MNNLFSS

SWKRTGGGGGGDGDIESGGGVEMAPPPGAA

AGASLDRFFEDVESIKDELRDLERIQRSLHDANEGGKSLHDAAAVRAL

RARMDADVAAAIKKAKVVKLRLESLDRANAANRSVPGCGPGSSTDRTRT

SVVAGLRKKLRDSMESFSSLRARISSEYRETVARRYYTVTGEQPDE

ATLDNLAETGEGERFLQRAIAEQGRGEVLGVVAEIQERHGAV

AELERSLLELHQVFNDMAVLVAAQGEQLDDIETHVGRARSF

VDRGREQLVVARKHQKSTRKWTCIAIIILLVLILV

VVLPIVLKFVNNNKSSSSSPAPATPSPPPPTA*

>OsSYP125_Os06g39050

MNDLFSSS

SFKKYADASPASGVGGSDMEAGGEGVV

NLDRFFEDVEGVKEDMKGLEALYKRLQSTNEETKTAHDARAVKAL

RSRMDGDVEQVLRRAKAVKGKLEALDRDNATSRKVPGCGPGSSTDRTRT

SVVAGLGKKLKDIMDDFQGLRTRMAAEYKETVARRYYTVTGEKAED

STIDSLIESGESESFLQKAIQEQGRGQVMDTISEIQERHDAV

KDIERSLLDLHQVFLDMAALVEAQGHQLNDIESHVAHASSF

VRRGTVELEVAREHQKSSRKWACVAVLAGIILIAVL

ILPVLINLRILTLR*

>MeSYP121_08G034400

MNDLFSG

SFSRSRSGEASPDHHVIQMSETPPSS

DGVNLDKFFKDVDSVKDELKELEKLNENLQSAHEQSKTLHNAKAVKDL

RSRMDTDVAQALKKAKLIKVRLEALDSSNATNRSLPGCGPESSSDRTRT

SIVNGLRKKLKDSMESFNRLREKISSEYRETVQRRYFTVTGENPDE

KTLDRLISTGESETFLQKAIQEQGRGRILDTINEIQERHGAV

KEMEKNLKELHQVFLDMAVLVEAQGEQLDDIESNMQRASSF

VRGGTQQLQTARAYQKNTRKWTCYAIILLLIIIL

VVLFTVRPWE*

>MeSYP111_08G147400

MNDLMTK

SFTSYVDLKKEAMKDLEAGPDADLEMQNATNT

MDRNLSLFLEEAETAKKEMESIREILVRLQESNEESKSLHKPESLKSL

RNKINTDIVTVLKKARAIKSQLEEMDRANAANKRLSGYKEGTPIYRTRL

AVTNGLRKKLKDLMMDFQGLRQKMMTEHKETVERRYFTVTGEYPDE

EIIDKIISDENGGEEFLKRAVQEHGKGRVLETVVEIQDRHDAA

KEIEKSLLELHQVFLDMAVMVEAQGEQLDDIEHHVLNASHY

VKDGAKELKSAKDYQRSSRKWMCIGIILLLLIIL

VIVIPVATSFSHS*

>MeSYP122_09G044600

MNDLFSG

SFSRFRSEEASPDHHVIQMSEMPHTG

GVNLDKFFGDVESVKDELKDLERLNESLQSAHEQSKTLHNAKAVKDL

RSKMDADVAQALKKAKLIKVRLEALDRSNAANRSLPGCGPGSSSDRTRT

SVVNGLRKKLKDSMDSFNSLREKISGEYRETVQRRYFTVTGENPDE

RTLDRLISTGESETFLQKAIQEQGRGRILDTINEIQERHDAV

KDMEKNLKELHQVFLDMAVLVQAQGEQLDDIESNMQRASSF

VRGGTQQLNTARVYQKNTRKWTCYAIIILLVIILF

VVLFTVRPWENNGGGGGGGSQPAPAQTPPSPPPPPQ*

>MeSYP112_09G144500

MNDLMTK

SFTSYVDLKKEAMKDLEAGPDPDIEMANSSNT

MDRNLGLFLEEAENVKKEMGSIRDILVRLQESNEEIKSQHKPEALKSL

RNKINIDIVTVLKKAKAIKTQLEEMDRANAANRRLSGFKEGTPIYRTRL

AVTNGLRKKLKELMMDFQGLRQKMMIEYKETVERRYFTVTGEYPDE

EIIDKIISDDNGGEEFLKRAIQEHGKGKVLETVVEIQDRHDAA

KEIEKSLLELHQVFLDMAVMVEAQGEQLDEIEHHVFNASHY

VKDGTKELKSAKDYQRSSRKWMCIGVILLLLIIL

VIIIPVATSFSDS*

>MeSYP113_11G086000

MNDLMTR

SFLNYVELKKEGIKDLESEPDLEMGQLDTA

DEQNLSKFFEEVNEIKIVMEEIANLLHDLQGLNEDSKSTHSMKVLKGI

RDRINSDMVAILRKAKIIKSRLESLDRSNMDNRNLSVAYKEGSPIDRTRV

SVTNGLRINLRNMMHDFQSLRAQILKDHKEGFKRRYYNATGEQLSD

EMLDKMVFEGGQEKVFEGKADLVMENQERHEAL

KEIQRSLTELHQLFLDMAVLVEKQGDEINNIEENVACAGTY

INGGTNGLYYAKQMKKNRRNWSCWIGLLLLVLLVILVST

LAS*

>MeSYP124_16G021400

MNDLFSN

SFKRFSDVKEQPNLDDLEAGKGN

MSLDKFFDDVENVKEDMKEVEKLHKKLQETNEQSKTAHNAKTMKNL

RARMDSDVEQVLKRVKLIKGKLEALERSNAAARNNPGCGPGSSADRTRT

SVVSGLGKKLKDLMDNFQNFRAQMSAEYKETVERRYFTITGEKASE

ETIENLISSGESESFLQKAIQDQGRGQILDTISEIQERSDAV

KEIEKNLIELHQLFLDMAALVEAQGHQLNDIESHVAHASSF

VRRGTEQLVEAREYQKSSRKWTCIAIYGFIILLFLL

LLPLLPTIIALM*

>Mp_Mapoly0009s0005

MNDLLGES

GRHSTSAQDVTGNGREKNGKDNNDLEAGPSGTPD

GGADMLQFFNEVGVIKTDMAQIRKNLAKLQDAHEETKTVTNAKAMKAL

KERMEKDIDEVSKVAQHIKGKIEALDKSNIANRKKPNCGEGSSTDRTRM

SMTATLKKKLKELMTEFQALRQKFTDEYREVVERRVFTVTGQKADE

GTIDQLIETGDSEQIFQKAIQEQGRGQILDTIAEIQERHDAV

KDIEKKLLELHQIFLDMAVLVEAQGELLDNIETQVSKAVTY

VQEGTVALQTAKKLQRGTRKCMCIAIILLLIIII

IIVVAVVQPWKTNKA*

>Mp_Mapoly0055s0091

MNDLLGDS

SREFSAAGGGTTAGSVEMSTNGKKKKRDVEAGREENEAE

GDPGMDEFFREEAAIQGDLSQITSLLKKLEEAHQESQTLTNAKALKAL

KERMARDLDDVSKVVRHIKGKIEALDRSNLASRKKPNCGEGTSTDRTRM

GLTAGLKVKLKDLMTQFVNLRQSFNDEYRQVVERRVFTVTGQKADE

EMIDQLIETGNSEQIFQKAIQAQGRGQILDTIAEIQERHDSV

KDIEKKLLELHQIFLDMAVLVEAQGELLDNISKNVSTAQDY

VARGGVALGQARKLQKGTRKCMCYAVILLLIIIL

IIVLATVKPWQK*

>Mp_Mapoly0101s0013

MNDLLAR

VFSRRDVVPDDVERGPAAFAMEEGTSSE

AGDELSGFFQEVDTLKKEMARVRDLLTKLQDAHEESKTARHTPALQEL

RDRINEDIGEVTKTSRLIKQGLEDLNKSNAASREIKGCEKGSSTDRTRI

QLTNSLTESLKDLMHDFGTLRTRIVGEYREIIGRSYYTVTGQRADE

TTIDRMVESGESETFIQRAIQEQGKGEVIDSLRDIQEQHEAV

KDIERNLQELNQIFVDLSVLVEAQGALVNDIQTNVERADSY

TKRAAVHLQVAKTHQRKNRLWMCAGVSVMLIAAG

VVAAIIATAK*

>Mp_Mapoly0163s0015

MNDLLQKT

YSGGRGYLNMRDVEKLGGEMEMQGIDP

EKNLASFFDEVNVIKTDMERIKSLLAKLQDSNEESKTIHKVQAMKAL

RDRMDKDLAEVSKVARSVKQKLEELDKANAASRRTKGCEEGTPTDRTRS

SITNSLTKKLKDLMESFGTLRSKIMVEYRETIERRYYTVTGQKPDE

ETLEQIIDTGESENFLQKAIQEQGRGQIIETIKEIQERHDGV

KEIEKSLLELHQIFLDLAVLVESQGTVLDNIESQVNRAHSY

VEKAGAHLTVAKKHQRNTRKWTCIAIIIVLIIIL

VIVVPIATSLKK*

>FvSYP111_gene00273

MNDLMTK

SFTNYVDLKKEAMKDLDLEAGNLELSTN

MHSDMGLFLEEAEKVKQEMACVRDILGRLQQANEESKSLHKSEALQSL

RNRINADILSVLKQARTIRCHLEDMDRANAANKRLSGAKEGTPIYRTRM

AVTNGLRKKLKELMMEFQGLRQRMMSEYKETVGRRYFTVTGEKADE

EVIEKIISNGGEEFMARAVQEHGRGKVLETVVEIQDRHDAA

KDIEKSLLELHQVFLDMAVMVEAQGEQMDDIEHHVINASQY

VKDGTKQLNTAKGYQRSTRKWMCIGLIILLILIL

LIVIPIATSFAHS*

>FvSYP131_gene10390

MNDLLTD

SFEIPRGQPPRNGDIELGTGSTDITMNS

GELGLENFFKKVQEIEKQNEKLDKLLRKLQDAHEETKTVTKAPSMKAI

KKRMEKDVDEVGKIARFIKSKIEELDRENLANRQKPGCGKGTGVDRSRT

ATTLALKKKLKNKMSEFQTLREIIHQEYREVVERRVFTVTGTRADE

ETIERLIETGDSEQIFQKAIHEQGRGQITDTLAEIQERHDAV

RDLEKKLLDLQQIFLDMAVLVDAQGELLDNIETQVSSAVDH

VQQGNTALHKAKKLQKNSRKWMCIAILILLIIVV

IIVVAVLKPWSSNNELRFKSCDPFTFRVEWKILGCTVATN

SSTFSMSEQEASSFSINSYQAEQLLIGISEAELEAIVPDYFDAGFPTSNHSKVTAGCMFH

TLSDS*

>FvSYP112_gene14738

MNDLMTK

SFLSYVDLKKQAQMDMEAELDVEAGLGGQLNPK

DQENLSQFFHEVSAIKADMEEITLLLFDLQSLNEEAKSTHSSKILRGL

RDRMESDMSAVLRKARVVKARLESLDQSNVISRKMSVDYREGSTVDRTRM

SITAGLRVKLRDLMCDFQSLREKILSDHKEDLKRRYYSAVGEMPTE

EELEKMISGSGKIAMFEGKSELEIENKVRHEAV

MDIQRSLTKLHQVFLDMAVLVEAQGEQIDNIEENVAIGGQY

ISGGTNSLHYAKQMKKKNNKWVLWLCAVVVIIFLVCLIS

MLAS*

>FvSYP122_gene18300

MNDLLSN

SFSQASPDHHVIEMTSFD

ASSDLKKFFEDVDLIQTELNDLQKLNTSLKSAHEQSKSLHKAKEVKDL

RTRMDADVNLALKAVKLLKVRLETLDRSNTSARSLPGCGPGSSSDRTRT

SVVSGLRKKLKDSMESFNDLRQKISSEYKETIQRRYFTVTGENPDD

KTIDLLISTGESETFLQKAIQQQGRGQVLDTIYEIQERHNGV

KAMERNLNELHQVFLDMAVLVQAQGEQIDNIESHVDRASSF

VQHGNQQLQKARFLQKNTRKWTCYGILILLIIIGI

ILASTLT*

>FvSYP121_gene18301

MNDLFSG

SFSRFRSEPDNNDEHVIEMSSATGTG

TGVNLDKFFGDVESVKDELKELERIHQNLQAAHEQSKTLHNANTVKDL

RSRMDGDVTLALKKAKVIKVRLEALDRSNAANRSQPGCGPGSSSDRTRI

SVVNGLRKKLKDSMESFNSLRQKISLEYRETVQRRYYTVSGENPDE

KTLDLLISTGQSETFLQKAIQEQGRGQVLDTINEIQERHDAV

KDMEKNLHELHQVFLDMAVLVQAQGEQLDDIESHVQRASSF

VRGGTQELGKAKVYQRNTRKWTCYLIILLLVIAL

IIILSLKPWN*

>FvSYP132b_gene23921

MNDLLTD

SFVSEARAQPSGTNDIEMGIPRSMS

DAGMDSFNKQIQEVEKQVEKLSGLLKKLKDANEESKSVTKASAMKAI

KKRMEKDIDEVGKIARGVKGKLEGISKDNLANRQKPGCEKGTGVDRSRM

NMTNSLTKKFREIMIEFQTLRQRIQDEYREVVERRVITVTGTRPDE

ETIDNLIETGNSEQIFQKAVQEQGRGQVLNTLEEIQERHDTV

KEIEKKLLDLHQIYLDMAVLVDAQGEVLDNIETQVSNTVDY

MQSGTTQLQNAKKLQKNSRKWTCIAIMILLIIVA

VVVVGVLKPWKSS*

>FvSYP132a_gene23921

MNDLLTD

SFVSEARAQPSGTNDIEMGIPRSMS

DAGMDSFNKQIQEVEKQVEKLSGLLKKLKDANEESKSVTKASAMKAI

KKRMEKDIDEVGKIARGVKGKLEGISKDNLANRQKPGCEKGTGVDRSRM

NMTNSLTKKFREIMIEFQTLRQRIQDEYREVVERRVITVTGTRPDE

ETIDNLIETGNSEQIFQKAVQEQGRGQVLNTLEEIQERHDTV

KEIEKKLLDLHQIYLDMAVLVDAQGEVLDNIETQVTNAVDH

VQSGTDALTTAKSLQKKSRKCMIISIILLLIIAL

IIVLSILKPWKK

>FvSYP113_gene25264

MNDLMTK

SFTSYVDLKKQAMKDIEAEPDLEMGQLEPA

EEQNLANFFEEVTAIKADMEEISNLLLDLQDLNEETKSTHSAKVLRGL

RDRINADMVSILRKAKNIKARLESLDQSNIANREVAGYKEGSPVDRMRI

SVTSGLRVKLRDMMNDFLALREQIVKEHKEGLKRRYYNATGEEATE

EVIEKILRSGEVKVFEGKTELVMENQERDEAL

KDLKRSLTELHQVFLDMAVLVEAQGEQINDIEKNVADAAVY

ISGGTKELLHAKQLKKKRGKWVCWIMAFVLFVLLVCFIS

IIAA*

>FvSYP124_gene30689

MNDLFSS

SFKKYTDLKQQAYLDDMESGKE

NINLDKFFQDVENVKDDMKQVEKLYKQLQDANEECKIVHNAKTMKEL

RTRMDLDVEQVLKKVKIIKGKLEGLERSNAAHRNNPGCGPGSSADRTRT

SVVSGLGKKLKDMMDDFSGLRGRMTAEYKETVERRYFTITGEKASE

ETIENLISSGESENFMQKAIQEQGRGQIMDTISEIQERHDAV

KEIEKNLIELHQIFLDMAALVEAQGHQLNDIESHVMHASSF

VRRGTDQLQEAKEQQKKSRKWTCIAILLGIVLLIVI

LIPVLLQVFPHLM*

>PtSYP124_Potri.004G035400

MNDLFSS

SFKKYTDLKQQAQMDDMEAGKE

SMNLDRFFEDVENVKEDMKTVERLYKSLQEANEECKTVHNAKTMKNL

RSRMDIDVEQVLKRVKIIKGKLEALDRSNAAHRNIPGCGPGSSADRTRT

SVVSGLGKKLKDLMDNFQDLRARMAAEYKETVERRYFTITGERASE

ETIENLISSGESESFMQKAIQEQGRGQILDTISEIQERHDAV

KEIEKNLIELHQVFLDMAALVEAQGHQINDIESHVAHASSF

VRRGTEQLSEAREYQKSSRKWTCIAIVAGAVLIIVL

LLPFIPHLLALL*

>PtSYP112_Potri.005G121300

MTR

SFLSYVELKKQAQKDLQVELDIESG

QPNLSQFFHEVNGIKTEMEDITNLLFDLQNLNEESKSTHSAKVLRGL

RDRMESDIVAVLRKAMIVKARLESLDRSNISNRRVSELYKEGSPIDRTRI

SVTNGSRVKLREIMNEFHILRQKILSDYKNDLKRRYYTATGEEPSE

EEIENMISGGGGVQMFEGKGVMDLKNKERHEVV

MEIQRSLKRLHQMFLDMVVLIETQGEKMDDIEENVTNAGNF

LSGGTNSLYHANQMKKKRKTWFLWVFAVMLIIIFVCIIS

ETAWKGII*

>PtSYP121_Potri.006G202200

MNDLFSG

SFSRFHSEEASPDHHVIQMSEAQSTG

GGVNLDKFFEDVESIKDELKELERLNENMRSSHEQSKTLHNARAVKDM

RSKMDADVALALKRAKLIKVRLEALDRSNAANRSLPGCGPGSSSDRTRT

SVVSGLRKKLKDLMESFNGLRQKITTEYRETVERRYFTVTGENPDE

KTLDLLISTGESETFLQKAIQQQGRGRILDTINEIQERHDAV

KDLENNLKELHQVFMDMAVLVEHQGEQLDDIESHVQRANSY

VRGGTQQLQTARKLQRNSRKWTCYAIIILLIIIL

VVLLSVRPWEKKK*

>PtSYP113_Potri.007G023100

MNDLMTR

SFLSYVELKKQSQKDLKAELDIESGQLNPT

DEPNLSQFFREVNEIKIEMEEITNLLFNLQTLNEESKSTHSAKVLRGV

RDRMESDIAAVLRKAKIVKARLESLDRSNISNCKVSELYREGSPVDRTRI

SVTNGLRVKLREIMNEFQILREKIFSDYKDDLKRRYYTAAGEEPSA

EVIEKIISGGGGVQMFEGKGVMDLKSKEKHEAV

MDIQRSLKRLHQVFLDMAVLIETQGEKMDDIEENVAKASNF

VSGGTNSLYYANQMKKKRKTWCLWVLAVVVIIILVCIIS

TLAT*

>PtSYP114_Potri.009G117900

MNDLMTK

SFLDYVDLKKQAIENIQPEPDLEMGKLDST

DERNLSKFFEEVKAIKIDMEEITNLLIDLQDLKEESRSTHSAKFLKGI

RDRINSDMVTILRKAKKIKSRLESLDQSNVANRRLSKAYKEGSSVDRTRV

SVTNGLRVKLRDMMHDFQALRENILKDHKEGLKRRYYNATGEHPTE

EMIERMILRGEKERVFEGKAELVMENLERHEAL

KKIQRSLTELHQVFLDMAILVEIQGDEINVIEENVAGAANH

ISGGTNGLYYADQMKRRGSHWACCG*

>PtSYP125_Potri.011G043700

MNDLFSS

SFKKYTDLKQQAQIDDMEAGKE

GMNLDRFFEDVENIKEDMKTVERLYRSLQEANEESKTVHNAKTMKNL

RSRMDMDVEQVLKRVKIIKGKLEALDRSNAAHRNIPGCGPGSSTDRTRT

SVVSGLGKKLKDLMDNFQDLRARMAAEYKETVERRYFTITGERASD

ETIENLISSGESESFMQKAIQEQGRGQILDTISEIQERHDAV

KEIEKNLIELHQVFLDMAALVEAQGHQLNDIESHVAHASSF

VRRGTEQLQEAREHQKSSRKWTCIAIIAGVVLIVVM

LLPFLPQILALL*

>PtSYP111_Potri.013G053200

MNDLMTK

SFMSYVDLKKEAMKDLEAGPDPVVEMANASNT

MESNLGMFLEEAENVKKEMGSIREILDQLQEANEESKTLHKPEALKSL

RNKINTDIVTVQKKARSIKSQLEEMDRANAANRRLSGYKEGTPIYRTRI

AVTNGLRKKLKELMMDFQGLRQKMMTEYKDTVGRRYFTVTGEYPDE

EVIDKIISDGSGGEEFLKRAIQEHGKGKVLETVVEIQDRHDAA

KEIEKSLLELHQVFLDMAVMVEAQGEQMDDIEHHVLNASHY

VKDGTKELKGAKGYQKSSRKWMCIGIILLLIIIL

VIVIPIATSFSHS*

>PtSYP122_Potri.016G068600

MNDLFSS

SFSRFSSEEAPPAHHVIQMSEAPSTG

GVNLDKFFEDVESIKDELKELERLNGNLQSAHEQSKTLHNSRAVKDL

RSKMDADVALALKKAKLIKVRLEALDRSNAANRTLPGCGAGSSSDRTRT

SVVNGLRKKLKDLMDGFNGLRQKISTEYRETVQRRYFTVTGENPDE

KTIDLLISTGESETFLQKAIQQQGRGRILDTINEIQERHDAV

KDLENNLKELHQVFLDMAVLVEHQGEQLDDIESNMQRANSF

VRGGTQQLQTARKLQKNTRKWTCYAIIILLIIIL

VVLLILRPWK*

>Phys_Pp3c1_23940

MNDLLSRS

FGRDGSNYVDLKKDSRHGDIELGNKASTG

PEIDMTKFFDEVAVIKGEMEKIKQLLSKVQDAHDESRTVTKAQGMKAL

RARMETDIRQVTKIAKAIKFKLEELDKANVENRRVIGCEEGAPTDRTRT

SITSTLRKKLKDLMGEFQILRQNMNDEYKESVERRYYTVTGEHADE

ETIDTIIETGNSETFLQKAIQEQGRGHVLETIKEIQERHDAV

KDIERSLLELHQIFMDMATLVDAQGEQLNDIEQQVNKASSF

IQRGTQQLQVAKNTQRSSRKWCLIAIILLIVLIL

VLAIPLLRSFGLL*

>Phys_Pp3c1_23960

MNNLLSNS

FGKAMNYVDLKKDIRRGDIELGEASIGD

GEVDMSQFFDEVGVIKSEMEKIKHCLEKVKDANEESRTVHKAQAMKAL

RSRMDADIAQVTKIAKSIKFKLEELDRANAANRRVRGCEEGTPTDRTRS

SITNTLRKKLKDLMGEFQILRQKMMEEYKETVERRYYTVTGQHADD

ETIENIIETGNSETFLQKAIQEQGRGQVLETIKEIQERHDAV

KDIERNLIELHQIFMDMATLVETQGEQLNDIESQVNKAASF

VERGTTQLKIAKNHQRNTRKWMCMGIALVIILIL

LILLPLLHTVGAI*

>Phys_Pp3c14_10430

MNDLFSRS

FGREGSNYVDLKKDSRKGDVELGEKSVAGG

PEIDMGPFFAEVDKIKSEMEKIKQLLAKVQGAHEESRTVSKAQAMKDL

RVRMDNDIKQVTKIAKTIKSKLEELDKANVENRKVRGCEEGTPTDRTRV

SITSTLRKKLKDLMGEFQILRQNMNEEYKETVERRYYTVTGKHADD

ETIETIIETGNSETFLQKAIQEQGRGQVLETIKEIQERHDAV

KDIERNLLELHQIFMDMATLVDAQGEQLNDIEQQMGKASSF

IARGAQNLQVAKNNQRSSRKWCCIAIILLIILLL

ILVIPILHSSGLI*

>Phys_Pp3c17_14090

MNNFLNNP

LGKAKNYVDLKKDARRGDIEMGDTAGSGGE

SEVDMTQFFEEVGVIKSEMDKIKQLLEKVKAANEESRIVHKAQAMKAL

RSRMDADIAQVTKIAKSIKLKLEDLDRANAANRRVRGCEEGTPTDRTRT

SITGTLRKKLKDLMGEFQTLRQKMMEEYKETVERRYYTVTGEHADD

DTIEHIIETGNSETFLQKAIQEQGRGQVLETIKEIQERHDAV

KDIERNLIELHSIFMDMATLVEAQGEQLNNIESHVNKASSF

IDRGTQQLKIAKDHQRNTRKWMCIGIALVIILIL

IILLPILKSVGAFDRSAPPPK*

>Phys_Pp3c18_17330

MNDLLA

RGLNRGVRYDEDDRQNDLENGVKHFPPSVQLTNMKA

NTDGMGDFLRHIEVVQAEVNKMNQQLVSLQNVNEKSKGVYRADELKAL

RAQMDAEIASATKRARFIKVKLEELDRSNIEHRQVRGCEAGTASDRQRI

SLTENQRKKVKELMDAFQSLRSKMVDGYKETIERRYYTITGEQADE

ETIENLISTGESETLLQQAIREQGRGPVLEAVREIQERLDGV

KEIEKHMLELHAIFMDISVLVSAQGDMINDIESNVQRSYSY

IKKGGEHLEVAKRYQMSKRRTTIICVLLLIIIIA

ILVLVLVLKFK*

>Phys_Pp3c19_4690

MSDLFKGI

WKGGKGGKVGNGEDTFGDVESGCVIQMTSLDA

DPSSMADFFREIGVVQGKVNDVKILLLKLQSAHEKTKGTHKASELKEI

RAEMDGDIESVTKAAQFMKFKLAELSKSNLANRQVKGCEEGTTTDRQRM

ALTNSQRKKLKELMDEFQALRATMMDEYKETITRRYYNVTGKQADE

ETTENMIRTGESETFLQQAIRQQGRGQLIETIREVQERHDGV

KEIERHFMEIHNIFTDISVLVDAQGQMVNEIQDNINRATSF

THRGADQLATARRRQIRKRKWTCVSILLLIVLIL

VLIIALKIAKIIP*

>Phys_Pp3c23_4510

MNDLLA

FSGRNNGRGYDDLESGGGPSTQMADMGG

GDQKMESFFAEVEKIKGDMDRIKQILIKLVDANEESKGVHRAPAMKEL

RGRMDADIAQVSKLARGIKGKLEDLDRKNADSRRVKGCEEGTPTDRTRM

TITNNQRKKLKDLMGDFQSLRERMMNEYKETIERRYYTVTGQQADE

ETIDQIIETGESETFLQKAIQEQGRGHVMETIREIQERHDSV

KEIEKNLLELHQIFMDMAVLVEAQGEQLNNIEAQVNRSSSY

VERGTTHLRVAKQHQRSKRKWTCIAIILLIILLL

IIIIPVLISNKVI*

>Phys_Pp3c24_8580

MNDLLSQT

FSARKAGGAYDDLESGPSTQMADLGG

GDQKLDGFFADVEKIKADMDKIKQLLLKLQEANEESKGVHRAPAMKAL

RERMDTDIAQVSKLARGLKGKLEALERGNAASRRVKGCEEGTPTDRTRM

TITINQRKKLKDLMGEFQVLRERMMNEYRETIERRYYTVTGQQADE

DTIETIIETGQSESFLQKAIQEQGRGHVMETIREIQERHDSV

KEIEKNLLELHQIFMDMAVLVESQGEQLNNIEAQVNRSASY

VERGTTHLRVAKSHQRSKRKWTCIAIILLVILLL

IIILPILKSNKVI*

>Phys_Pp3c24_8583

MNDLLSQT

FSARKAGGAYDDLESGPSTQMADLGG

GDQKLDGFFADVEKIKADMDKIKQLLLKLQEANEESKGVHRAPAMKAL

RERMDTDIAQVSKLARGLKGKLEALERGNAASRRVKGCEEGTPTDRTRM

TITINQRKKLKDLMGEFQVLRERMMNEYRETIERRYYTVTGQQADE

DTIETIIETGQSESFLQKAIQEQGRGHVMETIREIQERHDSV

KEIEKNLLELHQIFMDMAVLVESQGEQLNNIEAQVNRSASY

VERGTTHLRVAKSHQRSKRKWTCIAIILLVILLL

IIILPILKSNKVI*

>Phys_Pp3c3_13140

MNDLLADK

DSSANEKEEDLEAGPSPVPE

AGQDLSLFFQEVNDIKSSMAEIRKRFQKLQNTNEESKSVTKAANMTAL

KEKMEHDLDEISKVAQGLKGKIEAMDKALSYLLTVAQNLVNRKIKGCHEGSSTDRTRM

AITSTLKKKLKELMVDFQAIRRKFQVEYREVVERRVFTVTGQKVDE

SVIERLIDSGDGDQIFQKAIQEQGRGQILDTVAEIQERHDAV

RDIEKKLLELHQIFLDMAVLVEAQGDLLDNIETQVGKAVDH

IASGTTALQKAKSLQRGTRKCMCIGIVVLLVTIV

IIIVSVIQPWKNAV*

>Phys_Pp3c4_32180

MNDLLGDS

AAADGREVDLEAGPFDVPD

AGQNMSLFFQEVNDIKSSMAEIRKKFQKLQDTNDESKNVTKAPTMKAL

KEKMEQDLDDISKIAQGLKRKLEALDRANVANRKIKGCHEGSSTDRTRI

TISSTLKKKLKELMIGFQALRQRFQDEHREVVERRVFTVTGQKVDE

SVIERLIETGDSEQIFQRAIQEQGRGQILDTIAELQERHDAV

REIEKKLLELHQIFIDMAVLVESQGELLDSIETQVSKAVEH

VAAGTSALQKAKTLQRGTRKCTCVAIFLLLVTAI

IVLLAVIQPWKINATK*

>Phys_Pp3c6_25680

MS

FLDIEAG

GMPPGRRQQDSTQALASGVFQINTAVSSFKRLVSSLGTAKDTPAL

RDKLHKTRQHIGQLAKETGAKLKTASEHDHNRPVHGNKKLSDAKLAKDF

QAVLVEFQNAQKIAQEREKLYAPFVPEAALPTSQYSGEMKSAPEEN

QDQRAFYAAQRSQDFIQLENETVFNEAVIEEREQGI

REIHQQIGEVNEIFKDLAVLVHDQGYMIEDIDANVQGAEAA

TEQANRQLAKAAKSQKSGTTMTCLILVIVAMAVL

VLLLFLTR*

>PpSYP132b_Prupe.3G218900

MNDLLTD

SFVGDARAQPSGTNDIEMGRPVPSSF

DTGMDAFNKQIQEVEKQVDKLSGLLKKLKDANEESKSVTKASAMKAI

KKRMEKDIDEVGKIARGVKTKLEAISKDNLSNRQKPGCEKGTGVDRSRM

NMTNSLTKKFREIMIEFQTLRQRIQDEYREVVERRVITVTGTRPDE

ETIDNLIETGNSEQIFQKAVQEMGRGQVLNTVEEIQERHDTV

KEIEKKLLDLHQIYLDMAVLVEAQGEILDNIETQVTNAVDH

VQSGNTALQKAKKLQKNSRKWMCIAIIILLIIVA

IIVVSVLKPWKSG*

>PpSYP132a_Prupe.3G218900

MNDLLTD

SFVGDARAQPSGTNDIEMGRPVPSSF

DTGMDAFNKQIQEVEKQVDKLSGLLKKLKDANEESKSVTKASAMKAI

KKRMEKDIDEVGKIARGVKTKLEAISKDNLSNRQKPGCEKGTGVDRSRM

NMTNSLTKKFREIMIEFQTLRQRIQDEYREVVERRVITVTGTRPDE

ETIDNLIETGNSEQIFQKAVQEMGRGQVLNTVEEIQERHDTV

KEIEKKLLDLHQIYLDMAVLVEAQGEILDNIETQVTNAVDH

VQSGTDALQTAKSLQKKSRKCMMISIILLLIIAL

IIVLSILKPWKK*

>PpSYP125_Prupe.3G232300

MNDLFLSD

SFKRYKDLEHQVCIDDMQAGGEAWKE

TVNLDGFFKEIENVKHDMGAVVQLYKRLQEANKESKLIHDAKSMKEL

SARMDSDVEQVLKLVKIIKGTLETLELSNADQRKLPSCGAGSSSDRTRT

SVLSGLAKKFKDMMDDFQGLRTKMGSEYKETVQRRYFTITGEKANE

EMIENLISSGASETLLQKAIEEQGRGQVLDTVQEIQERHDAV

KEMEKSLIELHQVFLDMAALVDAQGQQLNDIESHVARANSF

VRRGNLQLEVAKDYQKNNRKWACIAIVIGTSAVIIL

LLPVYLHFKS*

>PpSYP124_Prupe.4G036000

MNDLFSS

SFKKYTDLKQQAYLDDMEAGKE

TVNLDKFFEDVENVKEDMRQVDKLYKQLQDANEESKTVHNAKTMKEL

RTRMDLDVEQVLKKVKIIKGKLEGLERSNATHRNLPGCGPGSSADRTRT

SVVSGLGKKLKDMMDDFQGLRGRMTSEYKETIERRYFTITGEKASE

ETIENLISSGESESFLQKAIQEQGRGQILDTISEMQERHDAV

KEIEKNLIELHQIFLDMAALVEAQGHQLNDIESHVMHASSF

VRRGTDNLQEAKEQQKKSRKWTCIAIILGIILVILL

LIPILIQVLPHML*

>PpSYP111_Prupe.7G000700

MNDLMTK

SFTNYVDLKKEAMKDLDLEAGHNNVEMSSSTTH

MHTDLGLFLEEAEKVKQEMGSVRDILGRLQQANEESKSLHKSEALQSL

RSRINADIMTVLKKTRTIRSQLEDMDRANAANRRLSAYKEGTPIYRTRI

AVTNGLRKKLKELMMDFQGLRQRMMTEYKETVGRRYFTVTGEHPDE

EVIEKIISNGDGNGGEEFLGKAIQEHGRGKVLETVVEIQDRHDAA

KEIEKSLLELHQIFLDMAVMVEAQGEQMDDIEHHVMNASHY

VKDGTKHLRTAKGYQRSSRKWMCIGVIILLLLIL

VIVIPIATSFANS*

>PpSYP131_Prupe.7G006600

MNDLLKD

SFEIPRGQASRDGDIELGTNGSMNS

GEFGLENFFKKVQEIEKQNEKLNKLLKKLQDAHEESKAVTKAPSMKSI

KQRMEKDVDEVGKIARWIKSKIEELDKENLANRQKPGCGKGTGVDRSRT

ATTLALKKKLKDKMAEFQTLRETIHQEYREVVERRVFTVTGTRADE

ETIERLIETGDSEQIFQKAIQEQGRGQIMDTLAEIQERHDAV

RDLERKLLDLQQIFLDMAVLVDAQGDLLDNIETQVSSAVDH

VQQGNTALQKAKKLQKSSRKWMCIAILILLIIVI

IIVVAVLKPWNSNKGA*

>PpSYP122_Prupe.7G093400

MNDLLSS

SFSGASPEHHVIEMTPSK

GDDLGKFFQDVDAVQSELDELQKLNKSLRSSHEQSKTLHNAKAVKEL

RTRMDADVHLALKKAKILKVRLDALDRSNAANRSLPGCGPGSSSDRTRT

SVVNGLRKKLKDSMDSFNDLRQKISSEHRETVQRRYFTVTGDNPDE

KTVDLLISTGESETFLQKAIQEQGRGRVLDTISEIQERHDGV

KAMERNLNELHQVFMDMAVLVQAQGEQLDDIQSHVERANSY

VHAGNQQLQKARFLQKNTRKWTCYGIIILLIIVGI

ILASTLPN*

>PpSYP121_Prupe.7G093500

MNDLFSG

SFSRFRSETNTNDDHVIEMSSAGSST

GGVNLDKFFGDAESVKDELKELERLHQNLQSSHEHSKTLHNAKAVKDL

RSRMDADVTLALKKAKVLKVRLEALDRSNAANRSLPGCGPGSSSDRTRI

SVVNGLRKKLKDSMDSFNSLRQKISSEYRETVQRRYFTVSGENPDE

KTLDRLISTGESETFLQKAIQEQGRGQVLDTIHEIQERHDAV

KDMEKNLQELHQVFLDMAVLVQAQGEQLDDIESHVARANSF

VRGGTQQLNKARFYQKNTRKWTCFLIILLLVIAL

IVILSLKPWNWNNGNNNNNNNPTPSPPPPA*

>PpSYP112_Prupe.7G156800

MNDLMTK

SFLNYVELKKQAQKDLEADLDVEAGQGQLNPS

DQENLSQFFQEVTPIKSDMEEITNLLYDLQSLNEEAKSTHSAKILRGL

RDRMESDMVAVLRKARIVKARLEALDKSNVISRKISDAYKEGSTVDRTRM

SITCGLRVKLRDMMDNFQSLREKIVADHKEDLKRRYYSAIGELPSD

EMIENMISGSLKVELFEGKTELDMGNKVRHEAV

MDIQRSLNKLHQVFLDMAVLVEAQGEDIDNIEENVANASYF

INGGTNSLYYASQMKKKSKKWVYWVWAVVLIILLVCVIS

TLAS*

>PpSYP113_Prupe.8G094700

MNDLMTK

SFLSYVDLKKQAMKDLESEPDLEMGKLGPA

DEQNLTQFFEQVTAIKADMEEITNLLLDLQDLNEETKSTHSAKVLRGL

RDRINADMVAILRKAKGIKARLESLDRSNVANRNVSVEYKEGSPVDRMRI

SVTNGLRAKLRDMTNDFQSLREQIVKEHKEGLKRRYYNATGEEASE

EVIDKMILESGQIKVFEGKAELAMENQQRHEAL

KDLQRSLTELHQVFLDMAMMVEKQGEQMDDIEQNVADAGAY

IHGGTNALFSAKQMKQRRRRWVCWIGALVLLVLLVEVPSSTTD

NKEIITMKVKG*

>SiSYP124_Seita.1G039700_

MNDLFTSG

SFKKYADLKQQVALDDLESGGGGEAEGP

DLDRFFEDVEVVKEDLRGLEALHRRLQSAHEEGKTAHDAAAVRVL

RARADADADQVLRHARAVKARLEALDRVNAASRKLPGCGPGSSTDRTRT

SVVIGLGKKLKDLMDDFQGLRTRMAAEYKETVARRYYTVTGEKAED

STVEALIASGASETFLQKAIQQGQGQAAGRGQVLDAVSEIQSG

TARLRELHQVFLDMAALVEAQGHQLNDIESHVARASSF

VLRGAVELETAREYQKGSRKWACVAVVAGAVLVAVI

VLPIVINLHLLTVR*

>SiSYP125_Seita.4G229100

MNDLFSSS

SFKKYADASPAPSGGDMEAGGESVV

NLDKFFEDVEAVKEDMRGLEGMYKGLQSTNEETKTAHDARTVKSL

RARMDKDVEQVLRRAKAVKSKLEELDRSNATSRKVPGCGPGSSTDRTRS

SVVAGLGKKLKDLMDDFQGLRARMAAEYKETVARRYFTVTGEKAED

STIEALISSGESESFMQKAIQEQGRGQVMDTISEIQERHDAV

KDIERSLMDLHQVFLDMAALVEAQGHQLNDIESHVAHASSF

VRRGTVELETAREYQKSSRKWMCFAVLAGILLIAVL

ILPVLVNLRILTLPTRR*

>SiSYP111_Seita.7G214300

MNDLMTK

SFMNYVDLKKAAMKDVEAGGDGIELPESGAGGV

TDERLRGFFQEAEAVKAEMAAIRDALDRLHAANEEGKSLHQADALRAH

RGRVNADIVAVLRRARDIRARLESLDRANAAQRRLSAGSREGTPLDRTRT

AVTAGLRKKLKDLMLDFQALRQRMMSEYKETVERRYYTLTGEVPEE

EVIERIISDGRGEELLGAAVAEQGKGAVLAAVHEIQDRHDAA

REVERSLLELHQVFLDMAVMVETQGEKLDDIESHVANASHY

VQGGNKELGKAREYQRGSRKWLCIGIIILLILVL

LVIVPIATSFRKS*

>SiSYP121_Seita.9G063400

MNSLFSS

SWKRGGGGDGGDIESGADGVEMSAPPGAA

AGASLDKFFEDVESIKDELRDLERIQRSLHEANEGSKSLHDAAAVREL

RSRMDGDVASAIKKAKVVKLRLESLDRANAANRSVPGCGPGSSTDRTRT

SVVAGLRKKLRDSMEAFSSLRSRIAAEYRDTVARRYFTVTGSQPDE

ATLDALAESGEGERFLQRAIAEQGRGEVLGVVAEIQERHGAV

AELERSLLELQQVFNDMAVLVAAQGEQLDDIEGNVGRARSF

VDRGREQLQVARKHQKSTRKWTFIAILIVLVIVLV

IVLPIVLNNTKK*

>SiSYP112_Seita.9G101200

MNDLMTK

SFMSYVDLKKAAMKDLEAGGDGIELPESGAGGV

TDERLRGFFEEAEAVKAEMAAIRDALDRLHAANEEGKSLHQADALRAH

RGRVNADIVAVLRRARDIRARLESLDRANAAQRRLSAGCREGTPLDRTRT

AVTAGLRKKLKDLMLDFQALRQRMMSEYKETVERRYYTLTGEVPEE

EVIERIISDGRGEELLGAAVAEHGKGAVLAAVHEIQDRHDAA

REVERSLLELHQVFLDMAVMVETQGEKLDDIESHVANASHY

VQGGNKELGKAREYQRSSRKWLCIGIIILLLLIL

LVIVPIATSFRKS*

>SpSYP124_Spipo0G0047600

MNDLFSAG

SFKNYTDLKRQVQMDDMEAGGATGD

LEKFFEEVEKVKEDLRGLEALHRRLQEANEESHTVHSAAAMKAL

RSRMDGDTQKVLRQAKLVKAKVEALDRSNAANRRLPGCGAGSSADRTRT

AVVGGLGKKLKTLMDEFQALRGKISAEYADTVRLRYYTVTGGHADE

ATVERLISTGESETFMQRAIQEQGRGQILDTITEIQERHESV

QDIERSLLELHQVFLDMAALVEAQGHQLNDIESHVAHANSF

VRRGTGQLETAKEYQKSSRKWTCIAIVLGAVLVLVL

VVPLLSTITNLANMA*

>SpSYP131_Spipo4G0027300

RHSIGD

SFEIPRGQPSRDQDIEMGVQVSTNS

AEQGLESFFKQVQDIEKMIDKLSKLLKKLQDANEESKAVTKASIMKAI

KQRMEKDIDEVGKIARIAKSKIEELDKDNLQSRQKPGCGKGSGVDRSRT

ATTVALKKKLKEKMSEFQTLRENIQQEYREVVERRVFTVTGSRPDE

ETIDRLIETGNSEQIFQKAIQEQGRGQVLDTLAEIQERHDAV

REIERRLLELQQIFLDMSVMVEAQGDLLDNIESQVSSAVDH

VQSGTTALQKAKRLQKSSRKWMCIAIIILLIIVA

IIVVAVVKPWASGK*

>SpSYP111_Spipo9G0044000

MNDLMTK

SFMSYVDLKKEALKDLEAGGGDDFGDVEMWSAAT

ADGNLRRFFEEAGRVREEMDEIRELLVRLQEANEEGRSLHKPESLKSL

RARINGDVVKVLKKARDIRNVLEEMDRENAVNRRLSGCREGTPVDRTRT

SITNGLRKKLKDLMMDFQTLRQKMMSEYKETVERRYFTVTGQVPEE

EMIERIISHGESEDLLKKAIEEHGRGKVLATVHEIQDRHDAA

KEIERSLLELHQVFLDMALMVEAQGEQMDDIEHHVTHAADY

VKGGVKQLATAKQHQRSSRKWLFIGLLLLLILIL

VVVVPVATSLRSS*

>TcSYP112_Thecc1EG000354

MNDLMTN

SFVSYVELKKQAQKDLEADLEIEEGQGQLNPA

DEKNLSQFFREVESIKITMEEITNLVFDLQTLNEETKSSHSAKVLRGL

RERMEADTVFILRKAKIVKTRLESLDRSNVTNRRLSEAYKEGSCVDRTRM

SVTNSLRVKLRQMMNDFQALREKMMLDHKEDLKRRYYNATGELPSE

AVLEKVVSGGEKDQLFAEKVEMDLRSKERHEAM

LDIQRSLQRLHQVFLDMAVLVEAQGENMDDIEEHVANAGNF

VSGGTNSLYYANQMKKKKKAWVYWVWAVGLIVLLVCIIS

MLAS*

>TcSYP124_Thecc1EG007960

MNDLFSS

SFKKYSDLKQQAYSDNLEAAGEAWKE

TVNLDKFFEDVDNVKDDMRVVKQLYKRLQESNEETKTAQNAKAMKDL

RARMDSDVEQVLKRVKVIKGKLEALERSNAAHQKLPGCGPGSSAYRTRT

SVVSGLGNTLKDMMDDFQGLRAKMTAEYKGTVERRYFTVTGQKADE

EMIENLIASGQSETFFQEAIQDQGRGQILDTISEIQERHDAI

KEIEKNLVELHQLFLDMAVLVEAQGLQLNDIESHVAHASSF

VMRGTEQLEVAKEYQKNSRKWGCFAIVLGVFLVVVI

LFPVLSSDIIKNT*

>TcSYP121_Thecc1EG022978

MNDLFSG

SFSRFRSEEASPDHHVIQMTQSSSTS

GGVNLDKFFEDVESIKDELKELERLNDNLSSSHEQSKTLHNAKTVKEL

RAKMDADVAMALKKAKLIKVRLEALDRSNAANRSLPGCGPGSSSDRTRT

SVVNGLRKKLKDSMESFNGLREKISSEYRETVQRRYFTVTGENPDE

RTIDLLISTGESETFLQKAIQEQGRGRILDTINEIQERHDAV

KDMEKNLKELHQVFLDMAVLVQAQGEQLDDIESQVNRANSF

VRGGTERLQTARNYQKNTRKWTCYAIILLLVIILF

VVLFTVRPWENNGGGGGGNSSPSQNPTTTAPPPPTA*

>TcSYP111_Thecc1EG026036

MNDLMTK

SFMSYVDLKKEAMKDLEAGPDYDLEMSSTANT

MDQNLGLFLEEAEKVKQEMAVIRELLGKLQESNEESKSLHKQESLKAL

RNKINNDIVTVQKKARTIKSQLEEMDRANAANKRLSGCKEGTLAYRTRI

AVTNGLRNKLKELMMDFQGLRQKMMTEYKETVGRRYYTVTGENPDE

EIIEKIISDGNGGEEFLTRAIQEHGRGKVLETVVEIQDRHDAA

QEIEKSLLELHQVFLDMAVMVEAQGEQMDDIEHHVMNASHY

VKDGSKELNTAKQYQRSSRKWMCIGIILLLLIVL

VIIIPIATSFSSS*

>TcSYP125_Thecc1EG029975

MNDLISI

SFKKYTDLKQQAYLDDMEAGNE

SVNLDKFFEDVENVKEDLKNVERLYKALQDANEESKTVHNAKTMKQL

RARMDTDVEQVLKRVKIIKGKLEALERSNAASRSIPGCGPGSSADRTRT

SVVSGLGKKLKVLMDDFQGLRSRMQSEYKETVERRYFTITGQKADE

DTIENLISSGESESFLQRAIQEQGRGQIMDTISEIQERHDAV

KEIEKNLIELHQIFLDMAALVEAQGHQLNDIESHVAHASSF

VRRGTENLQEAREYQKNSRKWTCIAILAAAVLIFVL

LFPLLPTIINLL*

>CpSYP121

MNDLFSG

SFSRFRSEDASPDRNHRTVEMTDSAVAT

GGVNLDKFFEDVESVKDELKELERLNDSLQSSHEKSKTLHNAKAVKDL

RARMDADAAAALKKAKIIKVRLEALDRSNAANRSLPGCGPGSSSDRTRT

SVVNGLRKKLKESMDSFNSLRERISSEYRETVQRRYFTVTGENPDE

KTLDRLISTGESETFLQKAIQEQGRGRILDTINEIQERHDAV

REMEKNLKELHQVFLDMAVLVQAQGEQLDDIESQVARAHSF

VRGGTQQLTTARVYQKSTRKWTCYAIIILLVIILF

VVLFTVKPWERNGGGNNSGPSPSSGQSTTPPAGQSSSPSPPGR

R*

>CpSYP124

MNDLFSS

SFKKYTDLKQQAFMDDMEAGGE

TMNLDKFFEDVENVKEDMRTVENLLKKLQDANEESKTVHNAKTMKEL

RARMDMDVEQVLKKVKMIKGKLEALERSNATSRNVPGCGPGSSTDRTRT

SVVSGLGKKLKDLMDGFQGFRAKMQAEYKETVERRYFTITGQKADD

QTIENLISSGESENFLQKAIQEQGRGQILDTISEIQERHDAV

KEIEKNLLELHQVFLDMAALVEAQGHQLNDIESHVAHANSF

VRRGTENLQEAREYQKASRKWTFIAIILVIVLIIVL

LLPLLPSILAVL*

>CpSYP111

MNDLMTK

SFTSYVDLKKEAMKDLEAGPDYDLEMSATANS

MDQNLGLFLEEAEKVKQEMGVIREILGRLQESNEESKSMHKSESLKLL

RHKINNDILTVLKKAKSIRSQLEEMDRANIANKRLSGCKEGTPIYRTRI

AVTNGLRKKLKELMMEFQGLRQKVMTEYKETVGRRYFTVTGEYPDE

EIIDKIISDSNGGEEFLSRAIQEHGKD*

>CpSYP112

MNDLMTK

SFLNYVELKKQAQRDLDSEQDIENGQLNKA

EEENLCQFFQEVEAIKVEIEEIPSLLLDLQALNEESKSAHSAKLLRGL

RDRMESDVVSVLRKAKIVKARLESLDRSNALNRTVSENYREGTCVDRTRI

FITNGLRVKLREIMNDFQSLREKIVSDHKEDLKRRYYNATGEKPCE

EIIEKMMVSGGEKVELLEGKTEVDLRNEEKHEAV

MDLERSLNRLHQVFLDMAVLVETQGEKMDDIEENIVNAGQF

INGGTNNLYYANQMKKKKAASFFWVWAVGLIVLLVCIIS

MLAS*

>AtSYP125

MNDLFSN

SFKKNQAQLGDVEAGQE

TMNLDKFFEDVENVKDDMKGVEALYKKLQDSNEECKTVHNAKKVKEL

RAKMDGDVAMVLKRVKIIKQKLEALEKANANSRNVPGCGPGSSTDRTRS

SVVSGLGKKLKDLMDSFQGLRARMNNEYKETVERRYFTITGEKADE

QTIDNLIASGESENFLQKAIQEQGRGQILDTISEIQERHDAV

KEIEKNLLELHQVFLDMAALVEAQGQQLNNIESHVAKASSF

VRRGTDQLQDAREYQKSSRKWTCYAIILFIVIFILL

LIPLLPHIMLMLK

>AtSYP124

MNDLFSS

SFKKYTDLKQQAQMDDIESGKE

TMNLDKFFEDVENVKDNMKGVETLYKSLQDSNEECKTVHNAKKVKEL

RAKMDGDVAQVLKRVKMIKQKLEALEKANANSRNVSGCGPGSSTDRTRT

SVVSGLGKKLKDLMDSFQGLRARMNAEYKETVERRYFTITGEQADE

QTIENLISSGESENFLQKAIQEQGRGQILDTISEIQERHDAV

KEIEKNLIELHQVFLDMAALVESQGQQLNDIESHVSKASSF

VRRGTDQLQDAREYQKSSRKWTCYAILLFIVVFALL

LIPALPHIMLMLK

>AtSYP112

MNDLMTK

SFLSYVELKKQARTDMESDRDLEKGEDFNFDFSPA

DEENLSGFFQEIETIKTLIEEITHLLLDLQNLNEETKSTHSTKILRGL

RDRMESNIVTISRKANTVKTLIETLEKRNVANRTSFKEGSCVDRTRT

SITNGVRKKLRDTMSEFHRLRERIFADYREDLKRKYFLATGEEPSN

EDMEKMISGSGSCSDLVKTFEVKPEMDLKTKERHEAV

NDIKRSLNRLHQVFLDMAVLVETQGDRIDDIEANVANAGSF

VSGGTNSLYYANQMKKKTKSWVLWVSILGVLILLVCVIS

MLASR

>AtSYP121

MNDLFSS

SFSRFRSGEPSPRRDVAGGGDGVQMANPAGST

GGVNLDKFFEDVESVKEELKELDRLNETLSSCHEQSKTLHNAKAVKDL

RSKMDGDVGVALKKAKMIKVKLEALDRANAANRSLPGCGPGSSSDRTRT

SVLNGLRKKLMDSMDSFNRLRELISSEYRETVQRRYFTVTGENPDE

RTLDRLISTGESERFLQKAIQEQGRGRVLDTINEIQERHDAV

KDIEKNLRELHQVFLDMAVLVEHQGAQLDDIESHVGRASSF

IRGGTDQLQTARVYQKNTRKWTCIAIIILIIIITV

VVLAVLKPWNNSSGGGGGGGGGGTTGGSQPNSGTPPNPPQARR

LLR

>AtSYP122

MNDLLSG

SFKTSVADGSSPPHSHNIEMSKAKVSGGSCH

GGNNLDTFFLDVEVVNEDLKELDRLCHNLRSSNEQSKTLHNANAVKEL

KKKMDADVTAALKTARRLKGNLEALDRANEVNRSLPESGPGSSSDRQRT

SVVNGLRKKLKDEMEKFSRVRETITNEYKETVGRMCFTVTGEYPDE

ATLERLISTGESETFLQKAIQEQGRGRILDTINEIQERHDAV

KDIEKSLNELHQVFLDMAVLVEHQGAQLDDIEGNVKRANSL

VRSGADRLVKARFYQKNTRKWTCFAILLLLIIVVL

IVVFTVKPWESNGGGGGGAPRQATPVQAQPPPPPAVNRRLLR

>AtSYP123

MNDLISS

SFKRYTDLNHQVQLDDIESQNVSLD

SGNLDEFFGYVESVKEDMKAVDEIHKRLQDANEESKTVHDSKAVKKL

RARMDSSVTEVLKRVKMIKTKLVALEKSNAAQRKVAGCGPGSSADRTRT

SVVSGLGKKLKDMMDDFQRLRTKMATEYKETVERRYFTVTGQKADE

ETVEKLISSGESERFLQKAIQEQGRGQVMDTLSEIQERHDTV

KEIERSLLELHQVFLDMAALVEAQGNMLNDIESNVSKASSF

VMRGTDQLHGAKVLQRNNRKWACIATILAIVVVIVI

LFPILFNTLLRP

>MtSYP121

MNNLFTG

SFSRFRSEEVSPDRHHVIEMTDGAGTRTGAGAG

GRINLDKFFDDVEGVKDDLKELESIHQRLSKTNEQTKTVHDAKGVKEL

RSRMDEEVSAALKKAKMVKLKLEKLERSNAANRNLEGCGPGSSSDRTRS

SVVNGLKKKLKDSMESFNRLREVITTEYRETVQRRFFTVTGENPDD

KTLDLLISTGESETFLQKAIQEQGRGRILDTINEIQERHDAV

KDLEKSLLALHQVFLDMTVMVQFQGEQLDDIESHVARASSF

VHTGTDQLQTARKHQKNTRKWACYCIILLLIIVLI

VVLATVKPWQHNDSGGGGGNQPAPAQTPPSPPPPSGGT

>MtSYP125

MNDLFSS

SFKKYSDLKEQAQIDDVEAGKE

SINLDKFFEDVENVKEDMRLVEKLYRKLQEANEESKIVHNAKTMKDL

RARMDKDVEQVLKRVKIIKGKLEALERSNAANRNIPGCGPGSSADRTRT

SVVSGLGKKLKDMMDDFQGLRARMQQEYKETIERRYFTITGEKADE

DTIENLISSGESETFMQRAIQEQGRGQIMDTISEIQERHDAV

KEIEKNLIELHQVFLDMAALVESQGQQLNNIESHVAHASSF

VRRGTEQLHEAREHQKDSRKWTCYVILLAIVLVIVL

LFPLLMSILPHLFL

>MtSYP124

MNDLFSN

SFKKYTDLKEQAYLDDVEAGNRE

TVNLDKFFEDVENIKEDMKTIEQLYRKLKEANEESKTVHNAKTMKEL

RARMDKDVEQVLKRVKIIKGKLEALDRSNAANRNIPGCGPGSSADRTRT

SVVSGLGKKLKDMMDDFQGLRARMQLEYKETVERRYFTITGEKADE

ETIENLISSGESENFMQRAIQEQGRGQIMDTISEIQERHDAV

KEIEKNLIELHQVFLDMATLVESQGQQLNNIESHVAHASSF

VRRGTEQLQEARVQQKNSRKWTCYAIILGIVLVVVL

LFPLLTSLLPHLLK

>MtSYP111

MNDLMTK

SFTNYVDLKKAAMKDEVDLEAGLHQKQGGVELTRSSLTH

LETDMNLFLEEAEKVRTEMGEIRDILTKLEQANEESKSLHKTEALKAL

RERINTDIVTVLKKAKCIRTQLEEMDRANAANRRLSGLKDGSPAIYRTRI

AVTNGLRKKLKELMMEFQGLRQKMMSEYKETVGRRYYTVTGEHADE

EVIEKIISNGDDESFLGKAIQEHGRGKVLETVVEIQDRHDAA

KEIEKSLLELHQIFLDMAVMVEAQGEKMDDIEHHVLHASHY

VKDGTKNLHGAKQYQKSSRKWMCIGIILLLILVL

IIVIPIVTSFSSS

>MtSYP122

MNDLFSG

SFSRFRNSDQVSPDNHHHVIEMSSPNTAQ

TGVHLDKFFEEVEGVKEELKELDRLYESLRVSHERSKTLHSAKAVKDI

RSKMDADVALALKKAKLVKLRLEALDRSNEASRSLPGAGPGSSSDRTRT

SVVSGLRKKLKDSMDSFNNLRQQISSEYRETVQRRYYTVTGENPDD

KTVDLLISTGESETFLQKAIQQQGRANIMDTIQEIQERHDTV

KEIERNLMELHQVFMDMSVLVQSQGEQLDNIESHVARANSY

VRGGVQQLHVARKHQMNTRKWTCIAIIILLIIILI

IVLPIVLKK

>ATSYP111

MNDLMTK

SFMSYVDLKKAAMKDMEAGPDFDLEMASTKADK

MDENLSSFLEEAEYVKAEMGLISETLARIEQYHEESKGVHKAESVKSL

RNKISNEIVSGLRKAKSIKSKLEEMDKANKEIKRLSGTPVYRSRT

AVTNGLRKKLKEVMMEFQGLRQKMMSEYKETVERRYFTVTGEHAND

EMIEKIITDNAGGEEFLTRAIQEHGKGKVLETVVEIQDRYDAA

KEIEKSLLELHQVFLDMAVMVESQGEQMDEIEHHVINASHY

VADGANELKTAKSHQRNSRKWMCIGIIVLLLIIL

IVVIPIITSFSSS

>MtSYP112

MNDLMTK

SFLSYVDLKKQAQKDFEDVDLELGNLNPT

QDPNLSQFFQEVEAIKVEMEEITNLLLDLQQLNEETKSTHSAKVLRGL

RDRMDSDMVAVLRKANIIKARLEALQKSNIANRSISECYKEGSPIDRTRV

SVTNGLKVKLRDMMNDFQSLRDKIVLDHKEDLKRRYYTVTGEVPSD

EVMEKMISGSLKVEFLAGKTDADMRTQVRHEAA

MDIQRSLNKLHQVFLDMAILVETQGEKVDNIEDNVVNAAQY

IHGGTSNLYYASQIKKRNRKWVYWVIAVLLIVLLVCLIA

MLAS

>MtSYP113

MNNLMTK

SFLSHVELKKQAQKDFEDVDLELGNLNPT

QDPNLSQFFQQVEAIKVEMEEITNLLLDLQQLNEETKSTHIAKVLRGL

RDRMDSDMVAVLRKANIIKARLEALEKSNIANRSISEW

DMMNDFQSLRDKIVLDHKEDLKRR

>SlSYP121a

MNDLFSG

SFSRYRENDHDQDSHGIEMGDT

GGVNLDKFFEDVEAIKDELKNLEKIYAQLQSSNEKSKTLHNAKAVKDL

RSKMDDDVSLALKKAKFIKVRLEALDRSNASNRSLPGCGPGSSSDRTRT

SVVNGLRKKLQESMNQFNELRQKMASEYRETVQRRYYTVTGENPDE

AVLDTLISTGQSETFLQKAIQEQGRGQVMDTIMEIQERHEAV

KEIERNLKELHQVFLDMAVLVESQGEQLDDIESQVNRANSF

VRGGAQQLQVARKHQKNTRKWTCFAIILLLIIIL

IVVLSIQPWKK*

>SlSYP121b

MNDLFSG

SFSRFRNEEQSPNQESAGIQMRQQT

GGVNLDKFFEDVETIKDELKELEKIHTQLHNSHEQSKTLHNAKNVKDL

RKKMDNDVSLALKKAKFIKVRLEALDRSNAANRSVPGCGPGSSSDRTRT

SVVNGLRKKLQESMNQFNELRQRMASEYRETVQRRYFTVTGENPDE

GTLDTLISTGQSETFLQKAIQEQGRGQVMDTVMEIQERHEAV

KELERNLKELHQVFMDMAVLVESQGAQLDDIESQVNRANSF

VRGGAQQLEVARKHQKSSRKWTCIAIIILLIIVL

VVVLSIQPWKK*

>SlSYP124a

MNDLFSN

SFKKYQDLKKQTEVDDLEGGQDGQPGTE

SIDLAKFFEDVENVKEDMKDVEKFHKKLQESNEESKLVHNAKTVKEI

RSRMDSDVSQVLKRVKMIKGKLEALERSNAAHRKISGCGPGSSADRTRT

SVVSGLGKKLKVLMDDFQGLRTRMNDEYKETVARRYFTVTGEKADD

GLIENLISSGESESFLQKAIQEQGRGQIMDTISEIQERHDAV

KEIEKNLIELHQIFLDMAALVEAQGQQLNDIESHVAHASSF

VRRGTEQLQEAREIQKSSRKCACFAVFLIILLIILL

TFPLWWPLVASKIL*

>SlSYP124b

MNDLFSP

SLKKYQDLKQQVQMDDLELGTGGTGPSHNE

SIDLAKFFEDVENVKEDMKEVEKLHKRLQDSNEESKTVHSAKKVKDI

RARMDSDVTLVLKRVKIIKGKLEGLERSNVANRKNLGCGPGSSADRTRT

SVVSGLGKKLKVLMDDFQALRAKMNSEYKDTVARRYFTVTGENADD

ELIDNLISSGESESFLQKAIQEQGRGQIMDTISEIQERHDAV

KEIEKNLIELHQIFLDMAALVEAQGQQLNDIESHVAHASSF

VRRGTEQLTEARELQKSSRKCTCIAILLIILLIIKLHLQIV

SMFLKLLGHRHYSSVTFFTNPPSKESEIIHLCRSDLLSQAIKLLKSTEKISSKPIVYATL

IQTCTKSHSFNHGVQFHTHVIKTGIET

>SlSYP111

MNDLMTK

SFTSYIDLKKAAMKDVEASPDLEMGMTQ

MDQNLTAFLEEAEKVKLEMNSIKEILRRLQDTNEESKSLHKPEALKSM

RDRINSDIVAVLKKARAIRSQLEEMDRSNAINRRLSGCKEGTLVDRTRS

AVTNGLRKKLKELMMDFQGLRQRMMTEYKETVGRRYFTVTGEHPDE

EVIDKIISSGNGQGGEEFLSRAIQEHGRGKVLETVVEIQDRHDAA

KEIEKSLLELHQIFLDMAVMVEAQGEKMDDIEHHVVNAAQY

VNDGAKNLKTAKKYQKSSRRCMCIGAIILLILIL

VVIIPIATSFTKS*

>SlSYP112a

MNDLMTK

SFLSYMELKKQAHLDLETERDLEMGQLSRT

DEDNLSNFFREIEAVKGDIQEITNLLMDLQNLNEETKTTHGPKVLRGI

RDRMDSDMVSVLRKAKIVKAKLEALDKSNVGNRKLSVAYAQGSVVDRTRV

SMSNGLRVKLRDIMNDFQALREKILSDYKDCLRRRYYNETGKEPNE

EVIEKMVSGESGKVQIFAAKTEMNLDDKDRHEAV

MDIKKSLDKLHQVFLDMAVLVETQGEQIDDIEHNMAIAGSF

ISGGTNSLFYAKQQQKKGRAWICWVWAVLLIILVVCLIA

TLSS*

>SlSYP112b

MTK

SFLSYVELKKQAMMDVEAGPDIEMGQLDPT

DERNLSKFFEEVAVIKSDMEEINNLLVNLQDLNRKTKSAPSAKILQGH

RDQINSDIITVLRKAKMIKTRLELLDKSNLDNRGVSGSPVDRTRI

SVTNGLRIKLRDMMNDFQCLRENIVAEHKEGLRKQYSNANGKEPSE

EAIEKMMQERVIEKGVVENQDRHEAV

KEIQKSLVELHQVFLDMAVMVETQGDQMNNIEQNVVNAGGY

VNGGMKELDRANRMKRTRTWACWIGALVLVFLLICLIA

ILF*

>MtSYP123

MNDLLSG

DNNHHHIIQMAETYTAP

VQTLEKFFQEVESLKEELKTLEKLHTSLKNSHEKSKTLHSAQAVKEL

RSAMDSDVTISLKKARLIKVRLESLDRSNEASRSLPDCGPGSSSDRTRL

AVVSGLRKNLKDSMESFNGLREQISSEYRETVKRRYFAVNEEKADD

KTVDLLISTGESETFLQKAIQKQGRATMMEKIQEIEERHGAV

KEIERNLKELHQVFLDMAVLVQSQGEELNDIESQMMRANSY

VRGGVQQLHVARKNQKNTREWTCFAILLLLIIALV

IILPIVLKNN*

>ShSYP131

MNDLLTD

SFVIPRDQSRGNRDIESGLHRQMNS

GEMGLETFFNQVQVIEKQYEKLNAHLRKLQNSHEESKAITKAAAMKEI

KRKMEKDVDEVGIIARSIKAKIEELDRQNLESRKKAGCGQGSGIDRSRT

ATTVALKKKFKDRMSEFQTLREDIHQEYREVVERRVFTVTGTRVDE

ETIDKLIETGDSEQIFRKAIQEQGRGQVLDTLAEIQERHDAV

RDLEKKLLDLQQIFLDIAVLVDAQGDMLDNIESQVSKAVDH

VQRGTNTLQKAKSLQKNSRKWMCLAIFIFLIIVA

VMVVNVLKPWQNK

>ShSYP132

MNDLLTD

SFAIPRDEGNRNGDIEMGMERQTNS

EDLGMDNFFKQVQEIEKQYEKLNILLRKLQDAHEESKAVTKAANMKAI

KKRMEKDVDEVGKIARSIKSKIEELDKENLANRQRPGCGKGSGVDRSRT

ATTVALKKKFKDRMSDFQTLRENIHQEYREVVERRVFTVTGTRADE

ETIDKLIETGDSEQIFQKAIREQGRGQVMDTLAEIQERHDAV

RDLEKKLLDLQQIFLDMAVLVDAQGDMLDNIESQVSSAVDH

VQSGNTALQKAKSLQKNSRKWMCIAIIILLIIVA

IVVIGVLKPWQNKNGGLAFYPSV

>BvSYP131

MGVNLQNS

GELGLQNFSKKVQEVDKQYDKINELLRKLQDAHEESKAVTKASAMKAI

KKRMEKDVDEVQKIARLIKSKIEELDRDNLSSMQKPGCGKGTAIERART

AQTIALKKRLRDKMAEFQVLRENIHQEYREVVERRVYTVTGQRADE

EKIDQLIETGDSEQIFQKAIQEQGRGQVMDTLAEIQERHDAV

RDVERKLLELQQIFLDMAVLVDAQGDLLDNIESQVSNAVDH

VNTGNTALQKAKSLQKNSRKWMCIAIIILLIIVA

VIVVGVLKPWQQKGGA

>MtSYP131

MNDLLTE

SFEIPRGRGGGDIELGEYGRNS

GELGLDSFFKKVQELEKQYVKLDNLLRKLQDAHEETKAVTKAPAMKAI

KQRMEKDVDEVKKTAYFLKTKVEELDKENLANRQKPACGKGSAVDRSRT

ATTIALKKKLKEKMAEFQILREAIHEEYREVVERRVFTVTGARADE

ETIDRLIETGDSEQIFQKAIQEQGRGQVMDTLAEIQERHEAV

RDVERKLLDLQQTFMDIAVLVDAQGDMLDNIESQVSSAVDH

VQQGNNSLQKAKKMQRNSRKWMCIAIMILLIVVV

IIVVSVIKPWVTKKGA

>CsSYP131

MNDLLSD

SFEIRRGQPSGGRDIELGANAPTSA

GDQGMGDFFKKVQEIEKQNEKLDRLLRKLQDSHEESKAVTKAPAMKAI

KQRMEKDVDEVGKVARYVKTKVEELDRENLSNRQKLGCGKGSGVDRSRT

ATTLALKKKLKDKMTEFQILREKIHQEYRDVVERRVFTVTGARADE

ETIEKLIETGDSEQIFQKAIQEQGRGQVMDTLAEIHERHSAV

RELERKLLELQQVFLDMAVLVEAQGDMLDNIESHVTSAVDH

VQQGNTALQKAKKLQKNSRKWMCIAIIILLIIVV

VIVVGVLKPWNNGKGA

>TcSYP131

MNDLLTE

SFEIPRGQGSRGGDIELGAQMNS

GELGLQNFFKKVQDIDKQYEKLDKLLKKLQDAHEESKAVTKAPAMKSI

KQRMEKDVDEVGKISRFIKAKIEELDKENLANRQKPGCGKGTGVDRSRT

ATTLAIKKKLKDKMAEFQTLRETIHQEYREVVERRVFTVTGARPDE

ETIEKLIDTGDSEQIFQKAIQEQGRGRIMDTVSEIQERHEAV

RDLERKLLELQQIFLDMAVLVDAQGDMLDNIESQVSSAVDH

VQSGNTALQKAKKLQKNSRKWMCIAIIILLLIVV

IIVVAVIKPWSSNKGLCYKTPISRVCVCWLNTVMIENLKF

F*

>CcSYP131

MNDLLGG

SFELPRDQSSRGGDIELGAQMNS

GELGLENFFKKVQDIDKEYEKLEKLLKKLQGAHEESKSVTKAPAMKAI

KKRMEKDVDEVGKISRFIKSKIEELDRENLTSRQKPGCGKGTGVDRSRT

ATTLGLKKKFKDKMGEFQVLRENIHQEYREVVERRVYTVTGKRADE

ETIDQLIETGDSEQIFQKAIQEQGRGQIMDTLAEIQERHDAV

RDLERKLLELQQIFLDMAVLVDAQGDMLDNIESQVSSAVDH

VQSGNTALQKAKKLQKNSRKWMCIAIIILLIIVA

IIVVAVIKPWSSNKGA

>AtSYP132

MNDLLKG

SFELPRGQSSREGDVELGEQQG

GDQGLEDFFKKVQVIDKQYDKLDKLLKKLQASHEESKSVTKAPAMKAI

KKTMEKDVDEVGSIARFIKGKLEELDRENLANRQKPGCAKGSGVDRSRT

ATTLSLKKKLKDKMAEFQVLRENIQQEYRDVVDRRVYTVTGERADE

DTIDELIETGNSEQIFQKAIQEQGRGQVMDTLAEIQERHDAV

RDLEKKLLDLQQIFLDMAVLVDAQGEMLDNIESQVSSAVDH

VQSGNTALQRAKSLQKNSRKWMCIAIIILLIVVA

VIVVGVLKPWKNKSA

>AtSYP131

MNDLLKG

SLEFSRDRSNRSDIESGHGPGNS

GDLGLSGFFKKVQEIEKQYEKLDKHLNKLQGAHEETKAVTKAPAMKSI

KQRMERDVDEVGRISRFIKGKIEELDRENLENRTKPGCGKGTGVDRTRT

ATTIAVKKKFKDKISEFQTLRQNIQQEYREVVERRVFTVTGQRADE

EAIDRLIETGDSEQIFQKAIREQGRGQIMDTLAEIQERHDAV

RDLEKKLLDLQQVFLDMAVLVDAQGEMLDNIENMVSSAVDH

VQSGNNQLTKAVKSQKSSRKWMCIAILILLIIII

ITVISVLKPWTQKNGA

>CpSYP131

MNDLLKD

SFEIPRGHSSRSGDIEMGGNAPSNS

GDLGLEDFFKKVQEIEKQNEKLDKLLKKLQNAHEESKAVTKAPAMKAI

KQRMEKDVDEVGKIARSMKGKIEELDRENLSNRQKPGCGKGSGVDRSRT

ATTLSLKKKLKDKMAEFQILRETIHQEYREVVERRVFTVTGTQADE

ETIDRLIETGDSEQIFQKAIQEQGRGQIMDTLAEIQERHDAV

RDLEKKLLELQQIFLDMAVLVDAQGEMLDNIESHVSSAVDH

VQSGNTALQKAKKLQKSSRKWMCIAIIILLIIVA

IIVVGVLKPWTSNKGA*

>MeSYP132

MNDLLSE

SFEIPRGQSSRAGDVEMGMSS

GDLGLESFFKKVQEIENQNEKLDKLLKKLQDAHEESKAVTKAPAMKAI

KKRMEKDVDEVGKIARSVKSKVEELDKENLANRQKPGCGKGTGVDRSRT

STTMALKKKLKDKMAEFQSLRETIHQEYREVVERRVFTVTGTRADE

ETIDRLIETGDSEQIFQKAIHEQGRGQVMDTLAEIQERHDAV

RDLEKKLLDLQQIFLDMAVLVDAQGEMLDNIESQVSSAVDH

VQSGNTALQKAKKLQRNSRKWMCIAIIILLLIVV

IIVVAVIKPWSNNKGA

>MeSYP131

MNDLLSE

SFEIPRGQGSRGGDIEMGMTS

TETGLENFFKKVQEIEKQNEKLDKLLKKLQDANEESKAVTKAPAMKAI

KQRMEKDVDEVGKIARSLKSKIEELDKENLANRQKPGCGKGTGVDRSRT

STTMALKKKLKDKMAEFQNLRETIHQEYREVVERRVFTVTGTRADE

ETIDRLIETGDSEQIFQNAIHEQGRGQIMDTLAEIQERHDAV

RDLERKLLDLQQVFLDMAVLVDAQGEMLDNIESQVSSAVDH

VQSGNTALQRAKSLQKSSRKWMCIAIIILLIIVA

IIVVAVIKPWSSNKGA

>VvSYP131

MNDLLSE

SFEIPRGQASREGDIELGERALQNS

GELGLENFFKKVQEIEKQNDKLNVQLKKLQDAHEESKAVTKAAAMKAI

KKRMEKDVDEVGKIARSIKAKVEELDKENLANRQKPGCGKGTGVDRSRT

ATTVALKKKFKDKMAEFQVLRESIHQEYREVVERRVFTVTGTRADE

ETIDRLIETGDSEQIFQKAIQEQGRGQIMDTLAEIQERHDAV

REVERKLLDLQQIFLDMAVLVDAQGDMLDNIESQVSSAVDH

VQSGNTALQRAKKLQRSSRKWMCIAIIILLIIVV

IIVVAVLKPWSKNGA

>PtSYP131

MNDLLSE

SFEIPRGQGSRGGDIEMGMNS

ADLGLESFFKKVQEIEKQNEKLDKLLKKLQDAHEESKAVTKAPAMKGI

KQRMEKDVDEVGKIARSIKSKLEELDKENLSNRQKPGCGKGTGVDRSRT

STTIALKKKLKDKMAEFQTLRENIHQEYREVVERRVFTVTGTRADE

ETIDTLIETGDSEQIFQKAIQEQGRGQITDTLAEIQERHDAV

RDLERKLLDLQQIFLDMAVLVDAQGDMLDNIESQVSNAVDH

VQSGNVALQKAKKLQRNSRKWMCIAIIILLIIVA

IIVVTVLKPWNNNKGA

>SlSYP132

MNDLLND

DDNFDAPRHQSNRNGDVEMGIQIPMNS

GELGLDDFFKKVQQIEKQYGRLNELLQKLQDAHEESKAVTKASAMKAI

KQRMEKDVDEVGKIARVIKSKIEELDKENLANRNKPGCGKGSAVDRSRT

ATTVSLKKKFKDKMAEFQTLRENIHHEYREVVERRVFTVTGNRADE

ETIDRLIETGDSEQIFQKAIQQQGRGQIMGTLAEIQERHDAV

RELERKLLELQQIFLDIAVLVDAQGDMLDNIESQVSTAVDH

VQSGTTALQKAKKLQKNSRKWMCFAIMILLIIVA

IIVVGVLKPWQSNKGA

>SlSYP131

MNDLLNDG

LDAFEAPRHQNDRSEDIEMGIQRPIDS

GELGLDDFFKQVQQIEKQHEKLEKLLMKLQDAHEESKAVTKATAMKAI

KQRMEKDVDEVSKVARFIKSKIEGLDKENLSNRSKPGCGKGSAVDRSRT

ATTVSLKKKLKDKMSEFQTLRENIHNEYREVVERRVYTVTGNRADE

ETIDRLIETGDSEQIFQKAIREQGRGQIMDTLAEIQERHDAV

RELERKLLELQQIFLDMAVLVDAQGDMLDNIESQVSAAVDH

VQSGNTALQKAKSLQRNSRKWMCIAIIILLIIVA

VIVVGVLKPWNSNKGA*

>SiSYP131

MNNLLSD

SFELPRRDSSRDADIEMGMHQADAS

DNLKDFLKKVDAIESLIAKLTNLLTKLQTANEESKAVTKASAMKAI

KQRMEKDIDEVGKFARQAKTKVDELEKDNLSNRQKPGCGKGSAVDRSRE

QTTGAVKKKLKERMDDFQALREAIRQEYREVVERRVFTVTGNRPDE

ETIDDMIETGKSEQIFKDAIQQGRGQILDTVAEIQERHDAV

RDLERKLLELQQIFMDMAVLVEAQGDMINNIETHVSNATNH

IQQGVSALQNAKKLQKNSRKWMCYAIIILLVIVV

IIVVAVIQPWKKGA*

>BdSYP131

MNNLLTD

SFELPRRDSSRDGDIEMGMHQADAS

DNLKGFLKKVDGIEGLIAKLTNLLTKLQTANEESKAVTKASAMKAI

KQRMEKDIDEVGKIARTAKTKVDELEKDNLSNRQKPGCGKGSAVDRSRE

QTTGAVKKKLKERMDDFQVLRESIRQEYREVVERRVFTVTGNRPDE

ETIDDLIETGRSEQIFKDAVQQQGRGQVLDTVAEIQERHDAV

RDLERKLLELQQIFLDMAVLVEAQGDMINHIETHVSNATNH

IQQGVGALQNAKKLQKNSRKWMCYAIILLLVIVA

IIILAVIQPWKK

>OsSYP131

MNNLLTD

SFELPRGGSSRDGDIEMGMQADPS

DNLKGFLKKVDAIESLIAKLTNLLHKLQTANEESKAVTKARDMKAI

KQRMEKDIDEVGKIARMAKTKVDELEKDNLSNRQKPGCGKGSAVDRSRE

QTTGAVKKKLKERMDDFQVLREAIRQEYRDVVERRVFTVTGSRPDE

ETVDNLIETGRSEQIFQEAIQQQGRGQILDTVAEIQERHDAV

RDLERKLLELQQIFMDMAVLVDAQGDMINNIETHVSNATNH

IQQGVSALQNAKKLQKNSRKWMCYAIILLLIIVV

IIVVAVIQPWKKGA

>AcSYP131

MNDLLTD

SFEIPRGQASRGGDIEMGTNS

VDMGLDRFLKQIDEIDRQIEKLNKLLKKLQDANEESKSVTKAAAMKAI

KERMEKDVDEVGKIALAVKTKIEQIDRDNLTNRQKPGYGKGTAVDRTRT

ARTVAVKKKLKDKMSEFQTLRQNIHQEYREVVERRVYTVTGTRADE

ETIDRLIETGDGEQIFQKAIQEQGRGQILDTVAEIQERHDAV

RDLEKKLLELQQVFMDMAVLVEAQGDMLDNIESQVTSAVDH

VQSGNTALQKAKKLQRNSRKWMCIAIIILLIIVA

IIVVAVIQPWKSNKGA*

>MtSYP132b

MNDLLTD

SFVSEANHGQPSRQGDIEMGLQDQRSSS

DMGMEAFNKQIQDADKQIEKVSALLQKLKEANEESKAVTKASAMKAI

KKRMEKDIDEVGKIAHGVKAKIEAINRENLNNRQKPGCEKGTGIDRARM

NMTNSLTKKFRDLMTEFQTLRQRIQDEYREVVERRVITVTGTRPDD

ETIDHLIETGNSEQIFQQAILEAGRGQVVSTVEEIQERHDAV

KEIEKKLLDLHQIYLDMAVLVEAQGEILDNIESQVNNAVDH

VQRGTTALQSAKKLQKNSRKWMCIAIIILLIIVA

IIVVGVLKPWKSS

>MtSYP132a

MNDLLTD

SFVSEANHGQPSRQGDIEMGLQDQRSSS

DMGMEAFNKQIQDADKQIEKVSALLQKLKEANEESKAVTKASAMKAI

KKRMEKDIDEVGKIAHGVKAKIEAINRENLNNRQKPGCEKGTGIDRARM

NMTNSLTKKFRDLMTEFQTLRQRIQDEYREVVERRVITVTGTRPDD

ETIDHLIETGNSEQIFQQAILEAGRGQVVSTVEEIQERHDAV

KEIEKKLLDLHQIYLDMAVLVEAQGEILDNIESQVANATDH

VKSGNDALHTAKSLQKKSRKCMMIAIILVLLIAI

FIVLGVVKPWKK

>CcSYP132a

MNDLLTD

SFVSDARGQPSRDNDIEMGTRVPRSSS

DMGMEGFNKQIQEIEKQVDKLSALLKKLKDANEESKSVTKASEMKAI

KKRMEKDVDEVGKIARNVKAKIEAINRENLANRQKPGCEKGTSVDRSRM

NVTNALTKRFKELMTEFQTLRQRIQEEYREVVERRVITVTGTRPDE

ETIDHLIETGNSEQIFQKAIQEQGRGQVLNTLEEIQERHDAV

KEIEKKLLDLQQIYMDMAVLVEAQGEILDNIESQVTHAVDH

VQLGTDALRIAKRLQRRSRKCMMIAIILLLVIAI

IIVLAVVKPWN

>CcSYP132b

MNDLLTD

SFVSDARGQPSRDNDIEMGTRVPRSSS

DMGMEGFNKQIQEIEKQVDKLSALLKKLKDANEESKSVTKASEMKAI

KKRMEKDVDEVGKIARNVKAKIEAINRENLANRQKPGCEKGTSVDRSRM

NVTNALTKRFKELMTEFQTLRQRIQEEYREVVERRVITVTGTRPDE

ETIDHLIETGNSEQIFQKAIQEQGRGQVLNTLEEIQERHDAV

KEIEKKLLDLQQIYMDMAVLVEAQGEILDNIESQVSNAVTN

VQSGTTALQNAKKHQKSSRKWMCIAIIILLIIVA

VIVVGVIKPWKSGNS

>MeSYP133a

MNDLLTD

SFVGDAKRPPSNNDIEMGMQLPRSNS

DMGMEAFNKQIQDVEKQVDKVSGLLKNLKEANEESKSVTKASSMKAI

KKRMEKDVDEVGKVARAVKAKLQAINKDNLANRQKPGCEKGTGVDRARM

NVTNALSKKFRDLMTEFQTLRQKIQDEYREVVERRVITVTGTRPDE

ETIDHLIETGNSEQIFQKAIQEMGRGQVLNTLEEIQERHDAV

KEIEKKLLDLHQIYLDMAVLVEAQGEILDNIESQVANAVDH

VQSGTDALKTAKSLQRNSRKCMMIGIILLLIIAI

IIVLFVVKIWKK

>MeSYP133b

MNDLLTD

SFVGDAKRPPSNNDIEMGMQLPRSNS

DMGMEAFNKQIQDVEKQVDKVSGLLKNLKEANEESKSVTKASSMKAI

KKRMEKDVDEVGKVARAVKAKLQAINKDNLANRQKPGCEKGTGVDRARM

NVTNALSKKFRDLMTEFQTLRQKIQDEYREVVERRVITVTGTRPDE

ETIDHLIETGNSEQIFQKAIQEMGRGQVLNTLEEIQERHDAV

KEIEKKLLDLHQIYLDMAVLVEAQGEILDNIESQVANAVNH

VQSGTTALQNAKKLQKNSRKWMCIAIIILLIIIA

VIVVGVVKPWKSSKGA

>PtSYP132a

MNDLLTS

SFIGDSKGHHPIDGDIEMGIRGSRSNN

PDMGMEAFNKKIQEVGKQLDKLLKNLKKEANEDSKSVTKASAMKAI

KKRMEKDVDEVGKIARNVKERIVAINKDNLDSRQKPGCEKGTGVDRARM

NVTNAITKRFRDLMTEFQTLRQKIQDEYRELVERRVITVTGTRPDE

KTIDHLIETGNSEQIFQKAIQEQGRGEVLNTLEEIQERHDAV

KEIEKKLLELKEIFGDLAVLVDAQGEILDNIENQVTNAVDH

VHNGTDALRTAKNLQKKSRKCMMIAIILVLIIAI

IIVLSILKPWKKN

>PtSYP132b

MNDLLTS

SFIGDSKGHHPIDGDIEMGIRGSRSNN

PDMGMEAFNKKIQEVGKQLDKLLKNLKKEANEDSKSVTKASAMKAI

KKRMEKDVDEVGKIARNVKERIVAINKDNLDSRQKPGCEKGTGVDRARM

NVTNAITKRFRDLMTEFQTLRQKIQDEYRELVERRVITVTGTRPDE

KTIDHLIETGNSEQIFQKAIQEQGRGEVLNTLEEIQERHDAV

KEIEKKLLELKEIFGDLAVLVDAQGEILDNIENQLPALWQDRVFLGIALMKMLLRLQMQ

SLMSQSGTTAFQTAKKLQKNSRKWMCIAIIILLLVVA

IIVD

>TcSYP132a

MNDLLTD

SFVGDAQGHGDIEMGRQVPGSNS

DMGMEAFNKQIQEVEKQVEKLSVLLRKLKDDNEESKSVTKASAMKAI

KKRMEKDIDEVGKIARNVKARLEAINKDNLANRQKPGCEKGTSIDRSRM

NVTNALAIKFKDLMIEFQTLRQKIQDEYREVVERRVITVTGTRPDE

QTIDRLIETGNSEQIFQKAIQEQGRGQVLNTMEEIQERHDAV

MGIEKKLLDLQQIYLDMAVLVEAQGEILDNIESQVTNAVDH

VQSGTDALRTAKSLQKKSRKCMMISIILLLIIAI

IVVLSILKPWKK*

>TcSYP132b

MNDLLTD

SFVGDAQGHGDIEMGRQVPGSNS

DMGMEAFNKQIQEVEKQVEKLSVLLRKLKDDNEESKSVTKASAMKAI

KKRMEKDIDEVGKIARNVKARLEAINKDNLANRQKPGCEKGTSIDRSRM

NVTNALAIKFKDLMIEFQTLRQKIQDEYREVVERRVITVTGTRPDE

QTIDRLIETGNSEQIFQKAIQEQGRGQVLNTMEEIQERHDAV

MGIEKKLLDLQQIYLDMAVLVEAQGEILDNIESQVSTAVTN

VQSGTIALQNAKKRQKSTRKWTCIAIIILLIIVA

VIVVGVLKPWKSS

>CpSYP132a

MNDLLTD

SFVGDAKGMPPRDNDIEMGRQDPRSNS

DIGMEAFNKQTQEIEKQVDKLSGLLKKLKRKGTSYGNFFLQDANEESKSVTKALAMKAI

KKRMEKDIDEVGKIARSVKAKLEAVNKDNLDNRRKPGCEKGTSIDRSRM

NVTNALTKKFKDIMTEFQTLRQRIQDEYREVVERRVITVTGSRPDDEIMEMVVILMVQLF

QTIDNLIETGNSEQIFQKAIQEQGRGQRPLLQVLNTLEEIQERHDAV

RDIEKKLLDLQQIYLDMAVLVEAQGEILDNIENQVMSAVDH

VNMGTDALHHAKSLQKKSRKCMIISIIILLIIAI

IIVLSILKPWKK

>CpSYP132b

MNDLLTD

SFVGDAKGMPPRDNDIEMGRQDPRSNS

DIGMEAFNKQTQEIEKQVDKLSGLLKKLKRKGTSYGNFFLQDANEESKSVTKALAMKAI

KKRMEKDIDEVGKIARSVKAKLEAVNKDNLDNRRKPGCEKGTSIDRSRM

NVTNALTKKFKDIMTEFQTLRQRIQDEYREVVERRVITVTGSRPDDEIMEMVVILMVQLF

QTIDNLIETGNSEQIFQKAIQEQGRGQRPLLQVLNTLEEIQERHDAV

RDIEKKLLDLQQIYLDMAVLVEAQGEILDNIENQVATAVTQ

VQSGTTALQNAKKLQKNSRKWMCIAIIILLLIVA

VVVVGVLKPWQNKNGA

>VvSYP132a

MNDLLTD

SFVGDAKVQPPKERDIEMGTRVLRTNS

DLGLEAFNKQIQEVEKQVDKLTFLLKKLKDANEESKSVTKASAMKEI

KKRLEKDVDEVGKIAQNVKAKLEAINRDNLANRKKPGCEKGTGVDRSRK

NMTNALTKKFKDLMIDFQALRQKMQDEYREVVERRVMTVTGTRPDE

ETIDNLIETGNSEQIFQKAIQEMGRGQVLATLEEIQERHDTV

KEIEKKLLDLHQIYLDMAVLVEAQGDLLNNIENQVTNAVDH

VQWGTDALRTAKSLQKKSRKCMMFAIILILIIAA

IITLSVLKPWK

>CsSYP132a

MNDLLTD

SFVSNAKVESSREIDLEKGTRVLRSNS

DMGMEAFNKQIQDVEVQVDKLAGLLIKLKEANEESKSVTKASEMKAI

KKRMEKDIDEVGKIARSVKGKLEAVNKDNLTNRQRPGFEKGTAIDRARM

NVTNALTKKFKDLMIEFQTLRQRIQAEYREVVERRVITVTGTRPDE

TTIDHLIETGNSEQIFKNAFEQMGRGQVISTVEEIQERHDAV

KEIERKLSELHQIYLDMAVLVEAQSEILDNIENQVTNAVDH

VRTGTDALQTAKSLQKRSRKCMMIGIILLLVIAI

IIILSVLKPWKK

>SlSYP133b

MNDLLAD

SSFIAGKDNASKESDIEMGNRFTRSQS

DSGIDSFNKQIQEIEKQVDRLSGLLKTLKDANEETKSVTKASAMKAI

RKRMEKDIDEVGKIARNVKAKIEATNKENLANLQKPGCGKGTSVDRSRT

NMTNSLTKKFRDVMTEFQTLRQRIDNEYREVVERRVITVTGTRPDE

ETINNLIETGNSEQIFQNAIQGMGRGQVLSTVEEIQERHDAV

KEIERKLLDLHQIYLDMAVLVEAQGDLLDNIETQVRYAVDH

VNMGTDALQTAKSLQKKSRKCMMIAIILLLIIAA

IIVLSVIKPWKK*

>SlSYP133a

MNDLLAD

SSFIAGKDNASKESDIEMGNRFTRSQS

DSGIDSFNKQIQEIEKQVDRLSGLLKTLKDANEETKSVTKASAMKAI

RKRMEKDIDEVGKIARNVKAKIEATNKENLANLQKPGCGKGTSVDRSRT

NMTNSLTKKFRDVMTEFQTLRQRIDNEYREVVERRVITVTGTRPDE

ETINNLIETGNSEQIFQNAIQGMGRGQVLSTVEEIQERHDAV

KEIERKLLDLHQIYLDMAVLVEAQGDLLDNIETQVTNTIDY

VQSGAIALQTAKKLQKRSRKCMFISIIVFLIIVG

IIVLPFIIKSQPWKSNKGA

>AcSYP132

MNDLLT

MEGKQPRRDRSIEMGAQFPSGNS

DSSMEAFFKQVRELEKQMDKLSQLLQKLQVANEESKTVTKASTMKAI

KQRMAKDIDEVGKIARKIKTKLDEIDKDNAANRQKPGCGKGTGVDRSRV

AMTNALKKKLKERMTDFQILRKNIQDEYRDVVERRVFTVTGNRPDD

ETIDQLIETGNSEQIFHKAIQEMGRGQVLDTVEEIQERHDAV

KEIEKKLLDLHQVFLDMAVLVDAQGDLMDNIEIQVTNAVDH

VQSGNTALTTAKNLQKKSRKCMMIAITILLIIAI

IVVLSIVKPWSKGKT*

>SiSYP133

MNNLLTD

SFERDEKPERERDVEMGNRNPKDKT

DYGLKDFYEEVKEIEMLLDKMSKIVHKLQEANEESKSVTKASAMKAI

KGRMEKDIDEVGKIARSVKVKLEQMDRNNLENRKKPGCGKGTSVDRSRM

SMTITLKKKMKERMNDFQNLRQTIQEEYREVVERRIFTVTGTKPSE

EVIDRLIETGSSEQIFERAIQGTGRGQILATIEEIQERHDAV

MEIEKRLLELQQIFADMAALVDAQGEILDNIENQVQNAVNH

VVTGTEALRTAKSLQKKSRKCMLIAIILLLVIAV

IVVLSILKPWAK*

>OsSYP133

MNNLLTN

SFESERDIEMGYQNSKNKS

DYGLEDFFQEVQEIETLLDKMSNINHKLQEANEESKSVTKASEMKAI

NKRMEKDINEVGKITRTIKVKLEEMDRNNLENRKKQGCEKGTGVDRSR

IALKNKLKERMKNFQNLRQIIQDEYRQGVARMVFTVTGEQPSD

QVIDHLIETGSSEQIFEKAIQGIGRGQIIATVKEIHERHDVV

MEIEKKLLELQQIFADMATLVDAQGETLNDIENQVQNAVDH

IQRGTGELRTAKRLQKKSRKCMFIAIIILLVIAA

IVVLSILKPWANSVCKVSRLLG

>BdSYP132

MNNLLTD

SFEMDEKPPKERDIEMGRRNSKNKS

DYGLEDFYEEVKDIEMLLDKMSNIVQKLQEANEESKSVTKASAMKAI

KGRMEKDIDEVGKIARNIKMKLEQMDRNNLNNRKKPDCGKGTGVDRSRM

SMTIALKKKLKERMKDFQNLRQTIQQEYREVVERRIFTVTGTKPSE

EVVDNLIETGSSEQIFEKAIQGIGRGQIMATVEEIQERHDVV

MDIEKKLLELQQIFTDMAALVDAQGEILDNIESQVQNAVNH

VQTGTEALRSAKNLQKKSRKCMMIAIIMLLVIAG

IIVLSILKPWAK

>SiSYP134

MRNLLTD

SFELGKRELAPGNVDIELGLQGDLTSS

AQPGFEGFFEQVKDIENLLSTLTKLLKDLQNSNEESKVVTKASAMKEV

KKRMEKDVNEVTKVARLAKSKVEELNKDNAANRQKPGFGKGSGVDRSRT

TTTVALTKRLRERILEFQTLREEIQKEYRGVVERRVFTVTGERADE

ETIDKLIETGDGEQIFQRAIQEQGRGRVLDTLQEIQERHDTV

KEIEKKLLDLHQIFLDLAVLVEAQGEMLDNIETQVTGAAEH

IQTGTNLLQKAKKLQKNTRKWTCIAIIILLIIVL

VIILSLKPWSWGK*

>OsSYP134

MRNLLTD

SFELSKVDQAPANVDIELGLQGGMSSS

AQPGFEGFFEQVREIEKLHETLTKLLKDLQNSNEESKIVTKASAMKEI

KKRMEKDVNEVTKTARLAKSKVEKLNKDNAANREKPGFGKGSGVDRSRT

TTTVSLTKRLRERISEFQTLREAIQKEYRDVVERRVFTVTGERADE

ETIDKLIETGDSEQIFQRAIQEQGRGRVLDTLQEIQERHDAV

KEIEQKLLELQQIFLDMSVLVEAQGEILDNIESQVSGAAEH

IQTGTNLLQKARFLQKNTRKWTCIGIVILLIIIL

IVVLSLKPWSK

>BdSYP133

MRNLLSD

SFEISKGEQAPGNVDIELGLQGDTASS

AQPGFKGFFEQVREIEKLLETLTKLLKDLQNSNEESKVVTKPATMKEI

KKRMEKDVNEVTKVARLAKSKLEQLNKENLANREKPGFGKGSGVDRSRT

TTTVALTKRLRERISEFQALREVIQTEYKEVVERRVFTVTGERADE

ETIDRLIETGDSEQIFQRAIQEQGRGRVLDTLQEIQERHDIV

KEIEKKLLELQQVFLDLSVLIEAQGDTLNNIEAQVTNAADH

LQTGTNHLQKAKVLLKSKRKWTCIAIILLLIVVL

IVVLSLKPWSWGNKNA

>SmSYP131

MNDLLAGS

SPQARISGDSNGDLEAGV

ANTGMEDFLQEVGHITTSMGQIRKNLHKLQEAHEESKTVTKAAAMKAL

KKRMEADVDEVSKAARKIKVKLEALDKSNISNRKKPGCGEGSSIDRQRM

SMTATLKKKLKDLMGEFQDLRQKFQDEYREVVERRIFTVTGKRPEE

NMIEQLIETGDSESIFQKAIQEQGRGQILDTVAEIQERHDAV

KDMEKKLLELHQIFLDMAVLVEAQGEMLDNIETQVSKAVDH

VQAGTSALKRTKQLQRSKRKWMCIAIIILLIVIA

VIVVGTSMRNVSFANSI*

>SmSYP132

MNDLLG

DAHEDTKTVTKSDVMKAK

KNEMEAQVDVVTKITQDIKHKIESLDKANILNRKKPNCGEGSSTDRTRM

SMTVTLKKKLKDVMQEFQNLRQKLQDEYREVVERRIFTVTGQKPDE

ETVDKLIETGDSETIFQKAVQEQGRGQILDTIAEIQERHDAV

RDIEKKLLDLHQIFMDMAVLVEAQGEMLDDIENQVSKAVDH

VQTGTAALEKVKKLQRGTRKCMCVGIILLLVIVI

IIVVAVIQPWK*

>CmSYP1

MPNDLLAQV

KGKNALYDGEDDNEIEDIEEQVAPPPTD

AEREMQEFFKKVELVKTDLAEVKELQKEILSMHEKGKTIVKSKEMQKH

RELMQEKIDAVNKLAHACKAKIEALDKDNDAAKKKKGQQAGSASERTRT

TITAGLKKKLKDHMQEFSELRTRIQSEYREVVERRVYTVTGTHATD

EEIDKMIETGDSENIFQKAILEQGRGRVLDTLAEIQERHRAV

KDLEQSLLELHQIFLDMAVLVEAQGEMLDNIEKQVARSVDY

VKGGTEALQDAKQLQKNTRKWMCCAIMIMLIVAL

VIVLAVVRPWKYLQ

>Utricularia_gibba(v4.1_unmasked)_Name:_Scf00110.g8922.t1_Type:_CDS_Feature_Location:_(Chr:_Scf00110_complement(5928..6836))_Genomic_Location:_59286836

MNDLFSH

SFKRYHEDDLEAAGNAGRTE

GVNLDKFFDEVESVKQDMSDVEKLYRRLQESNEQCKTAHSAKTMKDV

RSRMDSDVTQILKRVKIIKGKLEALEKSNATNRKVPGCGPGSSADRTRS

SVVSGLGKKLKDLMDGFQSLRCKMNAEYKETVARRYFTITGEKPDD

ETVENMISSGESETFLQKAIQEQGRGQILDTISEIQERHDAV

KEIEKNLIELHQIFLDMAALVEAQGQQLKDIESHVAHASSF

VRRGTEHLQEAREYQKSSRKWSYIAFLLVIALIVLL

TFPIWSHLLLTAII

>Utricularia_gibba(v4.1_unmasked)_Name:_Scf00043.g4899.t1_Type:_CDS_Feature_Location:_(Chr:_Scf00043_complement(52250..53185))_Genomic_Location:_5225053185

MNDLFAAP

SRDSHVIEFANVAAGS

GGANLNRFFDDVESVKEELAELESLYARLQNFHEQSKTLHNAAAIKDL

RSRVDADVTTALKKAKVIKLRLEALDRSNAAQRSFPGCGPGSSSDRTRT

SVVNGLRKKIQDSMARFNELRQKMSDEYRETVQRRYYTVTGENPDE

RIVDRLIESGESEKFLQKAIQQQGRGQVMDTIMEIQERADAV

KELEKNLVELHQVFLDMAVLVESQGEQLDDIHLNVTRANSY

VRGAADQLATARKHQKSTRKWTCYGIILLLIIAL

IVILSLRPWTWNQNQNQNQNQNRNSTP

>Utricularia_gibba(v4.1_unmasked)_Name:_Scf00031.g3844.t1_Type:_CDS_Feature_Location:_(Chr:_Scf00031_join(16734..1680817150..18061))_Genomic_Location:_1673418061

MKWNAADSLVDTIGAPVLVLRRLPLLGFLGIEAKMNDLMTK

SFMSYVDLKKQAMKDLESNLPDAEMGQITSS

EQKNLSIFFEEVDAIRAEIEELRYLLIDLQELNESTKSAQSPKILRGL

RDRINSDMVMVLRKAKVVKTRLESLEKSNTDNQTGHKEGSYVDRTRS

TVTNGLRTKLKATMDDFQSLREKVVSDHKEVLKRRYYAATGEEASD

ETIEKIALEGGTAGVFEGKREIVEENVERHEAV

KAIQKSLVELQQLFLDMAVMVETQGEHMNDIEMNVMNAGSY

VKEGTKELDRAQKLKKQRTWACWIGVLVLLVVLVCLIA

LLF

>Utricularia_gibba(v4.1_unmasked)_Name:_Scf01249.g24298.t1_Type:_CDS_Feature_Location:_(Chr:_Scf01249_join(9079..95179633..10084))_Genomic_Location:_907910084

MNDLLSES

SGRARNSHPVEGINNSAG

ADDFDPFFEDVESVKEGLADLEALYLRLQRSNEQSKTIHNGADVKRL

RSRMEADVTTALKKAKAIKLRLEALNRSNAALQKLPGRGPGSSFYRTRT

SVVNGLMKKLHDSMNRFNDLRLKAGLEYRETVQRRYYAVTGEYPDE

KTLDHLIKTGESETFLRKAIGEQGRGQVMDAIMEVRERHDAI

KEMERNLMELHQVFLDMVVLVESQGEVIDDIERQVNSATAF

VEGGAEELEQARKLQKSTRKWACFGIAFLLLIVL

VIVLSLHPWRK

>Utricularia_gibba(v4.1_unmasked)_Name:_Scf00096.g8289.t1_Type:_CDS_Feature_Location:_(Chr:_Scf00096_143824..144789)_Genomic_Location:_143824144789

MGSRNPGFEYVLEILRNFTFDCITVKMNDLMTK

SFLNYVDLKKQVMKDIEAQAPYMEMGDLASA

DQKNLSMFFIEVDAIRADMEELKYLLLDLQELNVSAKSAQSSKILRGL

RDRINSDMVMVLRKGKMIKTRLEALETSNIRNRSDYKEGSYIDRTRT

AVTNGLRVKLKVTMDDFQTLREKVVSDHKEVLRKRYYAVTGEQPSE

ETIEKIASDGGLVGGVFEGKMEMVEEQQERHAAV

KAIQKSLVELQQIFLDIAVIVENQDERMNDIETNVVNAGVY

VNQGTKELDRANRLKKQRIWACWIGVAVLVIVLVCLIA

ILF

>Utricularia_gibba(v4.1_unmasked)_Name:_Scf00028.g3616.t1_Type:_CDS_Feature_Location:_(Chr:_Scf00028_join(226867..226920227083..227139227310..227358227555..227643227705..227778227870..227912227991..227

MGTERSS

ADLGLGDFFKKVQEIEKQYEKVNSILRKLQEAHEESKAVTKAAAMKAI

KKHMEKDVDEVGKIARSIKLKIEELDRENLSNRQKPGCGKGSGVDRSRT

AATVALKKKFKDKMAEFQTLRENIHQEYREVIERRVFAVTGSRADE

AAIDKLIETGDSEQIFQKAIQEQGRGQIVETVAEIQERHDAV

RDLEKKLLDLQQIFLDMAVLVDAQGDMLNNIESQVSSAVEH

VQSGNTALQKAKTLQKSSRKWMCIGIIILLIIIV

IVVVGVLKPWQSRNGA

>Utricularia_gibba(v4.1_unmasked)_Name:_Scf00702.g20965.t1_Type:_CDS_Feature_Location:_(Chr:_Scf00702_join(18714..1909319161..1960019686..19690))_Genomic_Location:_1871419690

MEDDIELGNDPTTN

AVDEVDFLREDINAVEELFARLLESSDHIKNAQSTSAMKQI

RALVNDYLDHLLKTAKQIDKKFGVLLLNHRRAGSADQ

QDFSGIADDINQIMRNFQGLRAQMEADQRQTIEARYFAITGEKGTS

EAIDHLIATHAPGTHLHGALLDQGRGAILEAVAEVEERRDLM

MELRRSLINLHQILLGISRPVAAEQPGNAAAMEQQHPPKSLPP

QAGAKTGGNGGLNDYEKETRKQAYIAIGIALIINIS

VVILLLNGEARVENG

>Utricularia_gibba(v4.1_unmasked)_Name:_Scf00564.g19507.t1_Type:_CDS_Feature_Location:_(Chr:_Scf00564_join(23201..2321823614..2371823883..2393924147..2419524442..2453024601..2467424745..2478724864..242

MNDLLSD

SFISSRDQGNTAGDIEMGTERPT

ADLGLEDFFKKVQEVEKQYEKVNSILRKLQEAHEESKAVTKAAAMKAI

KKRMEKDVDEVGKIARSIKLKIEELDRENLSNRQKPGCGKGSGVDRSRT

ATTVALKKKFKDKMAEFQTLRENIHQEYREVIERRVFTVTGSRADE

ETIDKLIETGDSEQIFQKAIQEQGRGQIVDTVAEIQERHDAV

RDLEKKLLDLQQIFLDMAVLVDAQGDMLNNIESQVSSAVDH

VQSGNTALQRAKSLQKSSRKWMCIGIIILLIIIV

IIVVGVLRPWQSRNGA

>Utricularia_gibba(v4.1_unmasked)_Name:_Scf00078.g7253.t1_Type:_CDS_Feature_Location:_(Chr:_Scf00078_complement(join(139121..139471139563..139751)))_Genomic_Location:_139121139751

MRSKCLLIDLQELNESKTSAESPRTLRGV

RDGINSDIMMVLRKAKGVKSRLESLEKRP

STTGVKIALEGGG

TVGRIFPEGKGDVLEENLERHEAV

KAIQKILVELQQLFLDTDVMVGTQGEGIRMRHEMNVMNAGSY

FNQGRKELHRAHKLKNQRTVGSEFCPWLSCRSASSPYC

SNQT

>Pd_103720597

MNNLLND

SFELHRGEASRDGDIEMGQQVPMNS

ADLGLEGFFKQVQVIEKQIEKLSKLSKKLQTANEEANAVTKASDMKGI

KQRMQKDIDEVGKIARVAKAKLEELDRDNLTNRQKPGCGKGSSVDRSRS

ATTVALKKKLKERMSEFQTLRETIQQEYREVVERRVFTVTGNRPDE

ETIDRLIETGDSEQIFQKAIHEHGRGQVMDTLAEIQERHDTV

RELERKLLELQQIFLDMAVLVEAQGDLLDNIESQVSSAVDH

VQSGTVALQKAKKLQKNSRKWMCIAIIILLLIVV

IIVVAVIKPWSKNK

>Pd_103719700

MNNLLTD

SFELPRGEASRDGDIELGTQVPMNS

GELGLEGFFKQVQDIEKQIEKLSKLLKKLQDANEESKAVTKAAAMKAI

KQRMEKDIDEVGKIARLAKSKLEELDRENLANRQKPGCGKGSGVDRSRT

ATTIALKKKLKEMMSDFQALREKIQQEYREVVERRVFTVTGNRADE

ETIDRMIETGNSEQIFQRAIQEQGRGQVLDTLAEIQERHDTV

KEIERKLLDLQQIFLDMAVLVEAQGDMLDNIETQVSSAVDH

VQSGSTALQKARRLQKNSRKCMCIAMLILLIIGV

IIVLAVIKPWSKN

>Pd_103716820

MNDLLSD

SFVMSSDQSPKEKDMELGIQVPKGKS

DYGMEDFFKQVKDVEKLMEKLSKHLQKLQEAKEESRYVTKASAMKAI

KQQMEKYVDEVGKIVRNIKTRLEEIDKDNLINRSKPGCEKGTGVDRSRM

TMTAALKKKLKDKMNDFQNLRQTIQDEYREVVERRIFAVTGARPTE

EMIDHLIETGNSEQIFQKAIQEMGRGQVCDWHVEEIQERHDAV

KEIEKKLLELHQIFMDMAVLVEAQGEILDNIECQVSNAVNH

VQSGTEALHTAKNLQKNSRKCMMIAIIILLVIAA

IIVLSILKPWKK

>Pd_103723513

MTT

VNYSFDKLCVQEIEKQIETLSKLLKKLQDANEEFKAVTKAAAMKAI

KKRMEKDIDEVGKIARLAKSKLEELDRENLANRQKPGCGKGSGVDRSRT

ATTIALKKRLKERMSDFQALRETIQQEYREVVERRAFTVTCNHADE

ETIDRWIETGNSEQIFQRAIQEQGRG

QIFLDMAVLVEAQGDILDNIESQVSSAIDH

VQSGATALQRAKKLQKNSRKWICIAILILLIIVI

IIIVTVIKPWSKS

>Pd_103720705

MNDLMTK

SFLSYVDLKKEALKDLESGAEEDGIEMTAAA

ADENLRRFFEEAGLVREEMGSIRDLLARLQDANEESKSLHKPEALRCL

RDRINADIVQVLKKARAIRARLEAMDRSNAANRRLSGCREGTPVDRTRT

SVTNGLRKKLKELMMDFQGLRQRIMAEYNETVERRYFTVTGEVPDE

DMIEKIISNGESEELLKKAIMEHGRGKVLATLHEIQDRHDAA

KEMERSLLELHQVFLDMAVLVEAQGEQMDSIEHHVTSASHY

VKDGTKELRCAKEYQRSSRKWLCIGVLLLLILIL

VIVIPIATSFSKS

>Pd_103700157

MNDLFSTS

SFKKYTDLKQQVHLDEMESGTMGGMGEAGK

ESVNLDKFFEDVENVKDDLRGLGKLYRRLQEANEESKTVHNAKTMKDL

RARMDADIDQVLRRAKAVKGKLEALDRANAAHRKLPGCGPGTSADRTRT

SVVSGLGKKLKDLMDDFQGLRTRIAAEYKETVGRRYYTVTGEHADE

QTIETLISSGESETFLQKAIQEQGRGQIMDAISEIQERHDAV

KEIEKSLLDLHQVFLDMAALVEAQGHQLNDIESHVAHANSF

VRRGTVELETARDYQKSSRKWYCMAILLGALLVALL

LLPFLTNVMNLI

>Pd_103705725

MNSLFSG

SWRREADGSSGDVQMSRNSSASAA

GGANLDRFFEDVEAIKDELKEVERIHHRLHESNEASKTLHAASAVRDL

RARMDGDVATALKKAKLIKLRLESLDRANAANRSLPGCGPGTSTDRTRT

SVVAGLRKKLKDSMEGFVELRKRVAADYRESVERRYYTVTGEKPDE

QTVETLVATGEGERFLQRAIEEQGRGRVLDVIAEIQERHGAA

ADLERSLLELQQVFLDMSVLVAAQGEHLDDIESQVGRAHSF

VRRGTDQLQTARKHQKNTRKWTCIAIILLLIIILI

IVLPIVLNKK

>Pd_103708663

MNDLMTK

SFMSYVELKKQALKDLEADPVLDLEAGGLSCA

EEENLSLFFEEIGAIQSAMEDISSLLLDLQLLHEETKSAHSAKVLRGL

RDRMDSDVVSVLRKAKIIKARLEALDQSNIANRGLSARFSEGSPVDRTRI

SVANGLRTKLREMMNGFQSLRERILAEHKEALKRKYFNATGEAASE

EVIEKMLSGGSQVGLLDKKGEVDLEIMERQKAV

SDMQRSLMQLHQVFLDMAIMVEVQGEQLNDIEENVVRAKDY

IGGGTDSLISANALRKRNRKCTYCVWALVLLVLIVCLIP

ILTGF

>Ma02_p19160.1_Syntaxin132

MNNLLSE

SFELSRGEPSRDRDIELGLQQTMNV

AEQGLEKFFKQVEEIEKLIEKLSKLSANLQAANEKSKSVTKASDMKAI

KQHMQKEIDEVGKIARLAKSNLEELDRDNLASRQKPGCGKGSSVDRSRT

ATTVALKKKLKERMSEFQTLRETIHQEYREVVERRIFTVTGNRADE

ETIDRLIETGNSEQIFQKAIQEQGRGQVMDTLAEIQERHNTV

KDLERKLLELQQIFLDMAVLVDAQGEMLDNIESQVSSAVDH

VQSGTVALQKAKKLQKNSRKWMCIAIIILLLIVV

IIVVAVIKPWSK

>Ma07_p17290.2_Putative_syntaxin131

MNNLLSD

SFDLPRDEPPRDGDIESGLQHPKSA

AEQGLEGFFKQVEEIEKLIEKLAKLSKNLQAANEKSKAVTKASDMKAI

KQHMQKDIDEVGKIARLAKSKLEELDRDNLASRQKPGCGKGSGVDRSRT

ATTIALKKKLKERMSEFQTLRETIKQEYREVVERRVFTVTGNRADE

ETIDHLIETGNSEQIFQKAIQEQGRGQVMDTLAEIHERHNTV

KDLERKLLELQQIFVDMAVLVDAQGEILDNIESQVSSAVDH

VQSGTVALQKAKKLQKNSRKWMCIAIIILLLIVV

IIVVAVIKPWSK

>Ma02_p03500.1_Putative_syntaxin131

MNNLLTD

SFELPRGEASGYGDVEMGRQISTNS

AEMGFEGFFTQVQDIEKQIEKLSKLLQNLQDANEESKTVIKAAAMKEI

KHRMENDIDEVLRVARTTKTKLEELDRDNLANRQKPGCGKGSSTDRSRT

ATTVALKKKLKEKMSQFQTLRETIQKEYREVIERRVFTVTGTHANE

ETIDQLIETGKGDQIFAKAIQEQGRGQVLDTLAEIQERHDAV

RDIEKKLLELQQIFLDMAVLIEAQGEMLDNIEDQVNRAGVF

VSSGVNALSKAKILQKNSRKCM

>Ma09_p25290.1_Putative_syntaxin131

MSTSQSPKVRDVELGSLHSDNTS

DYGMEDFFKQVTGIEKMMEKISNHLKKLQEANENSKSATKASSMKKI

RQQMEKDVDEVGKIAHMIKTKLQEIDRDNLANRKKPGCEKGTGVDRSRM

ALTAALKKKSKDRMNDFQNLRKTIQDENREVVERAVFTVTGTLPTD

EMIDRLIATGNNEQIFQRQFKKWHVFQIIDIIEEIQERNDAV

FMDMAVLVEAQEEILDNIESQVATAMNH

VQSGNNELLTAKSLSKRSRKCMFIAIVILLAIAA

VIVLSILKPWK

>Ma10_p08930.1_Putative_syntaxin131

MNNLMTE

SFERVVGSYPGEGDIGSGRYSTDT

RDMGMEGFFKEVGEIDKQIERITGFFNKLQATNEESQLVTKASEMKAI

KQRMEKDVEEVKKIALKIKTMLEQLDKNNMINRQKPGCGKGTAVDRSRM

GMTVALKKKLKERMTQFQTLRQNIHEEYREVVERRVFTVTGTRPDE

EMVDQLIETGNSELIFEKAIQGQGRGQVVDTLAEIQERRDTM

LELEKKLLDLQQMFLDMSVLVDAQGDMLDDIEAQVTKSVDH

IENATATLQAAKKTQKNTRKYM

>Ma06_p06430.1_Syntaxin124

MNDLLSTT

SFKQYTNLKHQVQMDQMEAGMSGPGADVG

ANLERFFEEVENVKNDLRGLESLYRRLQEANEESKTAHSAKTMKAV

RARMDADIGQVLRQAKAVKAKLEALDQANAQHRNIPGCGPGSSADRTRT

SVVSGLGKKLKDLMDDFQGLRTRIAAEYKETVGRRYYTVTGTHADD

DTIETLISSGASEKFMQKAIQEQGRGQVMDTISEIQERHDAV

KEIERSLLDLHQVFLDMAALVEAQGHQLNDIESHVAHASSF

VRRGTVELETAREYQKSSRK

>Ma10_p28240.1_Syntaxin124

MNDLLSTT

SFKKYTNLKQQVQMDQMEAGMSEMGAVAG

VNLEQFFEEVESVKKDLRGLEGLYRRLQDANEESKTVHNAKAMKEV

RGRMDSDIGLVLRSAKAVKAKLEALDRSNAQHRNVPGCGPGSSADRTRT

SVVSGLGNKLKDLMDSFQGLRTRIAEEYKETVGRRYYTVTGTHADE

ETIETLISSGASETFVQKAIQEQGRGDVMDTISEIQERHDAV

KEIEKSLLDLHQVFLDMAALVEAQGHQLNDIESHVAHASSF

VRRGTVELETAREYQKSSRKWVCIG

>Ma01_p17220.1_Syntaxinrelated_protein_KNOLLE

MNDLMTK

SFYSYADLKKEALKDLESGAADDVAIEMAAGD

ADGHLRHFFEEAGLAKEEMAAIRDLLARLQAANEESKSLHQPEALRGL

RDRINADIVRVLKTARGIRGRLEAMDRSNAANRRLSGCREGTPVDRTRT

AVTNGLRIKLKELMMEFQALRQRMMAEYRETVERRYFTVTGEAPEE

EVIERIIANGESEGMVKKAMLEHGRGKVLATVHEIQDRHDAA

KEVERSLLELHQVFLDMAVMVEAQGEQMDDIEHHVASAAHY

VKDGTRELKCAKEYQRSSRKW

>Ma08_p02910.1_Syntaxin121

MNNLFSSA

SWRQDVESGGGRGSELQMAA

GGANLDSFFEDVETLKNDLREVERLHRSLHEANEEGKSLHEASAVRAL

RARMDADVALALKKAKFIKLRLESLDRANAANRAVPGCGPGTTTDRTRT

SVVAGLRKKLRDSMDVFAELRSKVASDYRETVERRYYTVTGDVPDE

ATVDELVATGEGERFLQRAIEEQGRGRVLDVVAEIREWHGAV

AELERSLLELQQVFMDMAVLVEAQGQQLDDIESNVGRAQSF

VRHGTEQLGAARQHQKSTRKWTC

>Ma06_p32840.1_Syntaxin121

NLDRFFEDVEAIKDELREVERLHRSLHEANEAGKTLHEASAVREL

RGRMDADVALALKKAKLIKLRLESLDRANAANRAVPGCGPGSSTDRTRS

SVVAGLRKKLRDSMEAFAELRRRIAAEYRETVGRRYYTVTGESPDE

ATVDALVATGEGERFLQRAIEEQGRGRVLDVVAEIQERHGAV

AELERSLLELQQVFMDMAVLVEAQGQQLDDIESNVGRAQSF

VRHGTDNLTTARVYQKNTRKWTCIA

>Ma10_p29500.1_Syntaxin112

MNDLMTK

SFMSYVELKKQALKDLEAGADDPPKVEAAAGGLTRM

EEENLARFFSEIGGIQSEMDDVSSLLVDLRFLNEESKSAHSAKLLRGL

RDRMDADVVAVLRKAKAIKVRLESLDRSNAANRGVAACFTEGSPVDRTRV

SVTNGLRTKLRETMNGFQLLRERIVSDHREGLKRRYLNATGEAATE

ETIDKMLSGASQVGLLDNRGEVDLEVLERQKAV

SDIQRSLMQLHQVFLDMAVMMEGQEDQLNDIEENVARAKDY

ISGGTDRLVSANAMRKRNKKCACLV

>Nn_NNU_021112RA

MNDLFSS

SFKKYTDLKQQNQFDDMEAGGDASGE

GVNLDKFFEDVENVKEDMRNVEKLYKRLQESNEESKTVHNAKTMKDL

RSRMDKDVEQVLKQVKVIKGKLEALERSNVANRSVPGCGPGSSADRTRT

SVVAGLGKKLKDMMDDFQGFRAKMTAEYKETVERRYFTITGEKADE

ETIENLISSGESETFLQKAIQDQGRGQILDTISEIQERHDAV

KEIEKNLIELHQVFLDMAALVEAQGQQLNDIESHVAHANSF

VRRGTEQLTVAREYQKSSRKWTCIAINLGILLVILL

ILPLLPTIITLMK

>Nn_NNU_002644RA

MNDLFSG

SFSRFRSNDVDHEPPYSGGGGGGGAVQMGT

VGVNLDKFFEDVEGIKDELKEEERLHQRLQDSHEESKTLHNARSIKDL

RARMDADVALALKKAKMIKVRLEALERANAANRSIPGCGPGSSSDRTRT

SVVSGLRKKLKDQMESFNTLRERISTEYKETVERRYFTVTGEKPDD

RTVDALISTGQSENLFQKVIQEQGRGTVMDIVSEIQERHDAV

KEIEKNLRELHQVFLDMAVLVQTQGEQLDDIESQVARANSF

IRGGTEKLVTARKLQKNTRKWACFGITLLLIVVL

IIILSLKPWNWGGSSGGGSPAAPVTSAPSTPTPPSPPQQ

>Nn_NNU_017127RA

MNDLMTK

SFLSYVDLKKEAMKDLEAGNLDMEMAATATQNQ

IDHNLAQFLEEAEMVKKEIGFIRDVLGRLQQANEESKCLHKPEALKAL

RIRTNADIVTVLKKARSIKSQLEDMDRANAANRRLSGYREGTPVDRTRT

SVTNGLRKKLKELMMDFQELRQRMMAEYKETVGRRYFTVTGEYPEE

EVIEKIISSDGGTGGEELLQRAVQEHGRGKVLETVLEIQDRHDAA

QEIEKSLLELHQVFLDMAVMVEAQGEQMDDIQHHVMNAAEY

VKGGTRDLRTAKNYQKSSRKWMCVGLILLLILIL

LVVIPIATSFSHS

>Nn_NNU_011499RA

MNDLFSG

SFSGLRSNNVDLEPPYSRRGASGGDVQMRT

VGVNLDKFFEDVEGIKDELKEVEALHRRLQDSHEESMTLHNARSIKDL

RARMDADVALVLKKAKMVKIRLEALDRANAAHRSTPGCGPGSSSDRTRT

SVVSGLRKKLKDQMENFNTLREKIATEYKETVEHRYFTVTGEKPDK

RTVDALISTGQSENLFQKMIQEQGRGTVMDIVSEIQERQDAV

KEIEKNLKELHQVFLDMAVLVQSQGEQLDDIESQVARATSF

IRGGTEQLQVARTLQKNTRKWACFGITILLIIII

IIVIIVVPKKKSSPAP

>Nn_NNU_011720RA

MKGSLFRTIVSPSIINLKTLQPTKEKRKSPEPSLHRGGWFLQILGNMNDLMTK

SFLSYVELKKQALEDLEAGPDTEMGRLGPT

DEENFAQFFEEVGAIKDEMEVINNLLHDLQCLNEETKSTHSAKVLRGL

RDRMDSDMVTVLRKAKIIKARLEALDRSNVANRSISAAYGEGSPVDRTRV

SVTNGLRIKLRDMMNNFQSLRETIVSDHKEGLKRRYFHATGEVPTE

EMIDKMILAGGQVEVFGGRTELNLENQERQEAV

KQIQRSLTELHQVFLDMAVLVEAQGEQMDDIEENLARAGNF

ISGGTDRLVSANKMKKGKWVCWVWLLVLLILLVCFLV

AVT

>Dca51659.1

MNNLLSD

SFELQRGQSSRDGDPEKGLNNS

GELGLQSFSKKVQELEKQYEKINNLLRKLQGAHEESKAVTKAAAMKSI

KQRMEKDVDEVQKIARLIKSKIEDLDRDNLSSLQKPGCGKGTAIERTRT

TQTVQLKKKLRDKMAEFQTLRENVHQEYREVVERRVYTVTGQRADE

ETIDQLIETGNSEQIFQRAIQEQGRGQVMDTLAEIQERHDAV

RDVERKLLELQQIFLDMAVLIDAQGDVLDNIESQVQNAVDH

VNTGNTALQRAKSLQKNSRKWMCFAIIILLIIVA

VIVVGVLKPWQNKNGA

>Dca20532.1

MNNLLSD

SFDLPRGQSTRENDLETGLNNS

GELGLQIFSKKVQEIEKQYEKINVLLRKLQGAHEESKSVTKAAAMKSI

KQRMEKDVDEVQKIARLIKSKIEDLDKDNSSSLQKPGCGKGTAIERTRT

MQTVQLKKKLRDKMGEFQKLRENIQEEYREVVERRVYTVTGQRADE

ETVDRLIETGNSEQIFQKAIQEQGRGQVMDTLAEIQERHDAV

RDVERKLLELQQIFMDMAVLVDAQGDILDNIELQVQNAVDH

VNSGNTALQKAKSLQKNSRKWMCIAILILLIIVA

VIVVGVLKPWQNKNGA

>Dca53324.1

MNDLFSS

SFKKYANLKEQREMDDLESGKET

INLDKFFEDVEKVKEDMNQVVALYRRLQDANEECKTVTNAKAVKDL

RARMDGDVEQVLKRAKIIKGKLEALDKSNVAHRRLPGCGPGSSADRTRT

SVVSGLGKKLKDMMDDFQGLRAKMSSEYKETVERRYFTVTGERPDE

ETIEKLISSGESESFLQKAIQSQGRGQIMDTVQEIQERHDAI

KEIEKNLYELHQVFLDMAALVEAQGHQLNDIASNVNRASSF

VNQGTGELVQAKVHQKNSRKWTCYAIILAIIIVIII

LFPILFSTILRPGP

>Dca11983.1

MNDLFSS

SFKKYANLKEQREMDDLESGKET

INLDKFFEDVEKVKEDMNQVVALYRRLQDANEECKTVTNAKAVKDL

RARMDGDVEQVLKRAKIIKGKLEALDKSNVAHRRLPGCGPGSSADRTRT

SVVSGLGKKLKDMMDDFQGLRAKMSSEYKETVERRYFTVTGERPDE

ETIEKLISSGESESFLQKAIQSQGRGQIMDTVQEIQERHDAI

KEIEKNLYELHQVFLDMAALVEAQGHQLNDIASNVNRASSF

VNQGTGELVQAKVHQKNSRKWTCYAIILAIIIVIII

LFPILFSTILRPGP

>Dca27582.1

MNDLFSS

SFKKYSDLKQGFSDIEAGGAE

GMNLDGFFEDVEKVKEDMKEVEKLYKKLQDLNEESKVAHNAKTMKDL

RARMDSDVSLVLKKVKVIKGKLEALEKSNATSRNVPGCGPGSSTDRTRI

GVVSGLGKKLKDMMEEFQGLRAKMQAEYKETMERRYFTITGEKADE

TMIENLIASGESENFLQKAIQDQGRGQIMDTISELQERHDSV

KEIEKNLIELHQVFLDMAALVEAQGQQLNNIESHVAHASSF

VRRGTEQLVEAREYQKSSRKCGMSPGSRPAGDRDSDGANTGVADEVWVLDGG

NAGAFGEAGVGVSDGGKADEP

>Dca493.1

MNDLMTK

SFMSYVDLKKAVAKDDDIDLEMGIGGS

TDPKMEAFLEEAEKVKAEIASISDILTTLEAANEESKSLHKPDALKAL

RGRINADIVSVLKKTKAIKARLEVMDQSTAANCRLSVELGTPVDRTRK

CVTNGLRKKLKELMMDFQTLRQKMMSEYKETVGRCMYTVTGEHPDD

EAIEKIISNGVGEGEILSHAIQEHGRGKVLETVMEIQGRYDAA

KEIERSLLELHQLFLDMSVMVEAQGEQMDNIEHHVVNTRHY

VQDGSKELKQAKVYQRGSRKCMCIGIIALLILIF

VVVIPIIASVSHS

>Bv_Bv_43750_ircr.t1

MNDLFSS

SFKRYANLKDQAQLDDLELGNQTG

TANLDKFFIDVDGVKGDMTHVEKLYKRLQDANEECKIATNAKAVRDL

RARMDGDVEQVLKRAKIIKGKLEGLERSNAAHRNLPGCGSGSSADRTRT

SVVSGLGLKLKDMMDKFQTLRTKMSLEYKETVERRYFTITGEKAEE

ETIEQLISSGESESFLQKAIQDQGRGQIMDTVKEIQERHDAI

KEIEKNLLELHQVFLDMAALVEAQGQQLNDIATNVNRASSF

VSQGVNELVVAKDHQKNSRKWTCIAIILGIVLVIVI

LFPILYSTLLVPR

>Bv_Bv8u_204830_jeew.t1

MNDLFSN

SFRKYSEFEQQGFKNDVESGTE

GMNLDKFFEDVEKVKEDMKEVEKIYKRLQDQNEESKVVHNAKTMKEL

RARMDSDVSLVLKKVKIVKAKLEALDKSNVQARNVPGCGPGSSADRTRT

SVVSGLGKKLKDMMDNFQALRAKMQSEYKETMERRYFTITGEKADE

TMIENLIASGESENFLQKAIQDQGRGQILDTISELQERHDAV

KEIEKNLIELHQVFLDMAALVEAQGQQLNNIESHVAHASSF

VRRGTEELVEARVQQKNSRKWYCYAILAGIVLIFVLVFP

LFINIILPHLVR

>Bv_Bv5_104810_jypk.t1

MNNLLST

SFSQHGKNNSGDIEMGLAAD

AGANLEKFFEDVEKINEDLKEIEELQRKLRAGHEESKTAHNAGVVKAL

RSRMDQDVSQTLKKAKLIKDRLQALDKSNAASRSLPECGPGSSTDRTRT

SVVGGLGKNLKDKMEEFQELREKINGEYRETIERRYYTVTGENPDE

KTVDLLISTGESETFLQKAIQQQGRGQILDTIAEIQERHDAV

RDIERNLKELHQIFLDIAVLVQHQGEQLDDIEANVDRANSY

VQRGTNQLHEARKHQKNTRKWTCFAIILLLIIIL

IIVLSLRPWAR

>Bv_02910_yrnu.t1

MNDLMTK

SFLSYVDLKKAAAKDVEKDLELGMSVAN

LDPKMEAFLEEAEKVKAEIGSIEDTLKTLEASNEESKSLHKPEALKAL

RSRISVDIVTVLKKAKAIKARLEQMDRATAENRRLSADMGTPIDRTRT

CVINGLRKKLKELMMEFQALRQRMMSEYKETVGRCYYTVTGENPDD

DVIEKIISNGAGEGEILSHAIQEHGRGKVLETVIEIQGRHDAA

KEIERSLLELHQVFLDMAVMVEAQGEQMDNIEHHVINASNY

VQDGNKNLKKAKDYQRGSRKCMCIGIIALLVIIL

LIVIPIIASLSHS

>Bv_Bv6_125600_spfd.t1

MNDLMTN

SFLNYVDLKKQIELDHETELSEAETWQQLNPI

DQDNLSNFFKEIESIKSQMEEVSNLLHDLQSLHQESKTTHSTKLLRGF

RDRMDSDTIAILRKVKSLKSRLESLDKSNITNRRLSNSFREGSTVDRTRV

AVTNGLRIKFKDMISDFTGLREKIMVDHKDELRRKFFNATGEVPSE

EIVDKLLSGSVKIEVFEGRIGGADMEEKERYEVV

KDIQRSLNKLHQVFLDMAVLIEQQGEQINDIEQNVVNAGEF

ITGGTNNLYYAKQMKKRNRAWFFGIWGLAIIILLVCLIC

ILALK

>StHe62GB1_9442

FSS

SFKRYGGDLETGSGGTARTE

GVNLDKFFDDVESVKRDMSDVDRLYRRLQELNEESKTVHSAATVKDL

RSRMDADVAQVLKRVKTIKSKLEALDRSNAAHRRVPGCGPGSSADRTRT

SVVSGLGKKLKDTMDDFQGLRARMNAEYKETVERRYFTVTGEKPDD

ELVESMISSGESESFLQKAVEEQGKGRILDTITEIQERHDAV

KEIERNLVELHQIFLDMAALVEAQGQQLNDIESHVAHASSF

VRRGTEQLQEAREYQKGSRKWYCVAVLLVIVLVALL

TFPVW

>StHe62GB1_52084

MNDLFSG

SFSRFRNHGGDQSPPPPDSHSIEMTT

GGVNLDRFFADVESVKDDLRDLESLHAQLLSAHDQSKTLHDARSVRDL

RSRMDADVSAALKKAKLVKVRLEALDRSNAANRSVPGCGPGSSSDRTRT

SVVNGLRKKLQESMSRFADLRQRMGSEYRETVQRRYFTVTGENPDE

AVLDRLIETGESETFMQKAIQEQGRGRVMDTIAEIQERHDAV

REIER?PEGAAPGVPGHGGAGG

EPGGA

>StHe62GB1_8571

RCRVGPGSGAGHGP

IGPKPDCVLGGSRASEERDGVHQGDPGPIGSLPRGGQGPAQAPSPKID

QGRVNSDILSVLKRARAIRAKLEEMDQANASNRRLSGCKEGTPVDRARF

AVTNGLRKKLKEVMMDFQGLRQRMMSEYKETVGRRYYTITGEYPDE

EVLEKIISNGGEDLLSKAVQEHGRGKVVETVVEIQDRHDAA

KEIERSLLELHQIFLDMAVMVEAQGEQMDDIEHHVMNAAHY

IAQGTNNLKVAKDHQKSSRRCMCLGIVLLLIVVL

VVLIPVLVSFSHS

>StHe62GB1_69192

MDRGAFDG

SFKIYVEDPRKPGPDIETGRMGS

GPYGMEMFAKEVDNVKEDMKSMDKLYGQVHESNEDIKVAQNAAAMREL

RDEVNTDLDHVIKLAKQINKKYDGLVRANAAQRRVAGSGPGSSDDNARA

KMISELGDNIKQTMRRFQGLRVQMETDHRQLIESRYFAITREKATA

EAIDNLIANEVPESPLHHAMQDHGRGPVMDAVAEIQERRGAM

MEARRTLMSLHQILLGIATPVAAVAAEGQAKAP

>Pinustaeda_2A_I10_VO_L_2676_T_4/6_C_0.429

MNDLLN

KSFSNYGAFKQEPMRDLEAGPEVEMANNG

NDKNLGQFFEQVGSIKRDMEAMQELFGNLQRANEESKTVHKAQAMKGL

RDRMDKDVVEVLKKAKLVKGKLEALDRANVASRRLLGCEEGTPTDRTRT

SITNSLRKKLKDSMGEFQILRQKIMGEYRDTVQRRLYNITGEYADE

DTLEKIVSTGESENILQKAIQEQGRGRILETIHEIQERHDAA

KEIEKSLLELHQIFLDMAVLVEAQGEQLNDIEHHVIHAANY

VDHGTKQLKGAKKYQRNTRKWMCIGIILLLILVLIIV

IPIATSFKKS*

>Pinustaeda_3855_T_2/7C_0.600

MNDLFS

RSFKNYANLKEDSRKDLEAGYDVEMVGIN

EEKNLGQFFGEVEDVKNDMESVEGLSRKLQASHEESKTIHNAKTIKNL

RDRMDKDVEAVLKEAKLIKGKLETLDKANIESRRLPGCGEGSPTDRTRT

SIVNGLRKKLKDMMAEFQLLRQKIMAEHKETIGRRYYAVTGEQADE

ETIENMISTGESETFLRKAIQEQGRGQILDTIHEIQERHDAA

MEIERNLLDLHQIFLDMSVLVEAQGEQLDDIEHHVTHASSF

VSRGTQQLQTAKTHQRNTRKWTCIGIIVLLVIILVIV

VPIAISLSKRKALVNSIWNRAYQWLRMKEAHGNLFPDWYLLNPS

VSELR*

>Pinustaeda_6A_all_VO_L_443_T_707/721_C_0.010

MLSSDGAMNTISLEIHAVT

AQPDLRSFKNYANLKEDSRKDLEAGYDVEMVGIN

EEKNLGQFFEEVEDVKNDMESVEGLSRKLQASHEESKTIHNAKTIKNL

RDRMDKDVEAVLKEAKLIKGKLETLDKANIESRRLPGCGEGSPTDRTRT

SIVNGLRKTLKDLMAEFQILRQRIVAEYKETIGRRYYAVTGEQADE

ETIENMISTGESETFLRKAIQEQGRGQILDTIHEIRERHDAA

MEIERNLLDLHQIFLDMSVFVEAQGEQLDDIEHHVTHASSF

VNRGTQQLQTAKTHQRNTRKWTCIGIIVLLVIILVIV

VPIATSLKPK*

>Pinustaeda_6A_I27_VO_L_2371_T_7/23_C_0.306

MADLFS

RSHKDYASLEEVSKRDLEAGYDVDREGIY

TEESLSQFFEEVEDVKKDMESVKGLLRKLLDSNEESKTIHNAKTIKNL

RDTMDKDVEAVLKKAKLIKGKLEALDKANIASRRLPGCGQGSPTDRTRT

SIVNGLRKKLKDMMAEFQLLRQKIMAEHKETIGRRYYAVTGEQADE

ETIENMISTGESETFLRKAIQEQGRGQILDTIHEIRERHDAA

MEIERNLLDLHQIFLDMSVFVEAQGEQLDDIEHHVTHASSF

VNRGTQQLQTAKTHQRNTRKWTSIAIILILIIITVIV

VPIAISLSKRKALVNSIWNRAYQWLRMKEAHGNLFPDLVFT*

>Pinustaeda_6A_all_VO_L_443_T_684/721_C_0.009

MLSSDGAMNTISLEIHAVT

AQPDLRSFKNYANLKEDSRKDLEAGYDVEMVGIN

EEKNLGQFFEEVEDVKNDMESVEGLSRKLQASHEESKTIHNAKTIKNL

RDTMDKDVEAVLKKAKLIKGKLEALDKANIASRRLPGCGQGSPTDRTRT

SIVNGLRKKLKDMMAEFQLLRQKIMAEHKETIGRRYYAVTGEQADE

ETIENMISTGESETFLRKAIQEQGRGQILDTIHEIRERHDAA

MEIERNLLDLHQIFLDMSVFVETQGDQLGDIEQHVSHASSY

VNHGTQQLEAVKTHQRNTRKWTSIAIILILIIITVIV

VPIAISLSKRKALVNSIWNRAYQWLRMKEAHGNLFPDLVFT*

>Gb_Gb15769_c0_seq1

MNDLLT

RSFTSYMDLKRAALKDLEAGPEMEMVDLD

TDENLGEFFEEVGAVKTDMETVKELLAKLQKANEETKTVHKAQAMKAL

RDRMDRDVVTVLKKARVIKGKLEALDKANMANRKMPGCEEGTPTDRTRT

SITNGLRKKLKDLMGDFQGLRQKVVGEYRETVERRYYTITGEHANE

ETIETIISTGQSETFLQKAIQEQGRGQILDTIHEIQERHDAA

KEIERNLLELHQVFLDMAVLVEAQGEQLNDIEHHVIHAATY

VDHGTKQLRSAKNYQRNSRKWMCIGVILLLILILIIV

IPIATSLRNA

>Gb_Gb9530_c0_seq1

MNDLLTD

SFELPRMGASKDEGDLEAGTSVAGNAA

SDLGMADFFKHVKDIEKLMEKLSKQLQKLQEAHEESKSVTKASAMKAI

KQRMEKDVDEVGKVARNIKQKLESLDADNVANRKKPGCEKDTSVDRSRM

AMTATLKKKLKERMSDFQTLRQMIQDEYREVVERRVFTVTGTKADE

ETIERLIETGDSEQIFQKAIQEQGRGQIMDTLAEIQERHDAV

KDIEKKLLELHQIFLDMAVLVESQGDMLDNIETQVSNAVEH

VQTGTVALQKAKSLQRSSRKWMCIAIIILLIIVV

IIVVAVIKPWKSNKG

>Gb5794_c0_seq1

MNDLMR

RSFSTYADLKQEAMMDLEAGADMEMADIS

ADKNLGQFFQEVGNIKFDMEAIKVLLGRLQNANEESKAIHKAQAMKAL

RDRMDKDVLEVLKKAKVVKGKLEALDKANMANRRLVGCEEGTPTDRTRT

SITNSLRNKLKDSMGEFQILRQKIMGEYRDTIERRYYTITGEHADE

DTIEKIISTGESETIFQKAIQEQGRGRILETIREIQERHDAA

KEIERNLLELHQVFLDM
