# Supplementary material for: SNARE Complexity in Arbuscular Mycorrhizal Symbiosis
Source: Front Plant Sci. 2020 Apr 3;11:354. doi: 10.3389/fpls.2020.00354 (PMC7145992; doi:10.3389/fpls.2020.00354)
Supplement: Supplementary file 2 [file Data_Sheet_2.docx]

>AtVAMP721_AT1G04750

MAQQSLIYSFVARGTVILVEFTDFKGNFTSIA

AQCLQKLPSSNNKFTYNCDGHTFNYLVEDGFT

YCVVAVDSAGRQIPMSFLERVKEDFNKRYGGGKAAT

AQANSLNKEFGSKLKEHMQYCMDHPDEISKLAKVKAQVSEVK

GVMMENIEKVLDRGEKIELLVDKTENLR

SQAQDFRTTGTQMRRKMWLQNMKIKLIVLAIIIALILIIVLSVCHGFK

C*

>AtVAMP726_AT1G04760

MGQQSLIYSFVARGTVILAEYTEFKGNFTSVA

AQCLQKLPSSNNKFTYNCDGHTFNYLADNGFT

YCVVVIESAGRQIPMAFLERVKEDFNKRYGGGKAST

AKANSLNKEFGSKLKEHMQYCADHPEEISKLSKVKAQVTEVK

GVMMENIEKVLDRGEKIELLVDKTENLR

SQAQDFRTQGTKMKRKLWFENMKIKLIVFGIIVALILIIILSVCHGFK

CT*

>AtVAMP725_AT2G32670

MGQQNLIYSFVARGTVILVEYTEFKGNFTAVA

AQCLQKLPSSNNKFTYNCDGHTFNYLVENGFT

YCVVAVESVGRQIPMAFLERVKEDFNKRYGGGKATT

AQANSLNREFGSKLKEHMQYCVDHPDEISKLAKVKAQVTEVK

GVMMENIEKVLDRGEKIELLVDKTENLR

SQAQDFRTQGTKIRRKMWFENMKIKLIVLGIIITLILIIILSVCGGFK

CT*

>AtVAMP723_AT2G33110

MAQQSLFYSFIARGTVILVEFTDFKGNFTSVA

AQYLENLPSSNNKFTYNCDGHTFNDLVENGFT

YCVVAVDSAGREIPMAFLERVKEDFYKRYGGEKAAT

DQANSLNKEFGSNLKEHMQYCMDHPDEISNLAKAKAQVSEVK

SLMMENIEKVLARGVICEMLGSSE

SQPQAFYIKRTQMKRKKWFQNMKIKLIVLAIIIALILIIILSVCGGFN

CGK*

>AtVAMP722_AT2G33120

MAQQSLIYSFVARGTVILVEFTDFKGNFTSIA

AQCLQKLPSSNNKFTYNCDGHTFNYLVENGFSESKYCSI

SYCVVAVDSAGRQIPMAFLERVKEDFNKRYGGGKAAT

AQANSLNKEFGSKLKEHMQYCMDHPDEISKLAKVKAQVSEVK

GVMMENIEKVLDRGEKIELLVDKTENLR

SQAQDFRTQGTQMRRKMWFQNMKIKLIVLAIIIALILIIILSICGGFN

CGK*

>Me_015780m.g

MGQQSLIYSFVARGTVILAEYTEFTGNFTSIA

AQCLQKLPATNNKFTYNCDGHTFNYLVDNGFT

YCVVAVESVGRQVPIAFLERVKEDFSNRYGGGKAAT

AVANSLNKEFGPKLKEQMQYCVDHPEEVSKLAKVKAQVSEVK

GVMMENIEKVLDRGEKIELLVDKTENLR

SQAQDFRQQGTKMRRKMWFQNMKIKLIVLGILIALILIIVLSVCKGFK

CN*

>Me_015786m.g

MGQQSLIYSFVARGTVILADFTEFTGNFTSIA

AQCLQKLPATNNKFTYNCDGHTFNYLVDNGFT

YCVVAVESVGRQVPIAFLERIKEEFTKKYGGGKAAT

AVANSLNKEFGPKLKEQMQYCVDHPEEVSKLAKVKAQVSEVK

GVMMENIEKVLDRGEKIELLVDKTENLR

SQAQDFRQQGTKMRRKMWLQNMKIKLIVLGILIALILIIVLSVCGGFK

CH*

>Me_015834m.g

MGQQTLIYSFVARGKVILAEYTEFKGNFTSVA

GQCLQKLPASNNRFTYNCDGHTFNYLVENGF

TYCVVAVESAGRQIPIAFLERIKEDFVKRYGGGKAKT

AQANGLKREFGPKLKDHMQYCVAHPEEINKLAKVKAQVSEVK

GVMMENIEKVLDRGEKIELLVDKTESLR

SQAQDFRQQGTKVKRKMWIENMKMKLIVFGIVVAMILIIFLSICHGFK

C*

>Me_015841m.g

MGQQSLIYSFVARGTVILAEYTEFTGNFTSIA

SQCLQKLPASNNKFTYNCDGHTFNYLVENGFT

YCVVAVESAGRQIPIAFLERVKEDFNKRYSGGKAAT

AQANSLNREFGSKLKEHMQYCVDHPEEVSKLAKVKAQVSEVK

GVMMENIEKVLDRGEKIELLVDKTENLR

SQAQDFRQQGTKMRRKMWLQNMKIKLIVLGIIIALILIIVLSICHGFK

C*

>Cc_10005852m.g

MGQQSLIYSFVARGTVILAEYTEFTGNFTSIA

SQCLQKLPATNNKFTYNCDGHTFNYLVENGFT

YCVVAVESAGRQIPIAFLERVKEDFNKRYGGGKAAT

AVANSLNKEFGSKLKEHMQYCVDHPEEISKLAKVKAQVSEVK

GVMMENIEKVLDRGEKIELLVDKTENLR

SQAQDFRQQGTKMRRKMWIQNMKIKLIVLGIIIALILIIVLSVCHGFK

C*

>Cs_140220

MGQQSLIYSFVARGTVILAEYTEFTGNFTSIA

SQCLHKLPATNNKFTYNCDGHTFNYLVDNGFT

YCVVAVETAGRQIPIAFLERVNEDFVKRYGGGKAMT

ATANSLNKEFGSKLKEHMKYCVEHPEEISKLAKVKAQVSEVK

GVMMENIEKVLDRGEKIELLVDKTENLR

SQAQDFRQQGTKMRRKMWYQNMKMKLIVLAIILALILIIILSVCGGFN

C*

>Cc_10022162m.g

MGQQSLIYSFVARGTVILAEYTEFTGNFTGIA

AQCLQKLPSSNNKFTYNCDGHTFNYLVDDGFT

YCVVAVESVGRQVPIAFLERVKEDFIKRYGGGKAAT

AVANGLNKEFGPKLKEHMQYCIDHPEEISKLAKVKAQVSEVK

GVMMDNIEKVLDRGEKIELLVDRTENLR

SQAQDFRQQGTQMRRKMWFQNMKIKLIVLGIIIALILIIVLSVCGGFN

C*

>Cs.256280

MGQQTLIYSFVARGTVILAEYTEFTGNFTTIA

SQCLQKLPASNNKFTYNCDGHTFNYLVENGFT

YCVVAIEAAGRQIPIAFLERVKEDFNKRYGGGKAAT

AVAHSLNKEFGSKLKEHMQYCLDHPEEISKLAKVKAQVSEVK

GVMMENIEKVLDRGEKIELLVDKTENLR

SQAQDFRTQGTKMRRKMWFQNMKIKLIVLGIIVALILIIILSVCHGFQ

C*

>Cs.266290

MAQKSLIYSFVARGTVILADYSDFSGNFTNIA

FQCLQRLPASNNRFTYTCDGHTFNYLLNNGFT

YCVVAVEAAGRQVPMACLERIKEDFDRRYGGGKASV

AVAKSLNKEFGPKMKGHMQYCVDHPEESSKLMQVKAQVSDVK

AVMIENIDKIMERGTKIEDLVDKTENLR

SQAKEFQTNGTQIKKKMWYQNMKIKLIVFSILIILALIIVLSICRGFN

C*

>Cp_59.108

MGQQSLIYSFVARGTVVLAEYTEFTGNFTSIA

SQCLQKLPASNNKFTYNCDGHTFNYLVENGFT

YCVVAVESAGRQIPIAFLERVKEDFNKRYGGGKAAT

AVANGLNKEFGPKLKEHMQYCVDHPEEISKLAKVKAQVSEVK

GVMMENIEKVLDRGEKIELLVDKTENLR

SQAQDFRTQGTKMRRKMWIQNMKIKLIVLGIIIALILIIVLSVCHGFK

C*

>Cp_8.171

MGQQSLIYSFVARGTVVLAEYTEFTGNFTSIA

AQCLQKLPASNNKFTYNCDGHTFNYLVDNGFT

YCVVAVESVGRQVPIAFLERIKEDFTKRYGGGKAAT

AAANSLNKEFGSKLKEHMQYCVDHPEEISRLAKVKAQVSEVK

GVMMENIEKVLDRGEKIELLVDKTENLR

SQAQDFRQQGTKMRRKMWFQNMKIKLIVLAILIALILIIVLSVCGGFK

C*

>Vv_01000524001

MVGQQSLIYSFVARGTVILAEYTEFTGNFTSIA

AQCLQKLPASNNKFTYNCDGHTFNYLVENGFT

YCVVAVESAGRQIPIAFLERVKDDFNKRYGGGKAAT

AVANGLNKEFGPKLKEHMQYCVDHPEEISKLAKVKAQVSEVK

GVMMENIEKVLDRGEKIELLVDKTENLR

SQAQDFRQQGTKMRRKMWMQNMKIKLIVLGIIIALILIIVLSICHGFK

C*

>Vv_01028579001

MGQQSLIYSFVARGTVILAEFTEFSGNFTSIA

AQCLQKLPATNNKFTYNCDGHTFNYLVEDGYT

YCVVAVEAVGRQIPIAFLERVKEDFTKRYGGGKAAT

AVANSLNKEFGPKLKEQMQYCVDHPEEISKLAKVKAQVSEVK

GVMMENIEKVLDRGEKIELLVDKTENLR

SQAQDFRQQGTKMRRKMWLQNMKIKLIVLGILIALILIIVLSICGGFN

C*

>MtVAMP721f_Medtr1g047720

MGQQTLIYSFVARGTMILAEYTEFSGNFSTIA

SQCLQKLPSSNNRFTYNCDGHTFNYLVDNGFT

YCVVAVESAGRQIPIAFLERVKEEFSKKYGGGKAAT

ASARSLNKEYGPKLKQQMQYCVDHPEEINKLAKVKAQVSEVK

GVMMENIEKVLDRGEKIEMLVDKTDNLR

SQAQDFRTQGTKMKRKMWIQNMKIKLIAVGIIFVIVLMIFMSICRGFS

CLH*

>MtVAMP721a_Medtr4g022570

MGQQSLIYSFVARGTVILAEYSDFTGNFTTIA

LQCLQKLPASNNRFTYNCDGHTFSFLVDNGFT

YCVVAVESVGRQIPIAFLERIKDDFNKRYGGGRATT

ATAKSLNKEFGPKLKEQMQYCVEHPEEVSKLAKVKAQVSEVK

GVMMENIDKVIDRGEKIEVLVDKTENLR

SQAQDFRQQGTQLRRKMWYQNMKIKLIVLAIIIALILIIVLSVCHGFS

C*

>MtVAMP721c_Medtr7g058640

MVQQSLIYSFVARGMVILAEHTNFTGNFVEIA

LQCLQRLPATNTKFTYNTDGHTFNYLAHDGFT

YCVVGVESFDRHIAMAFLDRIKEDFTKRYGGGKAAT

ATSKSLNKEFGPKLKEHMQYCVEHPEEVSKLAKVKAQVSQVQ

DVMLENIDQVLNRQVKIDVLMDKTDNLR

DQAQVFRREGGQLRRKMWFQNMKIKLIVLAIIIVIILIIVLLVT*

>MtVAMP721b_Medtr7g058900

MGQQSLIYSFVARGTVILAEYTEFTGNFTAVA

AQCLQKLPSSNNKFNYNCDDHTFNYLVDSGFT

YCVVAVESAGRQIPIAFLERIKEDFSKKYAGGKAEN

AAAKSLNKEFGSKLKEQMQYCVEHPEEISKLSKVQAQVSEVK

GVMMENIEKVLDRGEKIELLVDKTENLR

SQAQDFRQHGTKLRRKMWFQNMKIKLIVLGIIIALILIIVLSICGGFN

CSK*

>Fv_gene17527v1.0

MGQQSLIYSFVARGTVILAEFTEFTGNFTGIA

AQCLQKLPATNNKFTYNCDGHTFNYLVDNGFT

YCVVAVEALGRQVPIAFLERIKEDFTNRYGGGKAAT

AVANSLNKEFGSKLKEHMQYCVDHPEEISKLAKVKAQVSEVK

GVMMENIEKVLDRGEKIELLVDKTENLR

SQAQDFRQQGTQMRRKMWFQNMKIKLIVLGILIALILIIVLSVCNGFK

C*

>Pt_001G050400

MGQQTLIYSFVARGTVLLAEYTEFKGNFTGIA

AQCLEKLPASNNKFTYNCDGHTFNYLVENGFT

YCVVAVESAGRQIPIAFLERVKEDFNKRYSGGKAAT

AVAKSLNGEFGSKLKGHMQYCVDHPEEISKLAKVKAQVSEVK

GVMMENIEKVLDRGDKIELLVEKTENLR

SQAQDFRQQGTKMRRKMWIQNMKIKLIVLGIIIALILIIVLSICHGFN

C*

>Pt_002G240900

MGQQSLIYSFVARGTVILADYTDFSGNFAGIA

AQCLQKLPATNNKFTYSCDGHTFNYLVDNGFT

YCVVAVESVGRQIPIAYLERIKEDFTNRYGGGKAAI

AVANSLKKEFGPKLKEQMQFCMDHPEEVSKLAKVKAQVSEVK

GVMMENIEKVLDRGEKIELLVDKTENLR

SQAQVFRQQGGQIKRKMWWQNMKIKLIVLAILIVLILIIVLSICGGFK

C*

>Pt_003G177700

MGQQSLIYSFVARGTVILAEYTEFKGNFTGIA

AQCLQKLPASNNKFTYNCDGHTFNYLVEDGFT

YCVVAVESAGRQIPIAFLERVKEDFNKRYGGGKAAT

AVANSLNREFGSKLKEHMQYCVDHPEEISKLAKVKAQVSEVK

GVMMENIEKVLDRGEKIELLVDKTENLR

SQAQDFRQQGTKMRRKMWIQNMKMKLIVLGIIIALILIIVLSVCHGFN

C*

>Pp011188m.g

MGQQSLIYSFVARGSVILAEYTEFTGNFTSIA

SQCLQKLPSSNNKFTYNCDGHTFNYLVENGFT

YCVVASESAGRQIPIAYLERVKDDFNKRYAGGKAGT

AVANGLNREFGPKLKDHMKYCVDHPEEINKLAKVKAQVTEVK

GVMMDNIEKVLDRGEKIELLVDKTDNLR

SQAQDFRTQGTKMKRKMWFQNMKIKLIVVGIIILIGFVIFLSICHGFK

CT*

>Pp011218m.g

MGQQSLIYSFVARGTVILAEYTEFTGNFTSIA

SQCLQKLPATNNKFTYNCDGHTFNYLVDNGFT

YCVVAVEAIGRQIPIAFLERIKEDFTGRYGGGKAAT

AVANSLNKEFGSKLKEHMQYCVDHPEEISKLAKVKAQVSEVK

GVMMENIEKVLDRGEKIELLVDKTENLR

SQAQDFRQQGTQMRRKMWLQNMKIKLIVLGILIALILIIVLSVCNGFK

C*

>Tc_1EG002981

MGQQSLIYAFVARGTVVLADYTEFTGNFTSIA

SQCLQKLPASNNKFTYNCDGHTFNYLVDNGFS

YCVVAIESVGRQVPIAFLERIKEDFTKIYGGGKAAT

APANSLSREFGPKLKEHMQYCIDHPEEISKIAKVKAQVSEVK

GVMMENIEKVLDRGEKIELLVDKTENLR

SQAQDFRQQGTQMRRKMWLQNMKIKLIVLGILIALILIIILSVCGGFK

C*

>Tc_1EG020369

MGQQSLIYSFVARGTMILAEYTEFTGNFTSIA

AQCLQKLPASNNKFTYNCDGHTFNYLVENGFT

YCVVAVESAGRQVPIAFLERVKEDFNKRYGGGKAAT

ATANSLNREFGSKLKEHMQYCVDHPEEISKLAKVKAQVSEVK

GVMMENIEKVLDRGEKIELLVDKTENLR

SQAQDFRQQGTKMRRKMWLQNMKIKLIVLGILIALILIIVLSVCHGFK

C*

>SlVAMP721a_Solyc06g083530.2.1

MGQQSLIYSFVARGTVILAEYTEFTGNFTSIA

SQCLQKLPASNNKFTYNCDGHTFNYLVDDGFT

YCVVAVESVGRQVPIAFLERVKDDFTKKYGGGKAAT

AVANSLNKEFGPKIKEQMQYCVDHPEEINKLAKVKAQVSEVK

GVMMENIEKVLDRGEKIELLVDKTENLR

SQAQDFKTQGTKVRRKMWLQNMKIKLIVLGIIFALIMVIVLSICHGFN

CH*

>Sl_Solyc09g061620.2.1

MGQQSLIYSFVARGTVILAEYTEFTGNFTSIA

SQCLQKLPASNNKFTYNCDGHTFNYLVEEGFT

YCVVAVESVGRQIPIAFLERTKEEFTKKYGGGKAAT

AVANSLNREFGPKLKEQMQYCVDHPEEISKLAKVKAQVSEVK

GVMMENIEKVLDRGEKIELLVDKTENLR

SQAQDFRTQGTTMRRKMWLQNMKIKLIVLGIIIALILIIVLSVCGGFK

CH*

>Sl_Solyc09g091610.2.1

MGQQTLIYSFVARGTVILAEYTEFTGNFNSIA

SQCLQKLPASNNRFTYNCDGHTFNFLAESGFT

YCVVATESAGRQLPIAFLERIKDDFSKKYGGGKATT

ATPKSLSKEFGPKMKEQMKYCVDNPEEINKLAKVKAQVSEVK

GVMMQNIEKVLDRGEKIELLVDKTENLR

SQAQDFRQQGTKIRRKLWYENMKIKLIVLGIIIALILIIILSVCPGFK

CMS*

>Sl_Solyc12g098180.1.1

MGQQTLIYSFVARGTIILVEYSEFTGNFSSIA

TQCLQKLPPSSNKFSYNCDDHTFNFLSDNGFT

YCVVATESAGREIPLAFLDRVKNDFSKKYAGGKAAT

ASAKSLNREFGSKLKEHMKYCCDHPEEISKLSKVKAQVSEVK

GVMMENIEKVLDRGEKIELLVDKTDNLR

SQAQDFRQQGTKIRRKMWYENMKIKLVVFAIILVLILIIILSVCPGFK

CTS*

>TcVAMP72sym_Thecc1EG029360

MVQKSLIYAFVSRGEVILAEYTEFSGNFNSIA

FQCLQKLPSSNNKFTYNCDGHTFNYLVDNGYT

YCVVADESAGRQVPIAFLERIKDDFVSKYGSGKAAT

APANGLNKEFGPKLKEHMQYCVEHPEEISKLAKVKAQVSEVK

GVMMENIEKVLDRGEKIELLVDKTENLH

QQAQDFRSTGTKIRRKMWLQNMKIKLIVLGILIALILIIVLSVCHGFN

CGK*

>CsVAMP72sym_Cucsa.236490

MAQKSLIYAFVARGTVILAEYTEYSGNFNSIA

FQCLQKLPTANNKFTYNCDGHTFNYLVDNGYT

YCVVATESAGRQVPIAFLERIKDDFVSKYAGGKAST

APANSLNKEFGSKLKEHMQYCVDHPEEVSKLAKVKAQVSEVK

GVMMENIEKVLDRGEKIELLVDKTENLH

TQAQDFKTSGTKIRRKMWLQNMKIKLIVLGILIALILIIVLSVCRGFN

CGGK*

>PtVAMP72sym_a_Potri.012G119600

MNQKSLIYAFVSRGTVILAEFTEFSGNFNSIA

FQCLQKLPATNNKFTYNCDGHTFNYLADNGFT

YCVVADESAGRQVPMAFLERVKDDFVSKYGGGKAAT

AQANGLNKEFGPKLKEHMKYCADHPEEISKLAKVKAQVSEVK

GVMMENIEKVLDRGEKIELLVDKTENLH

SQAQDFRSQGTQIRRKMWLQNMKVKLIVLGILIALILIIVLSVCKGFN

CGK*

>CcVAMP72sym_Ciclev10016613

MSQKSLIYAFVARGNVVLAEYTEFSGNFNSIA

YQCLQKLPASNNKFTYNCDAHTFNYLVDNGYT

YCVVADESSGRQIPMAFLERVKDEFVSKYGGGKAAT

APANGLNKEFGPKLKELMQYCVDHPEEISKLAKVKAQVSEVK

GVMMENIEKVLDRGEKIELLVDKTENLH

QQAQDFRSTGTKMRRKMWLQNMKIKLIVLGILIALILIIVLSVCHGFN

CGK*

>FvVAMP72sym_gene20060

MGQKSLIYAFVARGTVILAEYTEFSGNFNSIA

FQCLQKLPATNNKFTYNCDAHTFNYLVDNGFT

YCVVADESAGRQVPIAFLERVKDDFVAKYGGGKAAT

AAANSLNKEFGSKLKEHMQYCVDHPEEISKLAKVKAQVTEVK

GVMMENIEKVLDRGEKIELLVDKTETLHHQKLKSYFEAVTYGNLHLINVPRLKHDLLKEI

KRSLCYGFGISFQQAQDFRNVGTKMRRKMWLQNMKVKLIVLGILIALILIIILSVCHGFN

CGK*

>PtVAMP72sym_b_Potri.015G118300

MSQKSLIYAFVSRGTVILADYTEFSGNFNSIA

FQCLQKLPATNNKFTYNCDGHTFNYLVDNGFT

YCVVAAESAGRQVPIAFLERVKDDFVTKYGGGKAAT

AQANGLNKEFGPKLKEHMQYCADHPEEISKLAKVKAQVSEVK

GVMMENIEKVLDRGEKIELLVDKTENLH

QQAQDFRSQGTQIRRKMWLQNMKVKLIVLGILIVLILIIVLSICKGFN

C*

>SlVAMP72sym_Solyc02g069150.2.1

MGQQKALIYAFVGRGNVILAEYTDFSGNFNSIA

YQCLQKLPASNNKFTYNCDGHTFNYLVDNGFT

YCVVAEESVGRQIPIAFLERIKDDFMSKYGSGKAAT

APPNSLNKEFGPKLKEHMQYCAEHPEEISKLAKVKAQVSEVK

GVMMENIEKVLDRGEKIELLVDKTENLH

HQAQDFRNTGTQIRRKMWLQNMKIKLIVLGILIALILIIVLSVCKGFN

CGK*

>MtVAMP721d_Medtr2g028790

M

ANNQNQKQLIYAFVSRGTVILAEFTEFSGNFNSIA

FQCLQKLPSTNNKFTYNCDNHTFNYLIDNGYT

YCVVADETTGRQVPMAFLERVKDDFVSKYGGEKAST

APPNSLNKEFGPKLKEHMQYCVDHPDEISKLAKVKAQVSEVK

GVMMENIEKVLDRGEKIELLVDKTDNLH

HQAQDFRSSGTSIRRKMWLQNMKVKLIVLGILIALILIIVLSVTRG*

>MtVAMP721e_Medtr4g083490

M

GQNQKSLIYAFVSRGSVILSEYTEFSGNFNSIA

FQCLQKLPASNNKFTYNCDGHTFNYLVDNGYT

YCVVADETVGRQVPVAFLERVKDDFVAKYGGGKAST

AAPNSLNKEFGPKLKEHMQYCVDHPEEVSKLAKVKAQVSEVK

GVMMENIEKVLDRGEKIELLVDKTENLH

HQAQDFRNSGTKIRRKMWLQNMKIKLIVLAILIALILIIVLPIVLKNK

*

>PpVAMP72sym_ppa011167

MGQKSLIYAFVARGTVILAEYTEFSGNFNSIA

FQCLQKLPATNNKFTYNCDGHTFNYLVDNGYT

YCVVADESSGRQVPIAFLERIKDDFVSKYGGGKAAT

APANSLNKEFGSKLKEHMQYCVDHPEEISKLAKVKAQVSEVK

GVMMENIEKVLDRGEKIELLVDKTENLH

QQAQDFRNVGTKMRRKMWLQNMKVKLIVLGILIALILVIILSVCHGFN

CGK*

>VvVAMP72sym_GSVIVG01012100001

MGQKSLIYAFVARGTVILAEYTEFSGNFNSIA

FQCLQKLPATSNKFTYNCDAHTFTYLLDNGYT

YCVVADESVGRQVPMAFLERIRDDFVARYGGEKAAT

APANSLNKDFSSKLKEHMQYCVDHPEEISKLAKVKDQVSEVK

GVMMENIEKVLDRGEKIELLVDKTHNLH

EQAQDFRSAGTKIRRKMWLQNMKIKLIVLGILVALILIIVLSVCHGFN

CGK*

>SlVAMP724_Solyc01g066940.2.1

MGQESFIYSFVARGTMVLAEYTEFTGNFPAIA

AQCLQKLPSSNNKFTYNCDHHTFNFLVQDGYA

YCVVSKESVGKQISIAFLERVRADFNKRYGGGKADT

AVAKSLNKEFGPVMKEHMQYIIDHADEIEKLLKVKAQVSEVK

SIMLENIDKAIERGEDLTILSGKTENLR

DSALEFKTKGTQIRRKMWYQNMKIKLVVFGIILLLVLIIWLSICRGFN

CTN*

>CsVAMP724_Cucsa.172230

MSQDSFIYSFVARGTMILAEFTEFTGNFPAIA

NQCLQKLPSANNKFTYNCDHHTFNFLVEDGYA

YCVVAKESLSKQISIAFLERMKADFKKRYGGGKADT

AVAKSLNKDFGPIMREHMKYIIEHAEEIEKLIKVKAQVSEVK

SIMLENLDKAFERGDNINTLADKTENLR

DQAQTYRGQGTKLRRKMWYQNMKIKLVVLVILLALVLIIWVSICHGFN

CTN*

>PpVAMP724_ppa011159

MSQESFIYSFVARGTMILAEYTEFTGNFPAIA

TQCLQKLPSSNNKFTYSCDHHTFNFLVEDGYA

YCVVAKDSVGKQISIAFLERMKADFRKRYGGGKADT

AIAKSLNKEFGPIMKEHMKYIIDHAEEIEKLLKVKAQVSEVK

SIMLENIDKAIDRGENLTVLVDKTETLR

SQAQDYRSKGTQMRRKMWYQNMKIKLVVFGILLLLVLVIWVSICHGFD

CTN*

>CcVAMP724_Ciclev10005844

MSQESFIYSFVARGTMILAEYTEFTGNFPAIA

AQCLQRLPSSNNKFTYNCDHHTFNFLVEDGYA

YCVVAKESVSKQISIAFLERMRADFKKRYGGGKADT

AIAKSLNKEFGPLMKEHMKYIIDHAEEIEKLIKVKAQVSEVK

SIMLENIDKAVDRGENIQNLADKTENLR

EQAQAYKKAGTQIRRKMWYQNMKIKLVVLGILLILVLIIWLSVCHGFD

CTN*

>PtVAMP724_Potri.008G209100

MSQESFIYSFVARGTMILAEYTEFTGNFPAIA

TQCLQKLPSSNDKFTYNCDHHTFNFLVEDGYA

YCVVAKETVSKQISIAFLERMKADFKKRYGGGKADT

AAAKSLNKEFGPIMKEHMKYIIDHAEEIEKLIKVKAQVSEVK

SIMLGNIDKAIDRGEAIATLADKTETLR

DQAQAYKKQGTQIRRKMWYQNMKIKLVVLGVLLILRSFGFIRLVSYLL

SKLAWREILSCH*

>VvVAMP724_GSVIVG01017751001

MSQESFIYSFVARGTMVLAEYTEFTGNFPAIA

TQCLQRLPSANNKFTYNCDHHTFNFLVEDGYA

YCVVAKESVGKQVSIAFLERMKADFKKRYGGGKADT

ATAKSLNKDFGPIMKEHMQYIIDHAEEIEKLLKVKAQVSEVK

SIMLENIDKTLERGENLTILADKTEDLR

SQAQQFKKQGSQVRRKMWFQNMKIKLVVLGILLILALVIWVSICHGFN

CSK*

>MeVAMP724a_cassava4.1_015754

MSQESFIYSFVSRGTMILAEYTEFTGNFPAIA

AQCLQRLPSSNDKFTYNCDHHTFNFLVEDGYA

YCVVAKESVSKQISIAFLERMKADFKKRYGGGKADT

AVAKSLNKEFGPIMKEHMKYIIDHAEEIEKLIKVKAQVSEVK

SIMLGNIDKAIDRGVTITTLADKTENLR

DQAQAYKKQGTQIRRKMWYQNMKIKLVVLGILLLLVLIIWLSICRGFD

CTN*

>MeVAMP724b_cassava4.1_015758

MSQESFIYSFVARGTMILAEYTEFTGNFPAIA

AQCLQRLPSSNDKFTYNCDHHTFNFLVEDGYA

YCVVAKESVSKQISIAFLERMKADFKKRYGGGKADT

AIAKSLNKEFGPIMKEHMKYIIDHAEEIEKLLKVKAQVSEVK

SIMLGNIDKVIDRGETITTLADKTENLR

DQAQAYKKQGTQIRRKMWYQNMKIKLVVLGILLLLVLIIWLSICHGFD

CTN*

>MtVAMP724_Medtr2g436870

MSQESFIYSFVARGTMVLAEYTEFTGNFPAIA

AQCLQKLPSSNNKFTYSCDHHTFNFLVEDGYA

YCVVAKESVSKQISIAFLERVKADFKKRYGGGKADT

AIAKSLNKEFGPVMKEHMKYIIDHAEEIEKLLKVKAQVSEVK

SIMLENIDKAIDRGENLSVLSDKTETLR

AQAQDFRKQGTQVRRKMWYQNMKIKLVVLGILLFLVLVIWLSICGGFN

CSN*

>AtVAMP724_AT4G15780.1

MGQESFIYSFVARGTMILAEYTEFTGNFPSIA

AQCLQKLPSSSNSKFTYNCDHHTFNFLVEDGYA

YCVVAKDSLSKQISIAFLERVKADFKKRYGGGKAST

AIAKSLNKEFGPVMKEHMNYIVDHAEEIEKLIKVKAQVSEVK

SIMLENIDKAIDRGENLTVLTDKTENLR

SQAREYKKQGTQVRRKLWYQNMKIKLVVLGILLLLVLIIWISVCHGFN

CTD*

>FvVAMP724_gene06875

MSQESFIYSFVARGIMILAEYTEFTGNFPAIA

AQCLQRLPSTNNKFTYNCDHHTFNFLVEDGYA

YCVVAKDSVGKQISIAFLERVKADFRKRYGGGKADT

AIAKSLNKEFGPIMKEHMKYIIDHAEEIEKLLKVKAQVSEVK

SIMLENIDKAIDRGENLTVLVDKTETLR

SQDAVIPRGQSSFISTFCA*

>TcVAMP724_Thecc1EG021196

MSQEPFIYSFVARGTMILAEYTEFTGNFPAIA

AQCLQRLPSSNNKFTYNCDHHTFNFLVEDGYA

YCVVAKDSVGKQISIAFLERMKADFKKRYGGGKADT

AIAKSLNKEFGPIMKEHMKYIIEHAEEIEKLLKVKAQVSEVK

SIMLENIDKAIDRGENLTTLADKTENLR

DQAQAYRKQGAQIRRKMWYQNMKIKLVVLGILLLLVLIIWLSVCHGFD

CTN*

>TcVAMP727_Thecc1EG044212

MNQKGLIYSFVAKGTVVLAEHTSYSGNFSTIA

VQCLQKLPSNSSKFTYSCDGHTFNFLIDNGFV

FLVVADESVGRSVPFVFLERVQDDFKQRYGASIKNEGLHPLA

DDDEDDDLFEDRFSIAYNLDREFGPRLKEHMQYCMNNPEEISKLSKLKAQITEVK

GIMMDNIEKVLDRGEKIELLVDKTENLQ

FQADSFQRQGRQLRRKMWLQNLQMKLMVGGAILVLIIILWLIACGGFK

C*

>SlVAMP727_Solyc11g006780.1.1

MNPKGLIYSFVARGTVVLAEHTPYSGNFSTIA

VQCLQKLPSNSSKYTYSCDGHTFNFLLDSGFV

FLVVADEPTGRSVPFVFLERVKDDFKKRYGSSIKNDGDPHPLA

DGDEEDDDLFGDRFSIAYNLDREFGPKLKEHMEYCMNHPDEMSKLSKLKAQITEVK

GIMMDNIEKVLDRGEKIELLVDKTENLQ

FQADSFQRQGRQLRRKMWFQNLQMKLMVGGAIVIFIIIVWLFACGGFS

C*

>MeVAMP727_cassava4.1_032126

MSQKGLIYSFVAKGTVVLAEHTPYSGNFSTIA

VQCLQKLPSNSSKYTYSCDGHTFNFLIDNEFV

FLAVADELSGRSMPFVFLERVKDDFKQRYGASIKNETHPLA

DDDDDDLFEDRFSIAYNLDREFGPRLKEHMQYCMSHPEEISKLSKLKAQITEVK

GIMMDNIEK

>PpVAMP727_ppa010737

MSHKGLIYSFVAKGGVVLAEHTSFSGNFSTIA

VQCLQKLPSSSSKYTYACDSHTFNFLLDNGFV

FLVVADESVGRSMPFVFLERVKEDFKQRYGSNNKIEGPHPLA

DDNEDDDLFEDRFSIAYNLDREFGPRLKEHMQYCMEHPEEISKLSKLKAQITEVK

GVMMDNIEKVLDRGEKIELLVDKTENLQ

FQADSFQRQGRQLRRKMWLQSLQMKLMIGGGILILIIILWLIACGGFK

C*

>CpVAMP727_evm.TU.supercontig_54.3

MSQKGLIYSFVAKGTIVLAEHTSYSGNFSTIA

VQCLQKLPSNSNKYTYSCDGHTFNFLIDSGFV

FLVVADESAGRSVPFVFLERVKDDFKQRYGASITDGGSHPLA

DDDDDDDLFEDRFSIAYNLDREFGPRLKEHMQYCMNHPEEISKIAKLKAQITEVK

GIMMDNIEKFIDEKPLERDINWLGAIV

FA*

>CsVAMP727_Cucsa.364630

MSQKGLIYSFVAKGSVVLAEHTSFSGNFSTIA

VQCLQRLPSNSSKCTYSCDGHTFNFLLDSGFV

FLAVADESVGRNMPFVFLDRVKDDFKQRYGSSIKDENPHPLA

DDEDDDDLFLDRFSVAYTLDREFGPKLKEHMQYCMSHPEEMSKLSKLKAQITEVK

GIMMDNIEKVLDRGERIELLVDKTENLQ

FQADNFHRQGRQLRRKMWLQSLQMKLMVGGGILVLFVILWFIVCGGFK

C*

>MtVAMP727_Medtr1g111890

MSQRGLIYSFVAKGTVVLAEHTQYTGNFSTIA

VQCLNKLPSNSTKYTYSCDGHTFNFLLDNGFV

FLVVADESIGRSVPFVFLERVKDDFNQRYGASIKIASDHPLA

DDDEDDDLFEDRFSIAYNLDREFGPSLKGHMQYCLTHPEEMSKLSKLKAQITEVK

GIMMDNIEKVLDRGEKIELLVDKTENLQ

FQADSFQRQGRQLRRKMWLQNLQMKLMVGGGILILVIILWVIACGGFK

C*

>VvVAMP727_GSVIVG01010710001

MSQKGLIYSFVAKGTVVLAEHTSFSGNFSTIA

VQCLQKLPSNSSKYTYSCDGHTFNFLIDSGFV

FLVVADESAGRGAPFVFLERVKDDFKQRYGGSIRSDGPHPLA

DEDDDDDDLFEDRFSIAYNLDREFGPKLKEHMQYCMNHPEEISKLSKLKAQITEVK

GIMMDNIEKVLDRGERIELLVDKTENLQ

FQADSFQRQGRQLRRKMWLQNLRLKLMVGGIVLVLIIILWLIACKGFK

C*

>AtVAMP727_AT3G54300.1

MSQKGLIYSFVAKGTVVLAEHTPYSGNFSTIA

VQCLQKLPTNSSKYTYSCDGHTFNFLVDNGFV

FLVVADESTGRSVPFVFLERVKEDFKKRYEASIKNDERHPLA

DEDEDDDLFGDRFSVAYNLDREFGPILKEHMQYCMSHPEEMSKLSKLKAQITEVK

GIMMDNIEKVLDRGEKIELLVDKTENLQ

FQADSFQRQGRQLRRKMWLQSLQMKLMVAGAVFSFILIVWVVACGGFK

CSS*

>FvVAMP727_gene00360

MSHKGLIYSFVAKGSVVLAEHTSFSGNFSTIA

VQCLQKLPSSTTKSTYSCDGHTFNFLLDNGFV

FLVVADESVGRSVPFVFLERVKADFMQRYAPSIKNEGPHPLA

DEDEDDALFEDRFSIAYNLDREFGPKLKEHMQYCMEHPEEMSKLSKLKAQITEVK

GVMMDNIEKVLDRGEKIELLVDKSENLQ

FQADSFQRQGRQLRRKMWLQSLQMKLLIGGGIFVLIIILWLIACGGFK

C*

>PtVAMP727a_Potri.010G239900

MSSQKGLIYSFVAKGNVVLAEHTSYSGNFSTIA

VQCLQKLPSNSSKYTYSSDGHTFNFLIDNGFV

FLVVADESVGRGVSFVFLERVKDDFNQRYGASIKNEAHPLA

DDDDDDDLFEDRFSIAYNLDREFGPRLKEHMQYCVNHPEEISKLSKLKAQITEVK

GIMMDNIDKVLDRGERIELLVDKTDNLS

FQADSFQRQGRELRRKMWLQNLKVKLVLGGTVLALIVIVWISVCGGFK

C*

>PtVAMP727b_Potri.008G019400

MSSQRGLIYSFVAKGNVVLAEHTSYSGNFSTVA

VQCLQKLPSNSSKYTYSCDGHTFNFLIDNGFV

FLAVADESAGRGLPFVFLERVKDDFKQRYSASIKNEAHPLA

DDDDDDDLFEDRFSVAYNLDREFGPRLKEHMQYCLNHPEEISKLSKLKAQITEVK

GIMMDNIEKVLDRGERIELLVDKTENLQ

FQADSFQRQGRQLRRKMWLQNLQMKLMVGGGVLVVILILWFVACGGFK

C*

>SiVAMP727_Si014368m.g

MNKQALIYSFVAKGSVVLAEHTAFSGNFSTVA

VQCLQKLPPNSTRSTYSCDGHTFNFLVDRGFV

FLVVADEATGRSVPFVFLERVREDFMQRYGSSIDEEGQHPLA

DDAEEDDFLFEDRFSIAYNLDREFGPRLKDHMQYCVNHPEEINKLSKVKAHLSEVK

GIMMDNIEKILDRGEKIELLVGKTETLQ

SQADSFHRHGRELRRKMWLQNLRFKLMVGGAIAALILILWLMVCRGFK

C*

>Si023178m.g

MAQAAEGGLIYGMVARGTVVVAEHTSYTGNFRDIA

AQCLHRLPAGNNRFTYTCDNHTFNFLVTDGYA

YCVVATESAGRQIPMAFLEMIKEDFNKRYAGGKAAT

ATANSLSRDFGPRLRDQMQYCTDHPEEVSRLSKVKAQVDQVK

GIMMENIDKVIDRGEQIDGLVTRTEQLH

DQAADFRQQGARVRRKMWYQNMKMKLIVLGIVVALILIIILSVCHGIC

K*

>SiVAMP724_Si030911m.g

MASPAPGGKGA

GEGGDKAEWLIYAFVARGTAVLAEYTEFTGNFPSIA

AQCLQRLPAGSSSASPGGSGAPARFSYSCDRHTFSFLLHRGYA

YCVVAKESVPKNVSVAFLERLKDDFMKRYGGGKADT

ALAKSLNKEYGPVIKQHIQYVLEHSEELDKTLKVQAQVSEVK

NIMLDNIEKTLGRGETLSELQDKTSDLR

SQAQEFKKQGVKIRRKTWLQNMKIKLVILGILLLLVVIVWVSVCQGFD

CTKHET*

>Si031049m.g

MGQQQLIYAFVARGTVVLAEYTEFTGNFTTIA

AQCLQKLPASNNKFTYNCDGHTFNYLVEDGFT

YCVVAVESVGRQVPIAFLDRVKEDFTKKYGGGKAAT

AAANSLNREFGSKLKEHMQYCVDHPEEVSKLAKVKAQVSEVK

GVMMENIEKVLDRGEKIELLVDKTENLR

SQAQDFRQQGTKVRRKMWLQNMKIKLIVLGIIIALILIIILSVCHGFN

CGKK*

>Si037146m.g

MGQQSLIYAFVARGTVILAEYTEFTGNFTTIA

SQCLMKLPASNNKFTYNCDGHTFNYLVEDGFT

YCVVAVESVGRQVPIAFLDRVKEDFTKRYGGGKAAT

AAANSLNREFGSKLKEHMQYCVDHPEEISKLAKVQAQVSEVK

GVMMENIEKVLDRGEKIELLVDKTENLR

SQAQDFRQQGTKVRRKMWLQNMKIKLIVLGIIIALILIIILSVCHGFK

CH*

>Si039081m.g

MGQQRTLVYSFVARGAAVLADHAEVSGNFASVA

AQCLQKLPANNNRFTYNCDGHTFNYHVHDGF

TYCVVATEAAGRQLPIGFIERVKEDFSKKYSGGKARS

ATANGLKREYGPKLKEHMRYCDQHPEEIDKLAKVKAQVTEVK

GVMMQNIEKVLDRGEKIELLVDKTEDLR

SQAQDFRQQGTKIRRKMWWENMKMKLIVFGIVVALILLIVLTVCKDFN

CW*

>Os_Os03g06960

MAPQKRTTLVYSFVARGAVVLADHAEVSGNFASVA

AQCLQKLPSTNNRHSYNCDGHTFNYHVHDGF

TYCVVATESAGRQLPVGFIERVKEDFSKKYSGGKAKN

ATANSLKREYGPKLKEHMKYCDAHPEEIDKLAKVKAQVTEVK

GVMMQNIEKVLDRGEKIELLVDKTEDLR

SQAQDFRKAGTKIRRKMWWENMKMKLIVFGIVVALILVIILTVCRDLN

CW*

>Os_Os03g58840

MGQQSLIYAFVARGTVILAEYTEFTGNFTTIA

SQCLMKLPASNNKFTYNCDGHTFNYLVEDGFT

YCVVAVESVGRQIPIAFLDRVKDDFTKRYAGGKAAT

AAANSLNRDFGSKLKEHMQYCVDHPEEISKLAKVKAQVSEVK

GVMMENIEKVLDRGEKIELLVDKTENLR

SQAQDFRQAGTQVRRKMWLQNMKIKLIVLGIIIALILIIILSVCHGFK

CK*

>Os_Os07g09600

MGQQSLIYAFVARGTVVLAEYTEFTGNFTTIA

AQCLQKLPASNNKFTYNCDGHTFNYLVEDGFT

YCVVAVESVGRQIPIAFLDRVKEDFTKRYGGGKAAT

AAANSLNREFGSKLKEHMQYCVDHPEEISKLAKVKAQVSEVK

GVMMENIEKVLDRGEKIELLVDKTENLR

SQAQDFRQQGTKVRRKMWLQNMKIKLIVLGIIIALILIIILSVCHGFK

CK*

>OsVAMP724_Os07g14540

MASPPGKKGEGGG

DGGGGKAEWLIYAFVARGTAVLAEYTEFTGNFPALA

AQCLQRLPASGGGGSGGGAPARFSYACDGHTFNFLLHRGYA

YCVVAKESVPKNVSVAFLERLKDDFMKRYGGGKADT

ALAKSLNKEYGPVIKQHMQYVLDHSEEIEKTLKVQAQVSEVK

NIMLENIEKTLGRGEKLSELQDKTSDLQ

SQAQEFKKKGVKIRRKTWLQNMKIKLVVLGILLLLVIIVWVSVCQGFD

CTKH*

>OsVAMP727_Os08g44430

MNGNKQSLIYSFVAKGSVVLAEHTAFSGNFSTIA

VQCLQKLPPNTSKSTYSCDGHTFNFLVDRGFV

FLVVADEAVGRSVPFVFLDRVKEDFMQRYGSSIDEEGQHPLA

DDADDDDFLLEDRFSIAYNLDREFGPRLKDHMLYCINHPEEISKLSKVKAHLTEVK

GIMMDNIEKILERGEKIELLVGKTETLQ

SQADSFHRHGRELRRKMWLQNLRFKLMVGGAVAALILFLWLIICGGFK

C*

>Os_Os12g44250

MAESKLIYAMVARGTVVLAEHTAYAGNFRDIA

AQCLQKLPAGDNRLTYTCDAHTFNFLIHQGYA

YCVVATESSGRQIPLALLDMIKEDFNKRYAGGKAAT

AAANSLSRDFGPRLGEQMKYCMDHPEEVSKLAKVKAQVSEVK

GIMMENIDKAIDRGQQIDVLVSRTEQLH

DQAADFRQQGTRVRRKMWYQNMKIKLIVLGIIIALILIIILSVCHGFK

C*

>AcVAMP724_Aquca_003_00772

MSQESFIYSFVARGTMILAEYTEFTGNFPAIA

TQCLQKLPSSNNKFTYNCDHHTFNFLVEDGYA

YCVVAKESAGKQVSIAFLERMKADFKKRYGGGKADT

AIAKSLNKEFGPVMKEHMQYIFDHADEIGKLLKVKAQVSEVK

SIMLENIDKALDRGEKLDILAEKTEDLR

SQAQEFKKQGTQVRRKMWYKNMKIKLVVLGVLLLLVLIIWVSVCQGFD

CTN*

>outgroup_AtVAMP711_AT4G32150.1

MAILYALVARGTVVLSEFTATSTNASTIA

KQILEKVPGDNDSNVSYSQDRYVFHVKRTDGLT

VLCMAEETAGRRIPFAFLEDIHQRFVRTYGRAVHTA

LAYAMNEEFSRVLSQQIDYYSNDPNADRINRIKGEMNQVR

GVMIENIDKVLDRGERLELLVDKTANMQG

NTFRFRKQARRFRSNVWWRNCKLTVLLILLLLVIIYIAVAFLCHGPT

LPSCI*

>AcVAMP721b_Aquca_025_00114

MGQQTLIYSFVARGTVILAEYTEFKGNFTSIA

SQCLQKLPASNNRFTYNCDSHTFNYLVDDGFT

YCVVAVESAGRQVPIAFLERIKDEFAKRYKGGKAAT

AVANSLNKEFGSKLKEQMQYCVDHPEEISKLAKVKAQVSEVK

GVMMENIEKVLDRGEKIELLVDKTENLR

SQAQDFRSQGTKLRRKMWWQNMKIKLVVLGIIIALVLIIILSVCHGFK

CT*

>AcVAMP721a_Aquca_034_00243

MGQQSLIYSFVARGTVILAEYTEFTGNFTSIA

AQCLQKLPASNNKFTYNCDGHTFNYLVEDGFT

YCVVAVESAGRQVPIAFLERVKDDFNKKYGGGKAAT

AVANSLNKEFGSKLKEQMQYCVDHPEEISKLAKVKAQVSEVK

GVMMENIEKVLDRGEKIELLVDKTENLR

SQAQDFRQQGTKMRRKMWLANMKIKLIVLGILIALILIIVLSVCGGFN

CGKN*

>AcVAMP727_Aquca_072_00106

MSQKGLIYSFVARGNVVLAEHTSFSGNFSTIA

VQCLQKLPSNSNKYTYSCDGHTFNFLIDNGFV

FLVVADEAVGRSIPYVFLDKVKEDFKQRYGASIGSEGPHALA

DEEDEDLFEDRFSIAYNLDREFGPRLKEHMEYCMSHPEEISKLSKLKAQITEVK

GIMIDNIEKVLDRGEKIELLVDKTENLQ

FQADSFQRQGRQLRRKMWLENLRMKLMVGGVVLVVIVILWLMVCKGFK

C*

>Bd_Bradi1g05030

MGQQSLIYAFVARGTVVLAEYTEFTGNFTTIA

SQCLAKLPSSNNKFTYNCDGHTFNYLVEDGFT

YCVVAVESVGQQMPIAFLVRIKDDFSKRYGGGKAAT

AAASSLNREFGSKLKEHMQYCVDHPEEINKLAKVQAQVSEVK

NVMMENIEKVLDRGEKIELLVDKTENLR

SQAQDFRQQGTQVRRKMWLQNMKIKLIVLGIIVALILIIILSVCHGFK

CK*

>Bd_Bradi1g05030(2)

MGQQSLIYAFVARGTVVLAEYTEFTGNFTTIA

SQCLAKLPSSNNKFTYNCDGHTFNYLVEDGFT

YCVVAVESVGQQMPIAFLVRIKDDFSKRYGGGKAAT

AAASSLNREFGSKLKEHMQYCVDHPEEINKLAKVQAQVSEVK

NVMMENIEKVLDRGEKIELLVDKTENLR

SQAQDFRQQGTQCK*

>Bd_Bradi1g53340

MASPAPKE

GGGKGEWLIYAFVARGIAVLAEYTEFTGNFPALA

AQCLQRLPAGSANPGSMPARLSYGCDGHTFNFLLDRGYA

YCVVAKESVPKNLTVAFLERMKDDFMKRYGGGKADT

ALAKSLNKDYGPVIKQHMQYVLDHSDEIEKTLKVQAQVSEVK

NIMLNNIEKTLDRGEKLTELQDKTSDLC

SQAQEFKKQGVKIRRKTWLQSMKLKLVILGILLLLVIIVWVSVCQGFD

CTKH*

>Bd_Bradi1g54680

MGQQSLIYAFVARGTVVLAEYTEFTGNFTTIA

AQCLQKLPASNNKFTYNCDGHTFNYLVEDGFT

YCVVAVESVGRQTPIAFLDRVKDDFTKRYGGGKAAT

AGASSLNREFGSKLKEHMQYCVDNPEEINKLAKVKAQVSEVK

GVMMENIEKVLDRGEKIELLVDKTENLR

SQAQDFRQQGTKVRRKMWLQNMKIKLIVLGIIIALILIIILSVCHGFK

CNK*

>Bd_Bradi1g73990

MAPAPPKKLVYSFVARGTVVLADHAEVSGNFASVG

TQCLQKLPPSNNRHSINCDGHTFNYHIHDGF

TYCVVATEAAGRQLPIGFIERVKEDFTKKYSGGKAKT

ASANGLKREYGPKLKEHLRYCDQHPEEIDKLAKVKAQVSEVK

GVMMQNIEKVLDRGEKIELLVDKTEDLR

SQAQDFKKQGTKIRQKMWWENMKIKLIVFGIVVALILLIVLTVCNDIR

CW*

>Bd_Bradi3g42850

MNGGSSKQTLIYSFVAKGSVVLAEHTAFSGNFSTIA

VQCLQKLPSNSTRSTYSCDGHTFNFLVDRGFV

FLVVAEEAVGRSVPFVFLERVKEDFMQRYGSSIDEEGQHPLA

DDADEDDFLFEDRFSIAYNLDREFGPRLKDHMQYCINHPEEISKLSKVKSHLSEVK

GIMMDNIEKILDRGEKIELLVGKTENLQ

SQADSFHRHGRELRRKMWLQNLRFKLMVGGGVAFLILILWLMVCKGFK

C*

>Bd_Bradi4g00397

MAAQGEEGGRLIYAMVARGTVVVAEQTAYTGNFRDIA

AQCLQKLPAGDNRFTYTCDGHTFTFLIHHGYA

YCVVASESAGREIPLAFLDNIKDEFVRRYAGGKADT

AAANSLTRDFGPKLKEQMQYCMDHPEELSRLSKVKVQVSEVK

GIMLDNIDKAIDRGEHIDVLVSRTEQLH

DQAADFRTQGTRVRRRMWYQNMKARLIVGGIVVAVVLIVVLTACHDRC

H*

>Pinustaeda_VAMP721

MGQQSLIYSFVARGTVVLAEYTEFKGNFTGIA

AQCLQKLPASNNKFTYNCDNHTFNYLVEDGFA

YCVVADESVGRQVPMAFLERVKEDFKRRYGGGRADT

AVANSLNRDFGSKLKEHMQYCIDHPEEISKLAKVKAQVSEVK

GVMMDNIEKVLDRGEKIELLVDKTENLR

FQAQDFQKKGTELRRKMWFQNMKVKLIVLGIVVALILIIVLSVCHGFN

C

>Pinustaeda_VAMP722

VVLAEYTEFKGNFTGIA

AQCLQKLPASNNKFTYNCDNHTFNYLVEDGFA

YCVVADESVGRQVPMAFLERVKEDFKRRYGGGRADT

AVANSLNRDFGSKLKEHMQYCIDHPEEISKLAKVKAQVSEVK

GVMMDNIEKVLDRGEKIELLVDKTENLR

FQAQDFQKKGTELRRKMWFQNMKVKLIVLGIVVALILIIVLSVCHG

>Pinustaeda_VAMP723

ARGTVVLAEYTQFTGNFTTIA

NQCLQKIPASNNKFTYNCDRHTFNYLVEDGYT

YCVVADESVGRQLPIAFLERIKDDFKKRYGGGKADT

AVAHSLNKDFGPKLKDHMQYCVDHPEEINKLAKVKAQVSEVK

GVMMENIEKVLDRGEKIELLVDKTENLR

FQAQDFQKQGTQIRRKMWFQNMKVKLVVLGIVFVLILIIWLSICHGFK

C

>Pinustaeda_VAMP727

MA

QQSLIYSFVARGNIVLAEHTSFSGNFSIIA

VQCLQKLPSNSNKFTYTCDNHTFNYLVDDGFV

FLVVADEAAGRQVPFLFLERVKEDFKRRYGGRAETSMA

HSLDKDYGYEEKFSVAYNLDREFGPILRDHMQYCMDHPEELSKFFKIKAQVSEVK

GIMMDNIEKVLDRGEKIELLVDKTEGLQ

FQADNFQRQGRQLRRKMWLQNLKFKLIVLGIVLVIMLIIWLSICKGFS

C

>Bv_4_081080_kgxf

MGQQNLIYSFVARGTVILAEYTEFTGNFTSIA

SQCLQKLPATNNKFTYNCDGHTFNFLVSDGFT

YCVVAVESVGRQIPMAFLERVKDDFNKRFGGGKAAT

AQAKSLNKDFGPKLKEHMQYCVDHPEEMNKLAKVQAQISEVK

GVMMENIEKVLDRGEKIELLVDKTENLR

SQAQDFRQQGTKMRRKMWFQNMKIKLIVLGIIIALILIIVLSICHGFN

CGK

>Bv_7_169720_cteq

MGQQSLIYSFVARGTVILAEFTEFSGNFTSVA

TQCLQKLPSSSSKFTYTCDGHTFNYLVDNGFT

YCVVAVESAGRQIPIAFLERVKDEFTKKYGGGKAAT

AAAKGLNKEFGPKLKEQMQYCVDHPEEINKLAKVKAQVSEVK

GVMMENIEKVLDRGEKIELLVDKTENLR

SQAQDFRTQGTKIRRKMWWENMKIKLVVIAIIVALIIILILSICGGFK

CTH

>Sh_StHe62GB1_11967

MAQGSRKTLIYALVARGTPPVVLAEYTEFSGNFNSIA

YQCLQKLPSSNNKFTYNCDNHTFNYLVYDGFT

FCVVAEESAGRQLPMAFLERVRDEFVAKYGGGKAAT

APANGLNKEFGPKMKEHMQYCIEHPEEISKLAKVKAQVSEVK

GVMMENIEKVLDRGEKIELLVDKTENLH

HQAQDFRTTGTKIRRKMWLQNLKIKLIVLAIIIALILIIVLSICRGFN

CGN

>Sh_StHe62GB1_50189

?KLP

ATNNKFTYNCDGHTFNYLVEDGFT

YCVVAVESVGRQLPIAFLERIKDDFTKKYGGGKAAT

SIANSLNREFGPKLKEQMQYCVDHPEEINKLAKVKAQVSEVK

GVMMENIEKVLDRGEKIELLVDKTENLR

SQAQDFRTQGT?DEEKNVAAEHEDKVDRPRHHHCLDLNHRFISMW

>Sh_tHe62GB1_18530

REPNQPWAGGKRRDQQNKEKGSSDKITHSGDRFSGA

QSAMGGQESFIYSFVARGTMVLAEYTEFTGNFPAIA

AQCLQRLPSSNNKFTYKCDNHIFNFLVEDGYA

YCVVAKESVGKQISIAFLERIKADFKKRYGGGKADT

AVAKSLNKEFGPLMKEHMQYIIDHADEIVKLLKVKAQVSEVK

SIMLENIDKTIERGENLTVLNDKAEDLR

YSAQEFKKKGTEIRRKMWYQNMKVKLVVLGIILLLVLIIWLSVCRGFD

>Sh_StHe62GB1_48984

AAFFLPSHFSFSPLFPPRLKPPAVPPSVFLRLSSDCLGERRAYCWAAS

FLKMTPKGLIYSFVAKGTVVLAEHTPYSGNFSTIA

VQCLQKLPSSSSKYTYSCDGHTFSFLLDGGFV

FLVVADESMGRSVPFVFLERVKDDFKQHYGDSIKSDGQHPLA

DDEDEDDDLFEDRFSIAYNLDREFGPKLKEHMQYCMNHPEEMNKLSKLKAQITEVK

GIMMDNIEKVLDRGEKIELLVDKTENLQ

FQA?QFSETRKAASSADVAANSPHKADGWRRHFNLNLPHLVDVW

>Sp_13G0003200

MSRQPLVYSFVAKGTIVLAEHTSFSGNFSTLA

IQCLQRLPSNSNKFTYSCDGHTFNFLVDSGFV

FLVVADESTGRSIPFVFLDRVKDDFMQRYGASINAGESHPLA

DEDDDDLFEDRFSIAYNLDREFGPRLKEHMNYCITHPEEMSKLSKLKAQITEVK

GIMMDNIEKVLDRGEKIELLVDKTESLQ

FQADSFQRQGRQLRRKMWLQNLRMKLMVVGAVLALAVIVWLMVCRGFK

C*

>Sp_Spipo18G0034800

MGQQSLIYGFVARGTVVLVSFTEFTGNFSSIA

AQCLQKLPASNNKFTYTCDGHTFNYLVEEGYSS

YCVVAAESAGRQVPIAFLERVKEDFIKRYGGGKAAT

VDADGLTREFGPKLKDHMQYCVDHPEEVSKLAKVKAQVSEVK

GVMMENIEKVLERGEKIELLVDKTENLR

SQAQDFRQQGTNVRRKMWLQNMKIKLIVLGILITLILIIVLAACHGFN

C*

>Sp_Spipo25G0001400

MGQKSLIYSFVARGAVIVASYAESSGNFTNIA

SQCLQKLPARNNKFTYNCGNQSFNYLVEDGYT

YCVVASESVGTQLPMAFLDRVKEDFIKRYGGGKATT

APANSLDRDFGPKLKNQMQYCVDHPEELSKLARVKDQVLEVK

GVMMNNIENVLSRGEKLDVLVDKAEDLR

SHAQVFKNQGTEIKKKMWMQNMKMKLVILAIIIALILIIVLSICGGSR

C*

>Sp_Spipo3G0038000

GTAASFIYSFVARGTMVLAEYTEFTGNFPAIA

VQCLQKLPSSNSRFTYACDHHSFNFLVEDGYA

YCVVVKESVGKQICMAFLDRLKVDFKKRYGGGRADT

ALAKSLNKEFGPVIKEHMQYVIEHADELIKLLKVKAQVSEVQ

SIMLENVEKAVDRGQKLGDLAEKATDLR

SQAQDFKKKGGQVRRKMWLQTMKVKLVVLAVLLLLVLIVWVTVCQGFD

CTK*

>Sp_Spipo8G0066900

MGQQSMIYSFVARGTVILASYTEFSGNFTSIA

SQCLQKLPASNNKFTYNCDGHTFNYLVEDGYT

YCVVAVESVGRQVPIAFLERIKEDFVKRYGGGKAAT

AAANSLDREFGSKLKEHMQYCVDHPEEISKLAKVKAQVSEVK

GVMMENIEKVLDRGEKIELLVDKTENLR

SQAQDFRQQGTKLRKKMWLQNMKIKLIVLGIIIALILIIVLSVCRGFK

C*

>Sp_Spipo9G0038000

MSKQFLIYSFVAKGTVVLAEHTSFSGNFSTIA

IQCLQKLPLGNNKFTYTCNGHTFNFLVDNGFV

FLVVADEAALRGLPFVFLERVKDDFMRRYETIIKAGGSHALD

VEEDDDLFEDKFSIAYNLDRDFGPRLKEHMSYCTRHPEEMIKVSKLKSQITEVK

GIMIDNIEKVLDRGDKIELLVEKTGTLQ

FQAEGFQRQGRQLRRKIWLHSLRLKMAIVGALLLLAVTLWLAACKGFR

CS*

>Dc_Dca21887.1

MGQQNLIYSFVARGTVILAEYTEFTGNFTSIA

SQCLQKLPATNNKFTYNCDGHTFNFLVSDGFT

YCVVAVESVGRQIPMAYLERVKDDFNKRYGGGKAAT

AVAKSLNKEFGPKLKEHMQYCVDHPEEMNKLAKVQAQISEVK

GVMMENIEKVLDRGEKIELLVDKTENLR

SQAQDFRQQGTQMRRKMWLQNMKIKLIVLGIIIALILIIVLSVCHGFN

CGK

>Dc_Dca51992.1

MGQQNLIYSFVSRGDSILAEYTEFSGNFTTIA

SQCLQKLPSTNNKFTYNCDGHTFNFLVSDGFT

YCVVAVESVGRQIPMAFLERVKDDFNKRYGGGKAAT

AAAKSLNKEFGPKLKEHMQYCVDHPDEMNKLAKVQTQISEVK

GVMMENIEKVLDRGEKIELLVDKTENLR

SQAQDFRQQGTKMRRKMWLQNMKIKLIVLGIIIALILIIVLSVCGGFH

CGK

>Dc_Dca39015.1

MVMVRVEEVKANWLRYFSMPGYRVQLQKGNVCLHRLFENAGHGKVRWTQDEEKEGP

YLWIATDSDGPWFRDKEATLEFTTVPADWKSKAIARANKKNHTVFRGNFSTIA

VQCLQKLPSSGSKYTYTCDGHTFNFLKDNGFV

FLVVADEAAGRSLPYVFLERVKDDFKQRYAGSIKSGDAHPLA

DENDSDDDLFEDRFSIAYNLDREFGPRLKEHMHYCMNHPEEMSRLSKVKAQIAEVK

GIMMENIEKVLDRGEKIELLVDKTENLQ

FQADSFQRQGRQLRRKMWFQNLQMKLIAGGSLLAFIIIVWLIACRGFK

C

>Nn_NNU_011988RA

MGQQSLIYSFVARGTVVLAEYTEFTGNFTSIA

AQCLQKLPATNNKFTYNCDSHTFNYLVENGFT

YCVVAVESVGRQIPVAFLERVKDDFNKRYGGGKAAT

AVANSLNKEFGPKLKEHMQYCVDHPEEVGKLAKVKAQVSEVK

EVLDRGEKIELLVDKTENLK

SQAQDFRQQGTKMRRKMWLQNMKIKLIVLGIIIALILIIVLSICHGFN

C

>Nn_NNU_022328RA

MGQQSLIYSFVARGTVILAEYTEFKGNFTSVA

SQCLQKLPASNNKFTYNCDNHTFNYLVENGFT

YCVVAVESAGRQIPIAFLERVKDDFSKRYGGGKGTT

ASAKSLNKEFGPKLKQHMQYCVDHPEEISKLAKVKAQV

LDRGEKIELLVDKTENLR

SQAQDFRQQGTKLKRKMWWENMKIKLIVLGIFVALVLIIVLSICHGFN

C

>Nn_NNU_015372RA

MSQQESFIYSFVARGTMILAEYTEFTGNFPAIA

SQCLQKLPSSNNKFTYNCDHHTFNFLVEDGYA

YCVVAKES?GKQVSIAFLERIKADFKKRYGGGKADT

AVAKSLNKEFGPVMKEHMQYIIDHAEEIGKLLKVKAQVSEVK

SIMLENIDK

>Nn_NNU_002506RA

MSQKGLIYSFVAKGNVVLAEHTSFTGNFSTIA

IQCLQKLPSNSSKYAYSCDGYIFNFLIDSGFV

FLVVADEAAGRSLPFVFLDRVNDDFKQRYGASISNAGPHPLA

DDDDDDLFEEDRFSVAYSLDREFGPRLKEHMQYCMNHPEEISKLSKLKAQITEVK

GIMMDNIERVLDRGEKIELLVDKTENLQ

FQADSFQRHGRQLRRRMWLQNIRLKLMVGGTILAVIILLWLLSCRGFK

C

>Nn_NNU_022742RA

MKTPLP

LVEGADPESNELQDIGGEGQKSVVRMDVA

YCVVADETVGRQVPMAFLERIKDDFVSRYGGGKAAT

APANSLNKEFGSKLKEHMQYCIDHPEEISKLAKVKAQVSEVK

GVMMENIEKVRVD

>Nn_NNU_009259RA

MSQQESFIYSFVARGTMILAEYTEFTGNFPAIA

SQCLQKLPSGNNKFTYSCDHHTFNFLVEDGYA

YCVVAKESVGKQVSIAFLERMKADFKKRYGGGKADT

AIAKSLNKEFGPVMKEHMQYIIDHAEEIEKLLKVKAQVSEVK

SIMLENIDK

>Ug_Scf00001.g272.t1

MGQQELIYSFVARGTVILADYTEFKGNFTSVA

AQCLQKLPASSNRFTYSCDGHTFNYHLDNGFSIILETLRILFAAIGFSF

QGNCYVSLNDASCFIAAFCVVAVESAGRQLPIAFLERVKEDFNKRYAGGKAGN

AGANGLKKEFGPKLKAHMQYCIDHPEEINKLAKVKAQVSEVK

GVMMQNIEKVLDRGEKIELLVDKTDNLR

SQAQDFKQQGTKMKRKMWVRNMKVKLIVFGIVVALALVVFLSICHNFK

CT

>Ug_Scf00052.g5585.t1

MVQKSLIYSFVARGTVILAEYTEFSGNFTGIA

AQCLQKLPATSSKFTYNCDGHTFNYLVDDGFT

YCVVAVESIGRQLPIAFLERIREDFAKKFAGGKAAA

ATANSLNGEFGPKLREHMQYCVDHPEEISKIAKVKAQVSEVK

GVMMENIEKVLDRGEKIEILVDRTENLR

SQAQDFRTQGTQMRRKMWLQNMKIKLIVLAIIVALILIIILSACGGFR

CGSRT

>Ug_Scf00081.g7411.t1

MGQQSLIYSFVARGTVILSEYTEFTGNFTGIA

AQCLQKLPATNNKFTYNCDNHTFNYLVEDGFT

YCVVAVESVGRQIPIAFLERIKEEFVKKYGGGKAAT

SVANSLNKEFGPKLKEQMQYCVDHPEEISKLAKVKAQVSEVK

GVMMENIEKVLDRGEKIELLVDKTENLR

SQAQDFKVQGTKMRKKMWLQNMKIKLIVLAIIIALILIIVLSVCHGFK

CH

>Ug_Scf00101.g8538.t1

MNRKSESAAQIGGKEATTPRASVYLAAGGAKLTTISHSPRLKNLLEIQLRFQKVMNSW

FSEMSPKGLIYSFVAKGTIVLAEHTPYSGNFSTIA

VQCLQKLPSNSSKYTYSCDGHTFNFLLDNGFV

FLVVADESMGRSVPFVFLERVKDVFNNCYGDSIHRGSQHPLA

DGDDEDDDLFEDRFSIAYNLDREFGPKLKEQMDYCMGHPEEMSKLSKLRAQITEVK

GIVLDNIEKVLERGEKIELLVDKTENLQ

FQADNFQRHGRQLRRKMWLQNFHMKLMIGGAILILICLFWFMFR

>Ug_Scf00121.g9506.t1

MAQKSLIYSFVARGTVILAEYTEFSGNFTGIA

AQCLQKLPATSSKFTYNCDGHTFNYLVDDGFT

YCVVAVESIGRQLPIAFLERIKEEFTTKYAGGKAAT

ATANSLNREFGPKLREQMQYCVDHPEEISKIAKVKAQVSEVK

GVMMENIEKVLDRGEKIEILVDRTENLR

SQAQDFRTQGTQMRRKMWFQNMKVKLIVLAIIVALILIIILSACGGFR

CGSRA

>Ug_Scf01362.g24617.t1

MGQQSLIYSFVARGTVILAEYTEFTGNFTGIA

AQCLQKLPATNNKFTYNCDNHTFNYLVEDGFT

YCVVAVESVGRQIPIAFLERTKEDFIKKYGGGKAAT

AVANSLNKEFGPKLKEQMQYCVDHPEEISKLAKVKAQVSEVK

GVMMENIEKVLDRGEKIELLVDKTENLR

SQAQDFKMQGTKMRKKMWLQNMKVKLIVLAIIVALILIIVLSVCHGFK

CH

>Ginkgo_EX931549.1

NKFTYNCDRHTFNYLVEDGFT

YCVVAVESVGRQLPMAFLERIMDDFKKRYGGGRADT

AVAHSLNKEFGSKLKEHMQYCIDHPEEISKIAKVKAQVSEVK

GVMMENIEKVLERGEKIELLVDKTENLR

FQAQDFQKQGTQLRRKMWFQNMKVKLIVLGIVLILILIIWLSICHGFK

CGK

>Ginkgo_EX938043.1

WIQSFG

PKLRDHMQYCMDHPEEMSKLSKIKAQVSEVK

GIMMDNIEKVLDRGEKIELLVDKTEGLQ

FQADNFQRQGRQLRRKMWLQNLKFKLIVLGIALLIILIIWLAVCKGFS

CT

>Ginkgo_Gb9983_c0_seq1

MGQQSLIYSFVARGTVVLAEYTEFTGNFTSIA

AQCLQKLPASNNKFTYTCDNHTFNYLVEDGFA

FCVVADESVGRQLPIAFLERIKEDFKKRYGGGRADT

AVAHSLNKDFGSKLKEHMQYCVDHPEEISKLAKVKAQVSEVK

GVMMENIEKVLDRGEKIELLVDKTENLR

FQAQDFQKQGTQLRRKMWFQNMKVKLIVLGILVALILIIVLSVCHGFN

CSNK*

>Ginkgo_Gb16042_c0_seq1

MR

QQSLIYSFVARGNVVLAEHTSFSGNFSTIA

IQCLQKLPSNSNKFTYTCDGHTFNYFVEDGFV

FLVVADEALGRQVPFVFLERVKEDFKQRYGGGKADTGVA

HSLDKDYGPKLRDHMQYCMDHPEEMSKLSKIKAQVSEVK

GIMMDNIEKVLDRGEKIELLVDKTEGLQ

FQADNFQRQGRQLRRKMWLQNLKFKLIVLGIALLIILIIWLAVCKGFS

CT*

>Ginkgo_Gb21921_c0_seq1

MAFLERIKDDFKKRYGGGRADI

AVANSLNKEFGPKLKEHMEYCVSHPEEINNFSKVKAQVSEVK

GVMMDNIEKVLERGETIEVMVDKTNNLR

SQAQDFRNQGNQIRRKMWFQNMKIKLIVLGIILSLILIIWLSVCRGFK

CH*

>Selagionella_164037

MGQDSLIYSFVARGTVVLAEYTGFSGNFSTIA

IQCLQKLPANNNKFTYTCDKHTFNYLVEDGFT

YMVVADEDFGRQIPFAFLERVKEDFKRRYGGGKADTAIA

NSLDKEYGPKLKEHMQYCVTHPDEMNKIAKIKAQVSEVK

GIMMDNIEKVLDRGEKIELLVDKTDNLR

FQADNFRRQGRDLRRKMWLQNMKVKLIVLGIVIVLILIIWLSICKGFK

CK*

>Selagionella_235084

MAGLIYSFVSRGNVVLAEFTAFSGNFSTIA

VQCLQKLPANNNRFTYTCDRHTFNYLIEEGFT

YMVVADEDYGRQVPFAFLERVKEDFRRRYVGGRAETAVA

HSLDREFGPKLKEHMEYCADHPEEMNKIARIKQQVSEVK

GIMMDNIEKVLERGEKIELLVDKTENLR

FQADNFQRQGRQLRRKMWLQNMKMKLIVLAIVLFVIVVIWLAICHGFS

CK*

>Mp_Mapoly0008s0223

MGAKNGLIYCFVARGTGPGVVVLADYSPFEGNFNKIA

LECAQKLAANNHSITYTCDRHTFNFLVEDGIT

YLVVAEESFPRKIPFAFLARVKDDFRKKFGGEAGADMA

VAHSLDKRFRPIMKEHMTFCLQHPEELDKVSKIQKQVDDVK

GVMMLNIDKIIERHERLEVIEDKAGNLA

NEAQQFQKKTNTLKNNLWWQNMKVKLFVLLLIVVVILVIWLSICHGFS

CSSGGGGGGNNPPANTGSGTAPPGGRRLL*

>Mp_Mapoly0008s0229

MGSILSSRSSHSSPRAKNGLIYSFVARGPGPQVVVLAEYTPVKGNFKKIA

LECAQTLDANNHSVTYTRDSHTFNFLVEDGFA

YLVVAEESLRWIPFAFLALVKDDFREKFPGAYKD

KANSLNKRFRPIMKKHMTFCVNRPEELDKVANIQNQLDEMK

GIAWRHINKFMDHQERLEDVFKKSGSLV

NEAQHFQRRTNRLKIYLCCQSMKVKLIVLLLIVLLAVISQKLYRFFM

KKLT*

>Mp_Mapoly0076s0053

MGVNSLIYSFVARGTVVLAEYTAFSGNFSTIA

VQCLQKLPANNNKFTYTCDRHTFNYLVEDGFT

YLVVADEDFGRQIPFAFLERVKEDFKRRYGGGRADTAIA

HSLDKEFGYEGKLDVAYSLDKEFGSKLKEHMTFCVEHPEEMNKLSKIKAQVSEVK

GIMMDNIEKVLDRGEKIEVLVDKTDNLR

TQADNFQRQGRQLRRKMWLQNFKVKLIVLAIIIVVILIIWLSICKGFK

CN*

>Phys_Pp3c12_11090

MGTQSLIYSFVARGPTVLAEYTAFSGNFSTIA

VQCLQKLPPNNNKFTYTCDRHTFNYLVEEGYT

YLVVADEEFGRQIPFAFLERVKEDFKRRYAGGKADSAIA

HSLDKEFGPKLKDHMQYCVDHPDEMNKISKIKSQVAEVK

GIMMDNIEKVLDRGEKIELLVDKTENLR

FQADNFQRQGRQLRRKMWFQNMKVKLIVLAIIIVVIIIIWLSICRGFT

CK*

>Phys_Pp3c14_26140

MGDANLIYSLVSRGTTVLAEYTSFAGNFSQIA

MQCLVKLPAANNKHTYVMDRHTFNFLVQDGFT

YLVVAEEDFGRQIPFAFLDRVKDDFKHRYQGGKADL

AVSHSLDAEFGPRLKEHMDFCERNPEEIRKMSKIKSQVAEVK

GIMMENIDKVLVRNEKIDLLVDRTSHLQ

SDAHNFQRQGKKIRYKLWCQNYRLKLLVLVLIIIVAFIIYLSICRGFV

CYNPGVPGTPPAPGTPPGQGL*

>Phys_Pp3c17_22240

MGDARLIYSFVARGTTVLAEHAIYAGNFSQIA

VQCLLKLPAGTSKQTYVMDRHTFNFFVENGFT

FLVVAEEALGRLIPFAFLERVKDDFKHHYQGGRADL

AVSHSLDAEFGPKLKEHMDFCMENPEEIKKISRIKSQVAEVK

GIMMENIDKVLDRSDKIDLLVDRTTHLQ

SSAAEYQRAGVRIRRRLWWQHFRLKLLVLLLIVVVAFIIYLSICRGFI

CHNPAVPGTPPAPGTPPGPL*

>Phys_Pp3c2_2310

MEEGLIYSFVSRGTTVLAEYASVSGNSNRIA

AQCLAKLPGGNNKHTYVCDRHTFNFLVEDGFT

FLAVADEDFSRQIAFAFLDRVKNDFQHRYQGGRADL

AVTYSLNAEFGPRLKEHMDFVAANPEEIKKMSKIKSQVAEVK

EIMMVNIEKLLDRNERIDLLVGKTDDLH

SNAHVFEKQGNQIRRRAWCAHFKLKLLVLVLIIIVAFIIYLSICRDFI

CHNPGMPGTTPANVPPAE*

>Phys_Pp3c4_13580

MGTQSLIYSFVARGSTVLAEYTAFSGNFSTIA

VQCLQKLPPNNNKFTYTCDRHTFNYLVEEGYT

YLVVADEEFGRQIPFAFLERVKEDFKRRYAGGKADSAIA

NSLDKEFGPKLKDHMQYCVDHPDEMNKISKIKSQVAEVK

GIMMDNIEKVLDRGEKIELLVDKTENLR

FQADNFQRQGKQLRRKMWFQNMKVKLIVLAIIIVIIIIIWLSICRGFT

CSNR*

>Ma08_p20110.1

MGQQSLIYSFVARGTVILAEYTEFSGNFNSIA

AQCLQKLPASNNKFTYNCDGHTFNYLVEDGYT

YCVVAVESVGRQIPIAFLERVKEDFNKKYGAGKAAT

ASANSLSREFGSKLKEHMQYCVDHPEEISKLAKVKAQVSEVK

GVMMENIEKVLDRGEKIELLVDKTENLH

SQAQDFRQQGTKMRRKMWLQNMKIKLIVLGIIIALILIIVLSVCHGFK

C

>Ma07_p01750.1

MGQQSLIYSFVARGTVILAEYTEFQGNFTSIA

AQCLQKLPSSNNRFTYNCDGHTFNYLVEDGYT

YCVVAVEALGRQIPIAFLDRIKEDFNKRYGGGKAAT

AVANSLNREFGSKLKEHMQYCVDHPEEISRLAKVKAQVSEVK

GVMMENIEKVLDRGEKIELLVDKTESLH

SQAQDFRQQGTQMRRKMWFQNMKIKLIVLAIIIALILIIILSVCHGFK

C

>Ma09_p10640.1

MGQQSLIYSFVARGTVILAEYTEFSGNFNSIA

AQCLQKLPASNNKFTYNCDGHTFNYLVEDGYT

YCVVAVESVGRQIPIGFLDRVKEDFNKRYGGGKAAT

ASANSLSREFGSKLKEQMQYCVDHSEEISKLAKVKAQVSEVK

GVMMENIEKVLDRGEKIELLVDKTENLR

SQAQDFRQQGTKMRRKMWLQNMKVKLIVLGIIIALILIIVLSICHGFK

C

>Ma04_p28820.1

MGQQSLIYSFVARGTVILAEYTEFQGNFTGIA

AQCLQKLPSSNNRFTYNGDGHTFNYLVEDGYT

YCVVAVESVGRQIPIAFLDRIKEDFNKRYGGGKAAT

ARANSLNREFGSKLKEHMQYCVDHPEEISKLAKVKAQVSEVK

GVMMENIEKVLERGEKIELLVDKTENLR

SQAQDFRQQGTKMRRKMWMKNMKIKLIVLGIIIALILIIILSVCHGFK

C

>Ma09_p25870.1

MGQQSLIYSFVARGTVVLAEFTEFSGNFNSIA

AQCLQKLPASNNKFTYNCDGHTFNYLVEDGYT

YCVVAVESVGRQIPIAFLERVKEDFNKRYGGGKAAT

AAANSLTREFGSKLKEHMQYCVDHPEEISKLAKVKAQVSEVK

GVMMENIEKVLDRGEKIELLVDKTENLR

SQAQDFRQHGTQMRRKMWLQNMKVKLIVLGIIIALVLIIVLSVCHGFK

C

>Ma02_p19450.1

EQRSLIYSFVARGTAILAEYTEFTGNFTTIA

SQCLQKLPATNNKFTYNCDGHTFNYLVDDGFT

YGVVAAESFGRQVPIAFLERVKEDFSKRYGGGKAAT

AAANSLTREFGSKLKEHMQYCVDHPEEISKLAKVQAQVSEVK

GVMMENIEKVLDRGEKIELLVDKTENLH

SQAQDFRQQGTKMRRKMWLQNMKIKLIVLGIIIALILIIILSVCHGFK

C

>Ma06_p06060.1

MGQQSLIYSFVARGTVILAEYTEFTGNFTTVA

SQCLQKLPATSNKFTYNCDGHTFNYLVQDGYT

YCVVAVESAGRQIPIAFLERVKEDFNKRYGGGKAST

ATAKSLNREFKSKLKEHMKYCVEHPEEINKLAKVKAQVSEVK

GVMMENIEKVLDRGEKIELLVDKTENLR

SQAQDFRQQGTKMKRKLWIENMKIKLIVFGIIIALILLIFLSICHGFQ

C

>Ma08_p33240.2

KQPLIYSFVAKGAVVLAEHTTFSGNFSTIA

VQCLQKLPRNSNKFTYSCDGHTFNFLLDKEFV

FLVVADEAVGRSVPFVFLERVKEDFMQRYGAIIDGGGPHPLA

DEDEDDDLFEDRFSIAYNLDREFGPRLKEHMQYCINHPEEMSKLSKLRAQITEVK

GIMMDNIEKVLDRGEKIELLVDKTESLQ

FQADSFQRQGRQLRRKMWLQNIRFKLILAGTLIALILIIWLRACGGFK

C

>Ma08_p33240.1

KQPLIYSFVAKGAVVLAEHTTFSGNFSTIA

VQCLQKLPRNSNKFTYSCDGHTFNFLLDKEFV

FLVVADEAVGRSVPFVFLERVKEDFMQRYGAIIDGGGPHPLA

DEDEDDDLFEDRFSIAYNLDREFGPRLKEHMQYCINHPEEMSKLSKLRAQITEVK

GIMMDNIEKVLDRGEKIELLVDKTESLQ

FQADSFQRQGRQLRRKMWLQNIRFKLILAGTLIALILIIWLRACGGFK

C

>Ma09_p31500.1

GKEGWFIYGFVARGTVVLAEYTEYTGNFPAIA

AQCLQKLPSSNKLSTYACDAHTFIFLVHNGYA

YCVVTKDSVVKNVSIAFLERLKADFTKRYGGGKADT

ATAKSLNKEFGPVIKGHMQYIIDHAEEVEKLLKVKVQVSEVK

NIMLENIDKTLERGEKLTDLEAKASDLR

NEAQGFKKQGTRIRKKMWLQNMKIKLVVLGILLFLVVIIWVSVCRGFD

CTK

>Ma08_p03170.1

QKLLIYSFVARRTMILAEYAEFKGNFTRIA

AQCLENLSGRNNKFSFNCDGRTFNYLVEDGY

TFCVVAVESVDREIPIFFLERVKEEFNKRYGGNAAT

AAAKSLSREFGSKLKEHMQYCADHPEEISKMAKLQAQVSELK

SAMIEKIEKVLDNKENVDVLVEKAKNLY

SQAQDFRLQGTTVRRNPWLQDM

>Ma08_p03170.2

MGQKLLIYSFVARRTMILAEYAEFKGNFTRIA

AQCLENLSGRNNKFSFNCDGRTFNYLVEDGYSECSRVSVSEIA

NLAAFCVVAVESVDREIPIFFLERVKEEFNKRYGGNAAT

AAAKSLSREFGSKLKEHMQYCADHPEEISKMAKLQAQVSELK

SAMIEKIEKVLDNKENVDVLVEKAKNLY

SQAQDFRLQGTTVRRNPWLQDM

>pda:103717265

MGQQTLIYSFVARGTVILAEFTEFTGNFTSIA

SQCLQKLPSSNNKFTYNCDSHTFNYLVEDGFT

YCVVAVESAGRQIPIAFLERVKEDFKKRYGGGKAAT

ASANSLNREFGSKLKEHMQYCVDHPEEISNLAKVKAQVSEVK

GVMMENIEKVLERGEKIELLVDKTENLR

SQAQDFRQQGTKIRRKMWFQNMKIKLIVLGIIIALILIIILSVCHGFK

CT

>pda:103695798

MGQQTLIYSFVARGAVILAEYTEFTGNFTSIA

SQCLQKLPASNNKFTYNCDSHTFNFLVEDGFT

YCVVAVESAGRQIPIAFLERVKEDFKKRYGGGKAAT

ASANSLNREFGPKLKEHMQYCVDHPEEISKLAKVKAEVSEVK

GVMMENIEKVLERGEKIELLVDKTENLR

SQAQDFRQHGTKMRRKMWLQNMKIKLIVLGIIIALILIIILSVCHGFK

CT

>pda:103720672

MGQQSLIYSFVARGTVILAEYTEFTGNFTSIA

SQCLQKLPASNNKFTYNCDGHTFNYLVEDGYT

YCVVAGESAGRQIPIAFLERIKEDFNKRYGGGKAAT

AAANGLNREFGPKLKEHMQYCVDHPEEISKLAKVKAQVSEVK

GVMMENIEKVLDRGEKIELLVDKTENLR

SQAQDFRQQGTKMRRRMWFQNMKIKLIVLGIIIALILIIILSVCHGFK

C

>pda:103706580

MSKQPLIYSFVAKGTVVLAEHTSFSGNFSTIA

VQCLQKLPPNSNKFTFSCDGHTFNFLVDKGFV

FLVVADESIGRSVPFVFLERVKDDFVQRYGSSIDDGGSHPLA

DDEDDDLFEDKFSIAYNLDREFGPRLKEHMQYCINHPEEMSKLSKLKAQITEVK

GIMMDNIEKVLDRGEKIELLVDRTESLQ

FQADSFQRQGRQLRRKMWLQNLQFKLMIAGAIIALILIVWLMACRGFK

C

>pda:103718871

MSKQPLIYSFVAKGTVVLAEHTSFSGNFNTIA

IQCLQKLTPNSNKYTYSCDGHMFNFLVDKGFV

FLVVTDESIGRSVPFVFLERVKDDFMQRYGSDINDGGFHTLA

DDEDDDLLEDRFSVAYSLDREFGPRLKEHMQYCVNHPEEMAKLSKLKSQITEVK

GIMMDNIEKVLDRGEKIELLVDKTESLQ

FQADSFQRHGRQLRRKMWLQNLRFKLMVAGAVMALIVIVWLMACRGFK

C

>pda:103715103

MGEGEEKGE

GEEKGWFIYSFVARGTMVLAEYTEYTGNFPAIA

AQCLQKLPSSNNKFTYACDHHTFNFLVHDGYA

YCVVAKESVPKHVSIAFLERLKADFKKRYGGGKADT

ATAKSLNKEFAPVIKEHMQYVIDHADEIEKLLKVKAQVSEVK

SIMLDNIDKAMDRGEKLKDLADKTTDLH

YQAQDFRKQGTKVRRKMWFQNMKIKLVVLGILLLLVLIVWVSVCQGFD

CTKHHN

>Bv_14430_kxzf

MAQQSLIYSFVARGTVILVEFTDFKGNFTSIA

AQCLQKLPSSNNKFTYNCDGHTFNYLVEDGFT

YCVVAVDSAGRQIPMSFLERVKEDFNKRYGGGKAAT

AQANSLNKEFGSKLKEHMQYCMDHPDEISKLAKVKAQVSEVK

GVMMENIEKVLDRGEKIELLVDKTENLR

SQAQDFRTTGTQMRRKMWLQNMKIKLIVLAIIIALILIIVLSVCHGFK

C
