# Supplementary material for: SNARE Complexity in Arbuscular Mycorrhizal Symbiosis
Source: Front Plant Sci. 2020 Apr 3;11:354. doi: 10.3389/fpls.2020.00354 (PMC7145992; doi:10.3389/fpls.2020.00354)
Supplement: Supplementary file 3 [file Table_1.docx]

| **Table S1. Interactions found between exocytosis related SNAREs in plants** | | | | | | | |
| --- | --- | --- | --- | --- | --- | --- | --- |
| **Syntaxin orthogroup** | **VAMP**  **Orthogroup** | | **Syntaxin** | **VAMP** | | **Assay** | **Reference** |
| SYP11I | VAMP72I | | AtSYP111 | AtVAMP721 | | Co-IP | (El Kasmi et al., 2013) |
| SYP12I | VAMP72I | | AtSYP121 | AtVAMP721 | | Pull-down | (Kwon et al., 2008) |
|  |  |  |  |  |  | Split luciferase | (Ichikawa et al., 2014) |
|  |  |  |  |  |  | Co-IP | (Ebine et al., 2011) |
|  |  |  |  |  |  | Pull-down | (Karnik et al., 2013) |
|  |  |  |  | AtVAMP722 | | Pull-down | (Kwon et al., 2008) |
|  |  |  |  |  |  | split luciferase | (Ichikawa et al., 2014) |
|  |  |  |  | AtVAMP721/AtVAMP722 | | Co-IP | (Kwon et al., 2008) |
|  |  |  | HvSYP121 | HvVAMP721 | | FRET-FLIM | (Kwon et al., 2008) |
|  |  |  |  |  |  | Split YFP | (Kwaaitaal et al., 2010) |
|  |  |  | AtSYP122 | AtVAMP722 | | Pull-down | (Pajonk et al., 2008) |
|  | VAMP72IV | | AtSYP121 | AtVAMP724 | | Pull-down | (Kwon et al., 2008) |
|  |  |  |  |  |  | split luciferase | (Ichikawa et al., 2014) |
|  | VAMP72VII | | AtSYP121 | AtVAMP727 | | pull down  Co-IP | (Kwon et al., 2008)  (Ebine et al., 2011) |
| SYP12IV | VAMP72I | | AtSYP123 | AtVAMP721 | | Pull-down,  Split luciferase | (Ichikawa et al., 2014) |
|  |  | |  | AtVAMP722 | | Pull-down,  Split luciferase | (Ichikawa et al., 2014) |
|  | VAMP72IV | | AtSYP123 | AtVAMP724 | | Pull-down,  Split luciferase | (Ichikawa et al., 2014) |
| SYP13I | VAMP72I | | AtSYP132 | AtVAMP721 | | Pull-down,  split luciferase | (Yun et al., 2013) |
|  |  |  |  |  |  | split luciferase | (Ichikawa et al., 2014) |
|  |  |  |  | AtVAMP722 | | Pull-down,  split luciferase | (Yun et al., 2013) |
|  |  |  |  |  |  | split luciferase | (Ichikawa et al., 2014) |
|  | VAMP72IV | |  | AtVAMP724 | | split luciferase | (Ichikawa et al., 2014) |
| **Absence of interaction found between exocytosis related SNAREs** | | | | | | | |
| **Syntaxin orthogroup** | **VAMP**  **Orthogroup** | **Syntaxin** | | | **VAMP** | **Assay** | **Reference** |
| SYP11I | VAMP72VII | AtSYP111 | | | AtVAMP727 | Co-IP | (Ebine et al., 2011) |
| SYP12I | VAMP72I | AtSYP121 | | | AtVAMP723  (ER/pseudogene) | split luciferase | (Ichikawa et al., 2014) |
| SYP12IV | VAMP72I | AtSYP123 | | | AtVAMP723  (ER/ pseudogene) | split luciferase | (Ichikawa et al., 2014) |
| SYP13I | VAMP72I | AtSYP132 | | | AtVAMP723  (ER/ pseudogene) | split luciferase | (Yun et al., 2013) |
|  |  |  |  |  |  | split luciferase | (Ichikawa et al., 2014) |

Ebine, K., Fujimoto, M., Okatani, Y., Nishiyama, T., Goh, T., Ito, E., et al. (2011). A membrane trafficking pathway regulated by the plant-specific RAB GTPase ARA6. *Nat. Cell Biol.* 13, 853–859. doi:10.1038/ncb2270.

El Kasmi, F., Krause, C., Hiller, U., Stierhof, Y.-D., Mayer, U., Conner, L., et al. (2013). SNARE complexes of different composition jointly mediate membrane fusion in Arabidopsis cytokinesis. *Mol. Biol. Cell* 24, 1593–601. doi:10.1091/mbc.E13-02-0074.

Ichikawa, M., Hirano, T., Enami, K., Fuselier, T., Kato, N., Kwon, C., et al. (2014). Syntaxin of plant proteins SYP123 and SYP132 mediate root hair tip growth in arabidopsis thaliana. *Plant Cell Physiol.* 55, 790–800. doi:10.1093/pcp/pcu048.

Karnik, R., Grefen, C., Bayne, R., Honsbein, A., Köhler, T., Kioumourtzoglou, D., et al. (2013). Arabidopsis Sec1/Munc18 protein SEC11 Is a competitive and dynamic modulator of SNARE binding and SYP121-dependent vesicle traffic. *Plant Cell* 25, 1368–1382. doi:10.1105/tpc.112.108506.

Kwaaitaal, M., Keinath, N. F., Pajonk, S., Biskup, C., and Panstruga, R. (2010). Combined bimolecular fluorescence complementation and Forster resonance energy transfer reveals ternary SNARE complex formation in living plant cells. *Plant Physiol.* 152, 1135–1147. doi:10.1104/pp.109.151142.

Kwon, C., Bednarek, P., and Schulze-Lefert, P. (2008). Secretory pathways in plant immune response. *Plant Physiol.* 147, 1575–1583. doi:10.1104/pp.108.121566.

Pajonk, S., Kwon, C., Clemens, N., Panstruga, R., and Schulze-Lefert, P. (2008). Activity determinants and functional specialization of Arabidopsis PEN1 syntaxin in innate immunity. *J. Biol. Chem.* 283, 26974–26984. doi:10.1074/jbc.M805236200.

Yun, H. S., Kwaaitaal, M., Kato, N., Yi, C., Park, S., Sato, M. H., et al. (2013). Requirement of vesicle-associated membrane protein 721 and 722 for sustained growth during immune responses in Arabidopsis. *Mol. Cells*, 1–8. doi:10.1007/s10059-013-2130-2.

**Table S2.** Primers used in this study.

|  | **number** | **name** | **Sequence 5’🡪3’** |
| --- | --- | --- | --- |
| ddPRC/qPCR | 1 | SYP111F | TCCCATTGTCACTAGTTTCAGC |
|  | 2 | SYP111R | GGCAATTCAAACGACGATG |
|  | 3 | SYP121F | ATGCAGCAGCTATTCCCATC |
|  | 4 | SYP121R | TTCACAAGGTTTGCCTCACC |
|  | 5 | SYP122F | AGCGAGGAAGAACAAGATCAG |
|  | 6 | SYP122R | CCGATTGTAACCCTTGTTCC |
|  | 7 | SYP123F | TCTCAAATGGTGGCTACTGG |
|  | 8 | SYP123R | TGATGCTTGACTTGACTACCAAG |
|  | 9 | SYP131F | CGGTAACACATTGTTTCCACAAC |
|  | 10 | SYP131R | GAGACCAATGTTTGTTTCAACC |
|  | 11 | SYP132AF | CAGCAGTTTAACAAGGTTCATGG |
|  | 12 | SYP132AR | AATATGCACAGAAAGCCAATTC |
|  | 13 | SYP132BF | GTTGTGTTGGTTGGGAAAGC |
|  | 14 | SYP132BR | CAGAGACACACCGATGTATTAGC |
|  | 15 | V721AF | AGGAAGGGAAGTGAATAAGGTGTG |
|  | 16 | V721AR | AGCATGCTTTCTATACACAATTCC |
|  | 17 | V721DF | TTGTAAATTGTTTGCTTTGCG |
|  | 18 | V721DR | AAAAAGCCACAGGTCCAATC |
|  | 19 | V721EF | AAGCTTTTACAGTATGATGGTGATG |
|  | 20 | V721ER | ATGATAACAGGATGGGTCGG |
|  | 21 | V724F | AAGAAAATCAACCCAGGAGGG |
|  | 22 | V724R | CTTTATCCACCAGCAACAAGG |
|  | 23 | V727F | TTGTACATTGAATTTGGTGCG |
|  | 24 | V727R | CTTGATTCAAAGTGACCAGCAG |
|  | 25 | Actin2F | CAGATGTGGATCTCCAAGGGTGA |
|  | 26 | Actin2R | TGACTGAAATATGGCACAAGACTGAGA |
|  | 27 | Ub10F | CCCTTCATCTTGTCCTTCGTCTG |
|  | 28 | Ub10R | CACCTCCAATGTAATGGTCTTTCC |
| Cloning cds SYP131 | 29 | cdsSYP131F | CACCATGAATGATCTTCTAACGGAATCA |
|  | 30 | cdsSYP131R | CTATGCACCCTTTTTGGTAACC |
| Cloning cds VAMP724 | 31 | cdsVAMP724F | CACCATGAGTCAAGAATCGTTCATATACAGC |
|  | 32 | cdsVAMP724R | CTAATTTGAACAGTTAAATCCACCG |
| Cloning cds VAMP727 | 33 | cdsVAMP727F | CACCATGAGTCAAAGGGGTTTGATATAT |
|  | 34 | cdsVAMP727R | TCAACATTTGAAACCCCCAC |
| PCR expression cassettes | 35 | MGWSpeI_F | TGACTAGTACGCCAAGCTATCAACTTTGT |
|  | 36 | MGWSwaI_R | GCATTTAAATCACGACGGCCAGTGAAT |
|  | 37 | MGWSwaI_F | TGATTTAAATACGCCAAGCTATCAACTTTGT |
|  | 38 | MGWApaI_R | TCATAACGTGACTCCCTTAATTCTC |
|  | 39 | MGWApaI_F | TAGGGCCCTACGCCAAGCTATCAACTTTGT |
|  | 40 | MGWEco81I_R | AACCTTAGGTCACGACGGCCAGTGAAT |
| Split-GFP vector construction | 41 | AscI-GFPnF | AGGCGCGCCATGGTGAGCAAGGGCGAG |
|  | 42 | Acc65I-GFPnR | AGGTACCGGCCATGATATAGACGTTGTG |
|  | 43 | AscI-START-GFPcF | AGGCGCGCCATGGACAAGCAGAAGAACGGCAT |
|  | 44 | Acc65I-GFPcR | AGGTACCCTTGTACAGCTCGTCCATGC |
| Cloning SYP132α CRISPR construct | 45 | SG-F | CACCGTCGACGTAAAGCCTGTAGAAGA |
|  | 46 | SYP132α-U6-R | GACTTCACATGATCAGTTGCAATCACTACTTCGACTCTAGCTGT |
|  | 47 | SYP132α-SG-F | GCAACTGATCATGTGAAGTCGTTTTAGAGCTAGAAATAGCAAGTTA |
|  | 48 | SG-R | AAAAGGTACCAAAAATTATATCCTGTG |
| detection SYP132α CRISPR deletion | 49 | SYP132α-F1 | GGCTTTTGGAGCAATACGGG |
|  | 50 | SYP132α-R1 | GTAAGTTGTGCCACCGTTCG |
| Cas9 detection | 51 | Cas9-F | TGGAGCAGCACAAGCACTACC |
|  | 52 | Cas9-R | CAAGACCGGCAACAGGATTCA |
| Cloning Timer-NLS | 53 | RikH_timer_F | CACCATGGTGGCTTCCTCCGA |
|  | 54 | RikH_NLS_R | TTAGGCAACCTTTCTCTTCTTCTT |
| Cloning Timer | 55 | AscI-TimerF | GGCGCGCCATGGTGGCTTCCTCCGAAGA |
|  | 56 | KpnI-TimerR | GGTACCCAGGAACAGGTGGTGGCGGCC |
