# Supplementary figures and images for: SNARE Complexity in Arbuscular Mycorrhizal Symbiosis
Source: Front Plant Sci. 2020 Apr 3;11:354. doi: 10.3389/fpls.2020.00354 (PMC7145992; doi:10.3389/fpls.2020.00354)

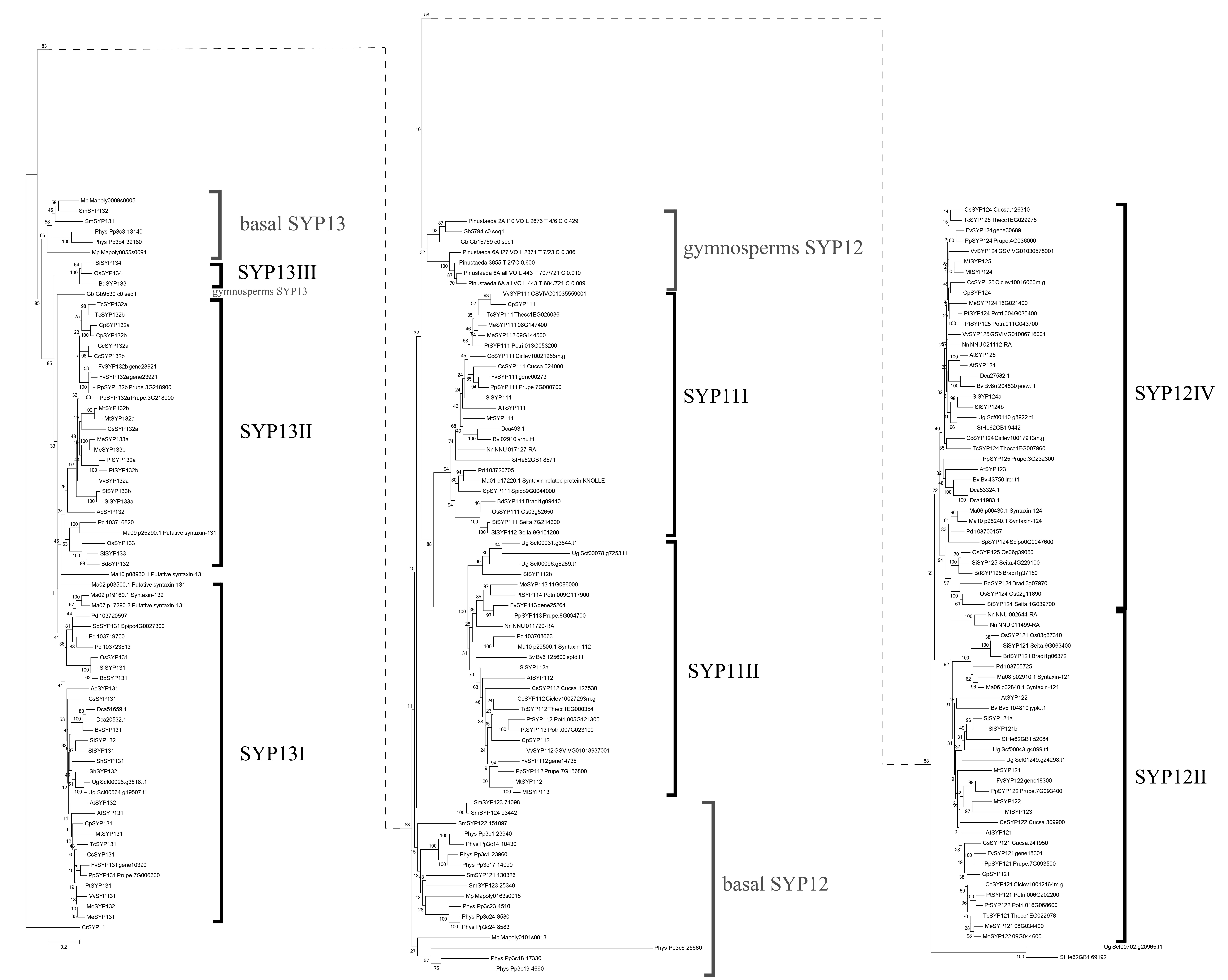

Supplement: Supplementary file 4 [file Image_1.JPEG]

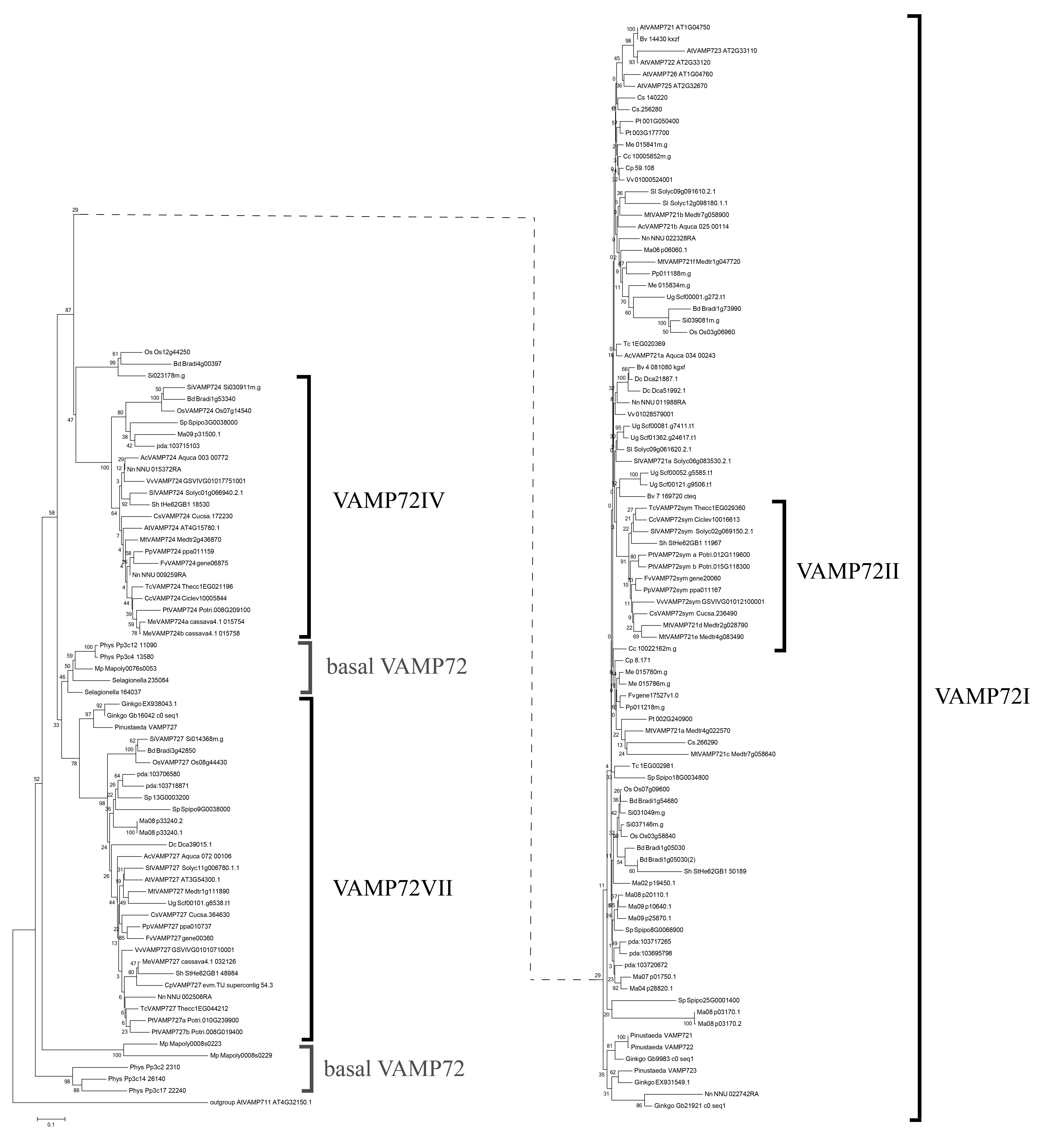

Supplement: Supplementary file 5 [file Image_2.JPEG]

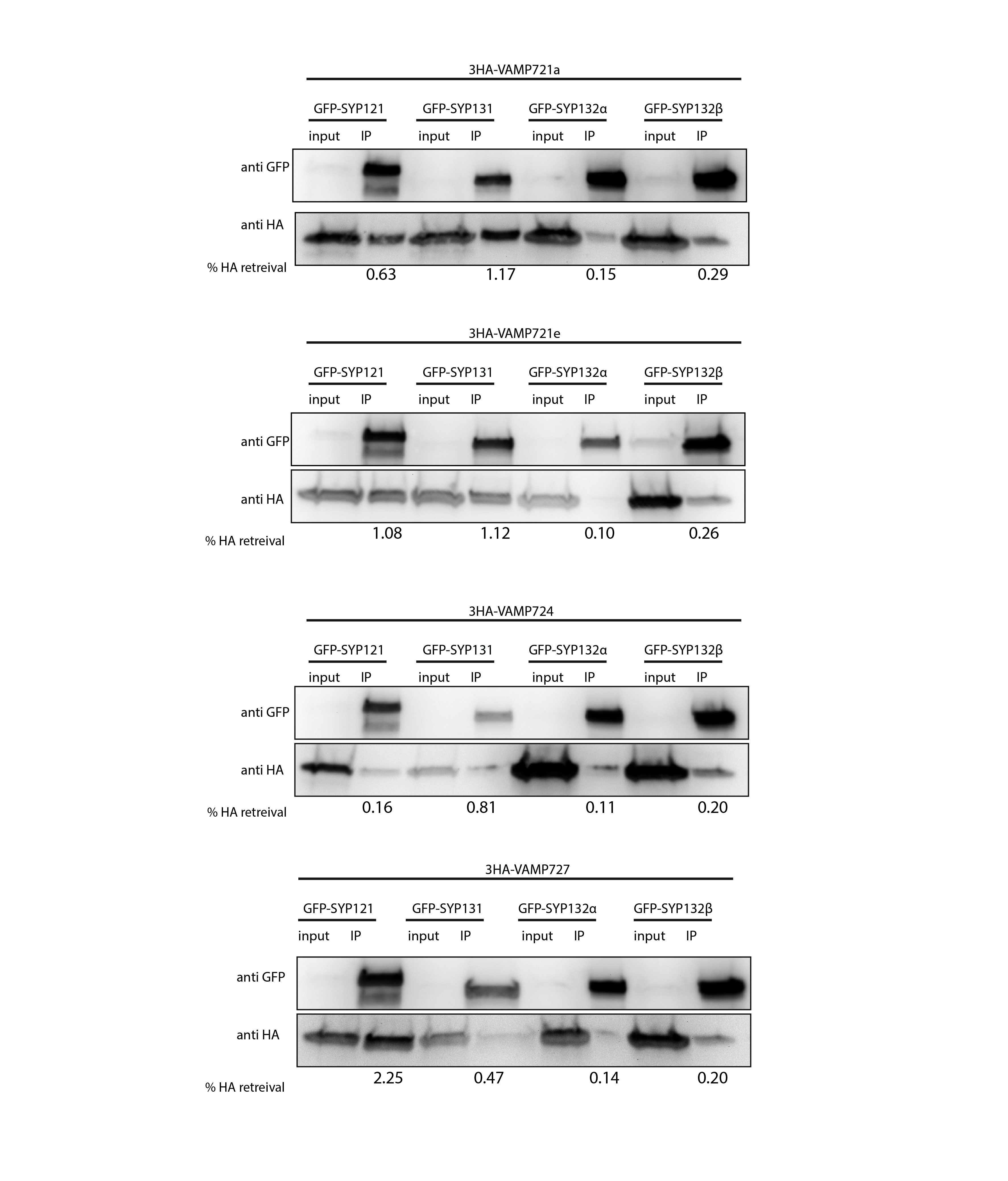

Supplement: Supplementary file 6 [file Image_3.JPEG]

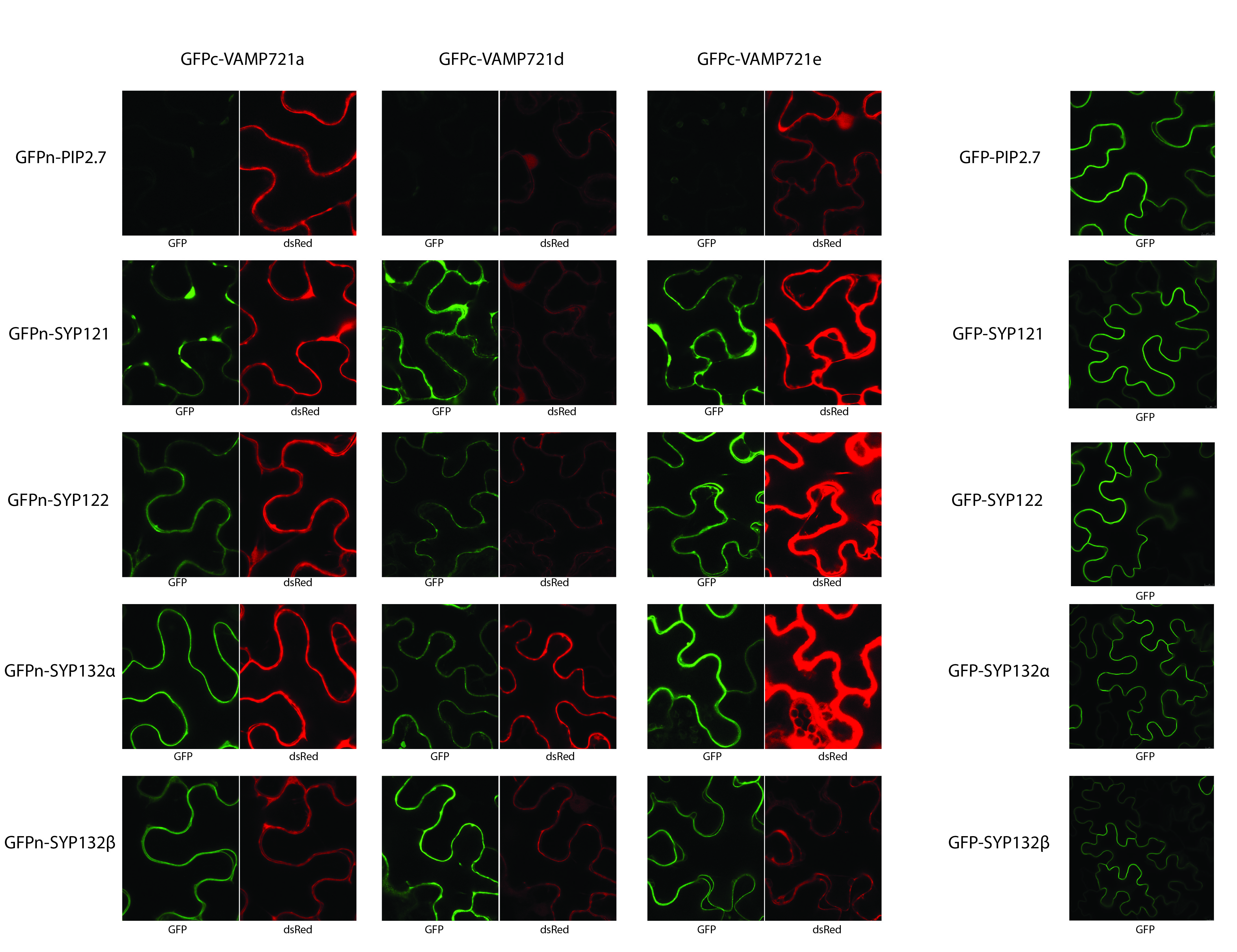

Supplement: Supplementary file 7 [file Image_4.JPEG]

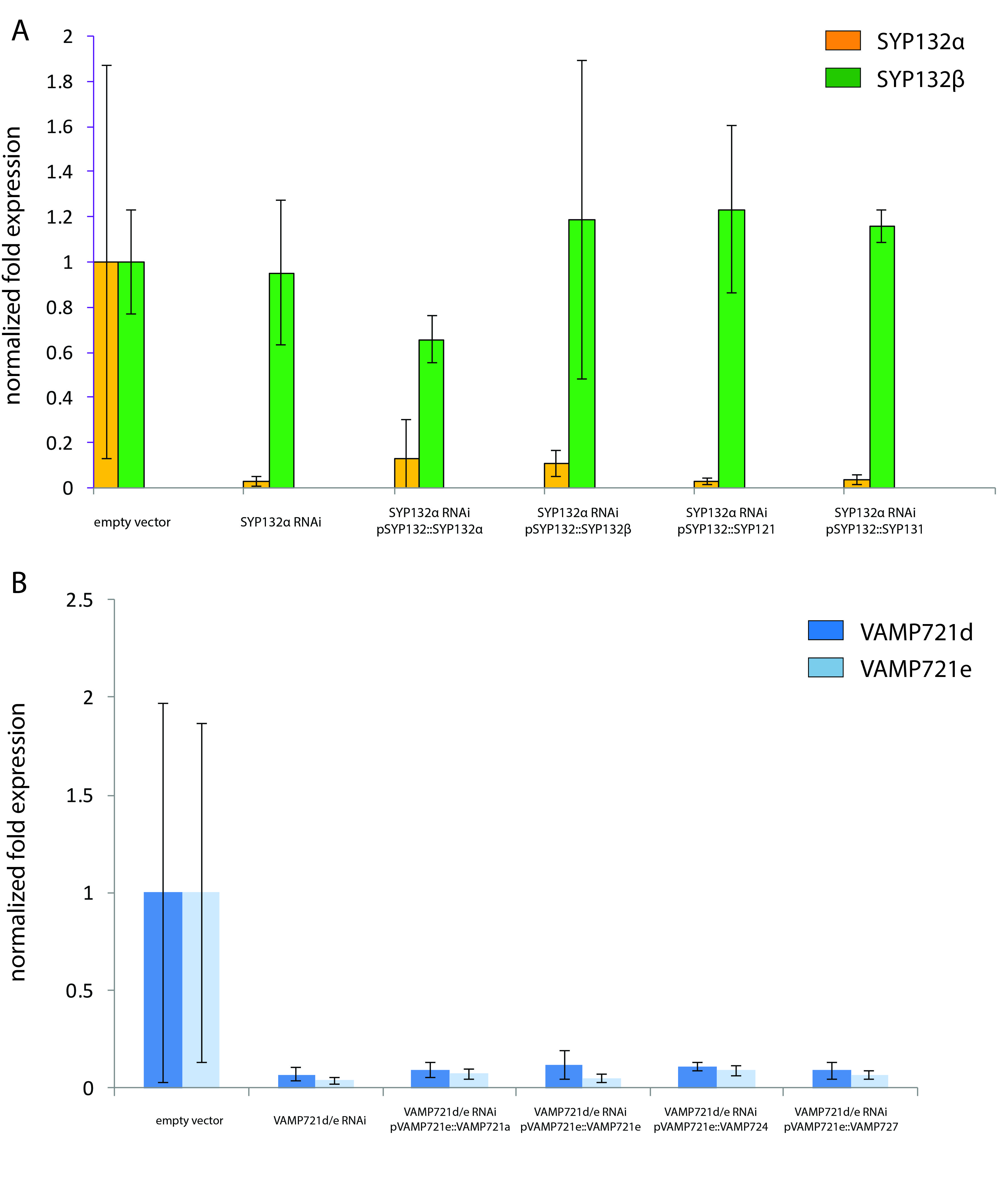

Supplement: Supplementary file 8 [file Image_5.JPEG]

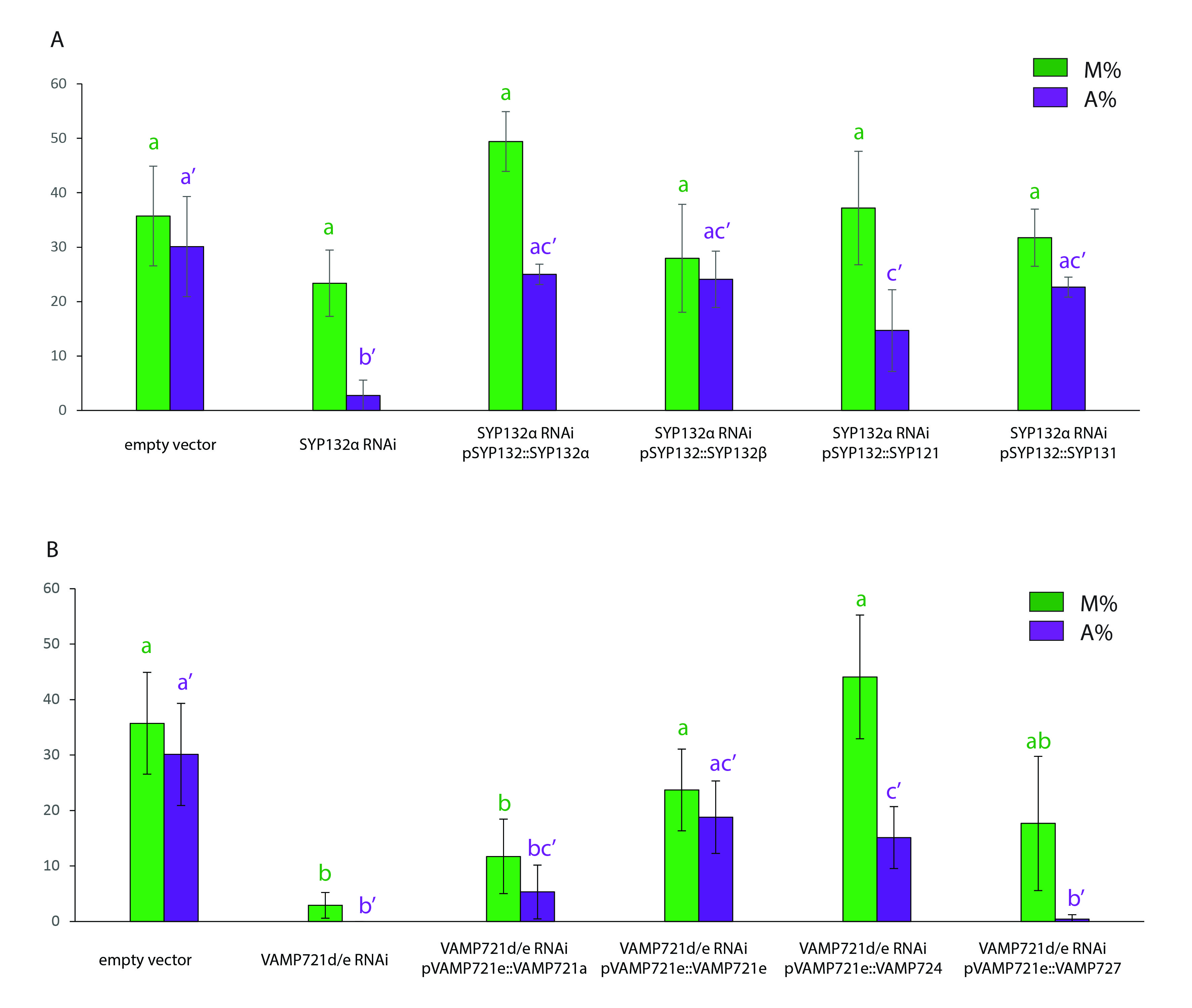

Supplement: Supplementary file 9 [file Image_6.JPEG]

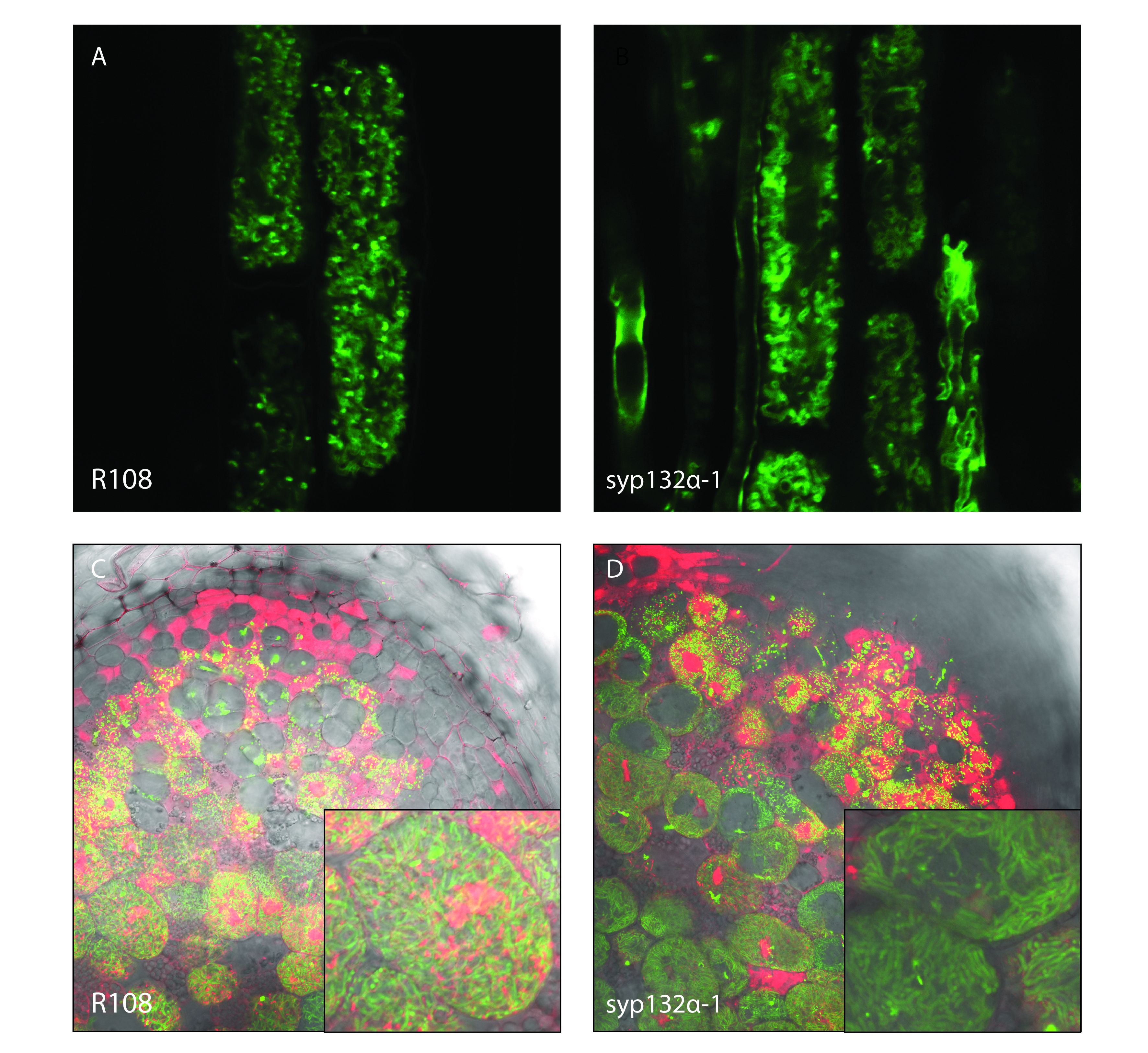

Supplement: Supplementary file 10 [file Image_7.JPEG]

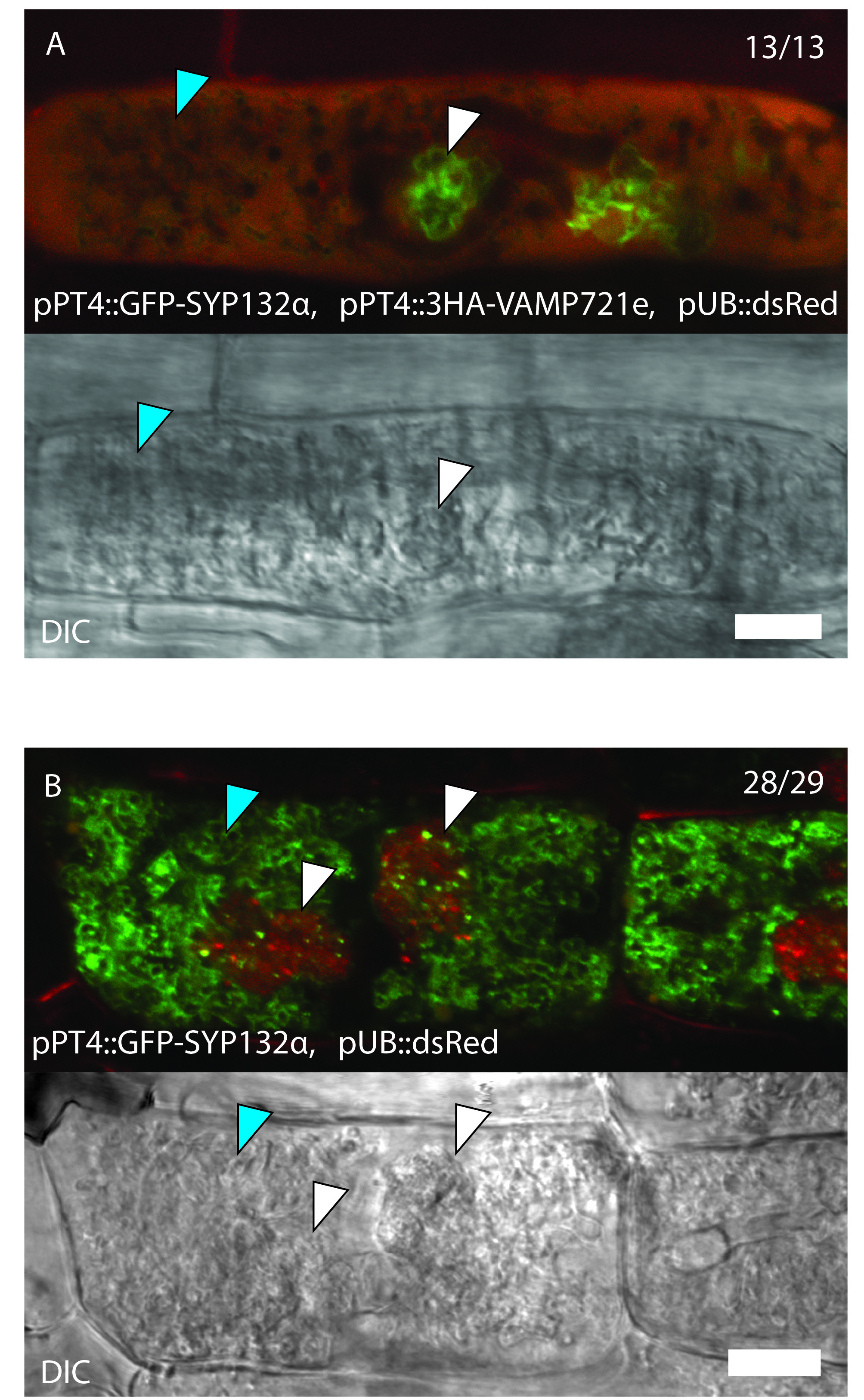

Supplement: Supplementary file 11 [file Image_8.JPEG]
